# Post-Synthetic Modification of a Metal–Organic Framework Glass

**DOI:** 10.1021/acs.chemmater.1c03820

**Published:** 2022-02-18

**Authors:** Alice M. Bumstead, Ignas Pakamorė, Kieran D. Richards, Michael F. Thorne, Sophia S. Boyadjieva, Celia Castillo-Blas, Lauren N. McHugh, Adam F. Sapnik, Dean S. Keeble, David A. Keen, Rachel C. Evans, Ross S. Forgan, Thomas D. Bennett

**Affiliations:** † Department of Materials Science and Metallurgy, University of Cambridge, Cambridge CB3 0FS, U.K.; ‡ WestCHEM, School of Chemistry, The University of Glasgow, University Avenue, Glasgow G12 8QQ, U.K.; § Diamond Light Source Ltd, Diamond House, Harwell Campus, Didcot, Oxfordshire OX11 0DE, U.K.; ∥ ISIS Facility, Rutherford Appleton Laboratory, Harwell Campus, Didcot, Oxfordshire OX11 0QX, U.K.

## Abstract

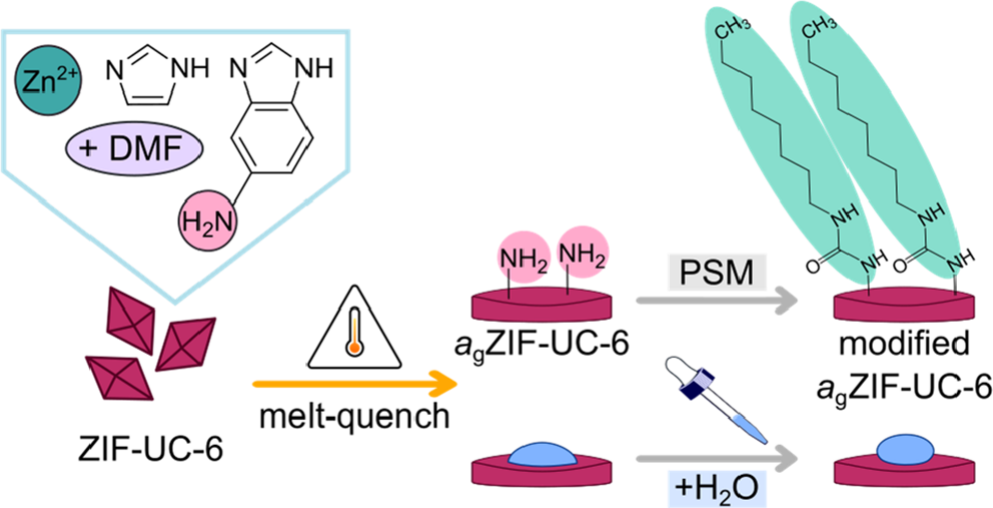

Melt-quenched metal–organic framework (MOF) glasses have gained significant interest as the first new category of glass reported in 50 years. In this work, an amine-functionalized zeolitic imidazolate framework (ZIF), denoted ZIF-UC-6, was prepared and demonstrated to undergo both melting and glass formation. The presence of an amine group resulted in a lower melting temperature compared to other ZIFs, while also allowing material properties to be tuned by post-synthetic modification (PSM). As a prototypical example, the ZIF glass surface was functionalized with octyl isocyanate, changing its behavior from hydrophilic to hydrophobic. PSM therefore provides a promising strategy for tuning the surface properties of MOF glasses.

## Introduction

There is growing interest in the field of metal–organic frameworks (MOFs) toward material properties such as framework flexibility and stimuli-induced structural transitions.^1−3^ Such transformations can be induced by chemical inclusion such as gas sorption, or by physical stimuli including pressure, temperature, or UV light.^3,4^ It has recently been observed that temperature can induce a solid–liquid transition in several MOFs.^5,6^ Cooling of these liquids can yield melt-quenched glasses, which retain the local metal–ligand connectivity of the parent crystalline framework, though they lack any long-range periodicity.^7^ These melt-quenched glasses retain some porosity in the glass phase, and, as they pass through a liquid state, can be more easily processed compared to typical crystalline powders.^6^

Melting and glass formation in MOFs are most commonly observed in zeolitic imidazolate frameworks (ZIFs), a subset composed of tetrahedral metal ions coordinated to imidazolate (Im—C_3_H_3_N_2_^–^)-type linkers.^7−9^ Melting in ZIFs has been demonstrated computationally to be a dynamic process occurring on a picosecond timescale; de-coordination of an imidazolate linker is rapidly followed by re-coordination of a new linker in its place.^6^ The reversible transition when a liquid is frozen as an amorphous solid, that is, without crystallization, is known as the glass transition (occurring at temperature *T*_g_).^7,10^

A reduction of the melting temperature (*T*_m_) and *T*_g_ of ZIFs would facilitate large-scale material processing at lower temperatures, improve the optical quality of the glasses, and prevent decomposition-related discoloration.^11−13^ Strategies designed to reduce *T*_m_ in ZIFs typically involve changing the chemical functionality of the crystalline framework either at the metal center, such as by replacing the more usual Zn^2+^ with Co^2+^, or at the organic linker, by using various mixed-linker approaches.^11,12^ Highly disordered systems such as [Co_0.2_Zn_0.8_(Im)_1.95_(bIm)_0.025_(ClbIm)_0.025_] (bIm—benzimidazolate—C_7_H_5_N_2_^–^, ClbIm—5-chlorobenzimidazolate—C_7_H_4_N_2_Cl^–^) have been found to exhibit melting temperatures of *ca.* 310 °C, that are among the lowest currently known for ZIFs.^10^ The use of electron-withdrawing linkers to lower both *T*_m_ and *T*_g_ by weakening the Zn–N coordination bond has also been reported.^14,15^ For example, the ClbIm linker in ZIF-UC-5 [Zn(Im)_1.8_(ClbIm)_0.2_] resulted in a lower *T*_m_ (428 °C) and *T*_g_ (336 °C) compared to a non-halogenated structural isomorph [Zn(Im)_1.8_(mbIm)_0.2_] (mbIm—5-methylbenzimidazolate—C_8_H_7_N_2_^–^) (*T*_m_ = 440 °C, *T*_g_ = 350 °C).^14^

Amine-functionalized ZIF glasses are interesting due to their potential adsorption capability for Lewis acidic gases such as CO_2_,^16−18^ the hydrophilic nature imparted to the glasses by the amine moiety, and the possibility for post-synthetic modification (PSM) of these ZIF glasses to further tune their properties.^19−21^ Given these advantages, it is surprising that there have only been a few reports of amine-functionalized crystalline ZIFs^16,17^ and no reports of any ZIF glasses containing this functionality.^22−24^

Currently, the chemical functionality available within ZIF glasses remains limited.^14,15^ In the inorganic glass domain, chemical modification is achieved through post-synthetic ion exchange of Na^+^ ions in sodium silicate glasses for larger K^+^ ions. This is used to strengthen the glass surface, resulting in toughened glasses, suitable for smartphone screens.^25,26^ Other methods include ion implantation, where the glass surface is bombarded with high energy ions, altering the surface chemistry and hence optical and mechanical properties, such as refractive index and material hardness.^27,28^ Inorganic glasses have also been modified with polymer coatings to enhance both the hydrophobicity and durability of the glass surface.^29,30^ PSM is also used to produce porous inorganic glass, for example, Vycor glass (Corning Incorporated), which is prepared by post-synthetic treatment of a melt-quenched inorganic glass with acid to remove the boron and alkali metal-rich components.^31,32^

PSM of crystalline MOFs has also been demonstrated, utilizing reactive chemical functionalities included in their frameworks.^19−21^ For example, the nucleophilic amine functionality on 2-aminoterephthate in IRMOF-3 has been reacted with acetic anhydride, resulting in an amide-functionalized framework.^33^ Further studies demonstrated the versatility of amine functionalities for reacting with various electrophiles including carboxylic acids, acid anhydrides, and isocyanates.^34−36^ Despite this, PSM has not been demonstrated on melt-quenched MOF glasses or indeed on any noncrystalline MOF system. This raises two questions: is it achievable, and if it is, what effect would PSM have on the physical and chemical properties of the glass?

**Figure 1:**
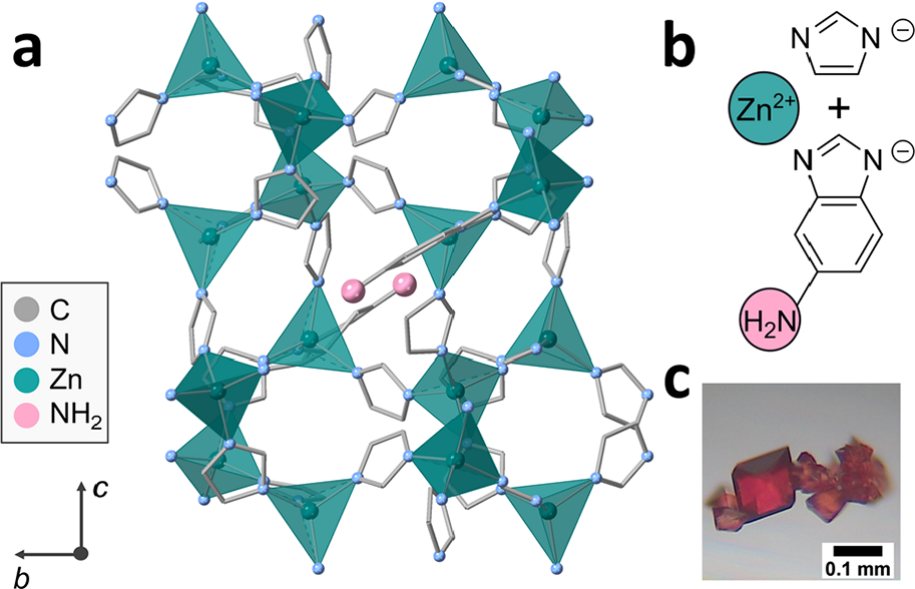
(a) Crystal structure of ZIF-UC-6, *Pbca* space group, **cag** topology, viewed down the crystallographic *a* axis. Atoms shown are carbon (gray), nitrogen (blue), and zinc (green). ZnN_4_ tetrahedra have been highlighted in green, amine groups have been highlighted in pink, while hydrogen atoms have been omitted for clarity. Disorder resulting from multiple linker occupancy has also been omitted for clarity. (b) Zn^2+^ ion alongside imidazolate and aminobenzimidazolate linkers in ZIF-UC-6. (c) Optical image of dark burgundy single crystals of ZIF-UC-6.

Here, we synthesize a previously unknown amine-functionalized ZIF, denoted ZIF-UC-6, possessing the **cag** network topology (Figure 1). We first demonstrate its liquid and glass-forming behavior before utilizing the amine functionality to investigate PSM on both crystalline ZIF-UC-6 and its glass, denoted *a*_g_ZIF-UC-6. This allowed us to investigate the effect of PSM on the physical properties of a ZIF glass for the first time.

## Results and Discussion

### Crystalline ZIF-UC-6

Single crystals of ZIF-UC-6 were grown by solvothermal synthesis (Methods). Specifically, zinc nitrate hexahydrate (0.32 mmol), imidazole (6.76 mmol), and 5-aminobenzimidazole (abIm, 0.75 mmol) were dissolved in *N*,*N*-dimethylformamide (DMF), yielding a dark red solution which was heated to 130 °C and held there for 48 h (Figures S1, S2). Slow cooling of the reaction mixture at 5 °C h^–1^ resulted in burgundy single crystals (Figure S3). Single-crystal X-ray diffraction (SCXRD) confirmed their crystallization in the *Pbca* space group [*a* = 15.839(2) Å, *b* = 15.599(2) Å, *c* = 17.984(2) Å, *V* = 4443.3(9) Å^3^] (Figures 1, S4, Table S1). ZIF-UC-6 crystallizes with a unit cell very similar to that of the prototypical glass-former ZIF-62 [Zn(Im)_2–*x*_(bIm)_*x*_].^11,37^ The abIm ligand is localized on one of the four possible linker positions; a 0.4:0.6 abIm:Im occupancy ratio on this site leads to an overall composition of [Zn(Im)_1.82_(abIm)_0.18_] for this particular crystal. A phase-pure burgundy microcrystalline powder sample was then synthesized (Methods, Figures S5–S7, Table S2) with a composition of [Zn(Im)_2–*x*_(abIm)_*x*_] (where *x* = 0.18) confirmed by ^1^H NMR spectroscopy (Figure S8), correlating closely to the single-crystal structure which can be taken as representative of the bulk polycrystalline powder.

### Thermal Behavior and Glass Formation

**Figure 2:**
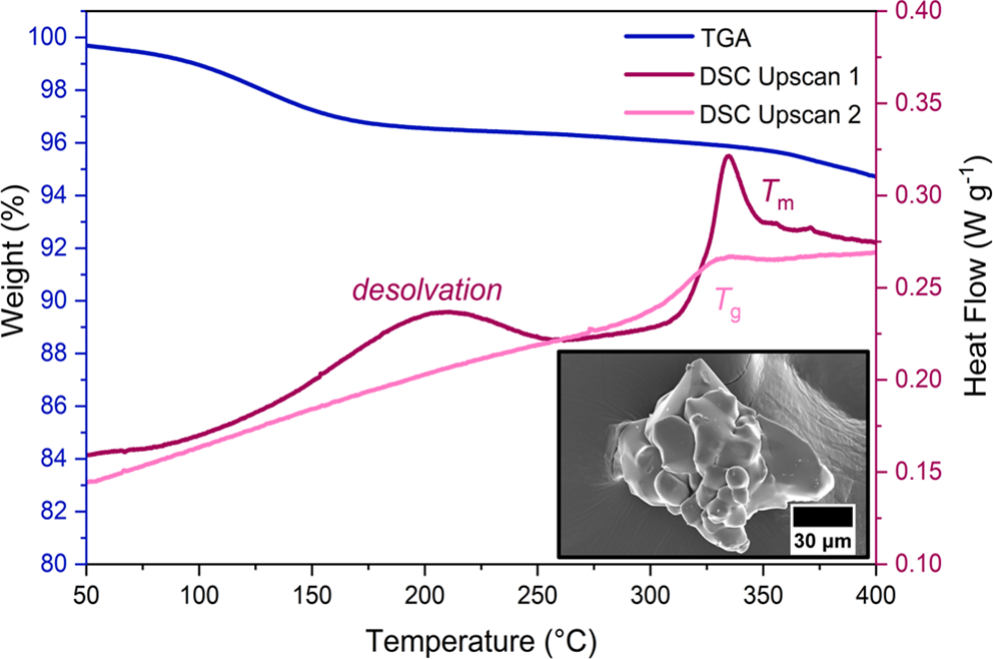
TGA curve (first upscan—blue) and DSC traces (first upscan—dark pink; second upscan—light pink) of ZIF-UC-6 in the region of 50–400 °C. The first upscan of the DSC exhibits a desolvation endotherm at 211 °C followed by a melting endotherm. *T*_m_ was taken as the offset of this endotherm with *T*_m_ = 345 °C. The second DSC upscan shows a *T*_g_ of 316 °C. The inset shows an SEM image of *a*_g_ZIF-UC-6 displaying a loss of crystal facets and evidence of particle coalescence and flow.

The thermal response of ZIF-UC-6 was studied using differential scanning calorimetry (DSC) and thermogravimetric analysis (TGA) (Figures 2, S9–S11). A 3.5% mass loss occurred below 220 °C and was accompanied by a broad endotherm in the DSC with a peak at 211 °C. This is ascribed to residual solvent in the pores after activation in line with previous studies.^13^ Above 507 °C, significant sample decomposition began as indicated by a sharp drop in mass (Figure S9). Below this temperature, a melting endotherm was observed in the DSC (*T*_m_ = 345 °C) (Figures 2, S11). A 1.8% mass loss occurred between desolvation and 400 °C, suggesting that melting is accompanied by trace levels of decomposition (Figure S10). Reheating this sample gave a *T*_g_ = 316 °C (Figures 2, S11). Samples of ZIF-UC-6 heated above 400 °C and subsequently cooled will henceforth be denoted *a*_g_ZIF-UC-6 to highlight their transformation to the glass phase.

ZIF-UC-6 melts at a markedly lower temperature than other isostructural—*Pbca* space group, **cag** topology—melting ZIFs with different functionalized linkers such as TIF-4 [Zn(Im)_1.8_(mbIm)_0.2_] (mbIm—5-methylbenzimidazolate—C_8_H_7_N_2_) (*T*_m_= 440 °C) and ZIF-UC-5 [Zn(Im)_1.8_(ClbIm)_0.2_] (ClbIm—5-chlorobenzimidazolate—C_7_H_4_N_2_^–^Cl^–^) (*T*_m_ = 428 °C).^14^ A recent study highlighted that *T*_m_ has both an enthalpic contribution (Δ*H*_fus_) and an entropic contribution (Δ*S*_fus_), and that lowering Δ*H*_fus_ and increasing Δ*S*_fus_ both result in a reduction of *T*_m_.^38^ We applied this methodology here and compared the enthalpic and entropic contributions to melting in ZIF-UC-6 to those reported for TIF-4 and ZIF-UC-5 (Table S3, Figure S12).^14^ ZIF-UC-6 had the lowest Δ*H*_fus_ and Δ*S*_fus_ of the three ZIFs compared. Additionally, both Δ*H*_fus_ and Δ*S*_fus_ increased from ZIF-UC-6 to ZIF-UC-5 to TIF-4, following the same trend as *T*_m_. This implies that the major contribution to *T*_m_ is Δ*H*_fus_, and that the low *T*_m_ of ZIF-UC-6 results from weaker Zn–N coordination bonds.

The amine functionality in ZIF-UC-6 also has the potential to undergo hydrogen bonding. Therefore, one possible explanation for the low *T*_m_ of ZIF-UC-6 is that the dynamic movement of the framework during melting may result in the potential formation of temporary hydrogen bonding interactions. These dispersive interactions may stabilize the uncoordinated linkers that form during the melting process, thus reducing the energy barrier to melting.^6^

The *T*_g_ for *a*_g_ZIF-UC-6 (*T*_g_ = 316 °C) is also lower when compared to these other two ZIF glasses: *a*_g_TIF-4 (*T*_g_ = 350 °C) and *a*_g_ZIF-UC-5 (*T*_g_ = 336 °C). This may also be attributed to the lower Δ*H*_fus_, that is, weaker coordination bonds, in ZIF-UC-6 compared to these frameworks. Additionally, the Van der Waals volume of −NH_2_ is 10.54 cm^3^ mol^–1^, while −CH_3_ in TIF-4 (13.67 cm^3^ mol^–1^) and −Cl in ZIF-UC-5 (12.0 cm^3^ mol^–1^) are both larger.^39,40^ The *T*_g_ also depends on steric freedom, that is, how easily molecules can move past one another; so the smaller linker substituent in *a*_g_ZIF-UC-6 also likely contributes to its lower *T*_g_.^7,41^ The use of amine-functionalized linkers therefore provides a promising strategy for preparing ZIFs with a lower *T*_m_ and *T*_g_.

A sample of *a*_g_ZIF-UC-6 quenched from 400 °C appeared visually to have undergone melting, with evidence of particle coalescence as well as the presence of flow-related striations (Figure 2 inset, Figure S13). This sample was then confirmed to be X-ray amorphous with a composition identical to the parent crystalline material, that is, [Zn(Im)_1.82_(abIm)_0.18_] (Figures S5, S14). The thermal stability of the glass was found to be very similar to the crystalline material (Figure S15, S16).

**Figure 3:**
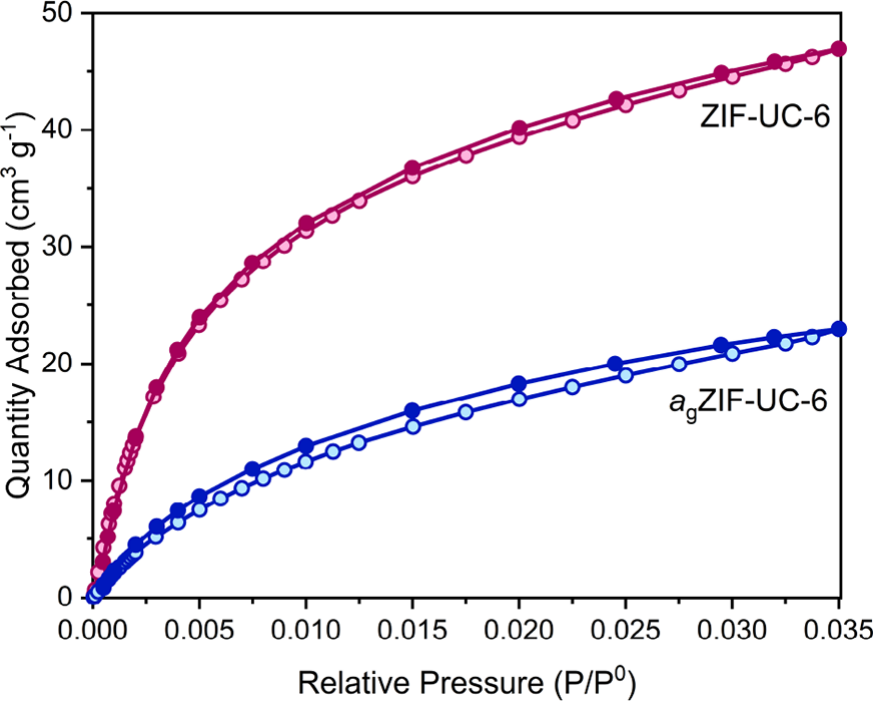
CO_2_ gas sorption isotherms for ZIF-UC-6 (pink) and *a*_g_ZIF-UC-6 (blue). Adsorption isotherms are represented by open circles, while desorption isotherms are represented by closed circles. ZIF-UC-6 had a maximum uptake of 46.9 cm^3^ g^–1^ at STP, while for *a*_g_ZIF-UC-6, it was 23.0 cm^3^ g^–1^ at STP.

CO_2_ gas sorption on both crystalline ZIF-UC-6 and *a*_g_ZIF-UC-6 revealed significant differences in their behavior. ZIF-UC-6 exhibited a CO_2_ uptake of 46.9 cm^3^ g^–1^ at standard temperature and pressure (STP) (Figures 3, S17, Table S4), with this value being comparable to some of the highest values reported for CO_2_ uptake in ZIFs.^15,23,42^ Meanwhile, *a*_g_ZIF-UC-6 displayed a lower CO_2_ uptake (23.0 cm^3^ g^–1^ at STP) as well as more pronounced hysteresis, as expected due to the collapse of the crystalline framework upon glass formation (Figures 3, S18, Table S5).

**Figure 4:**
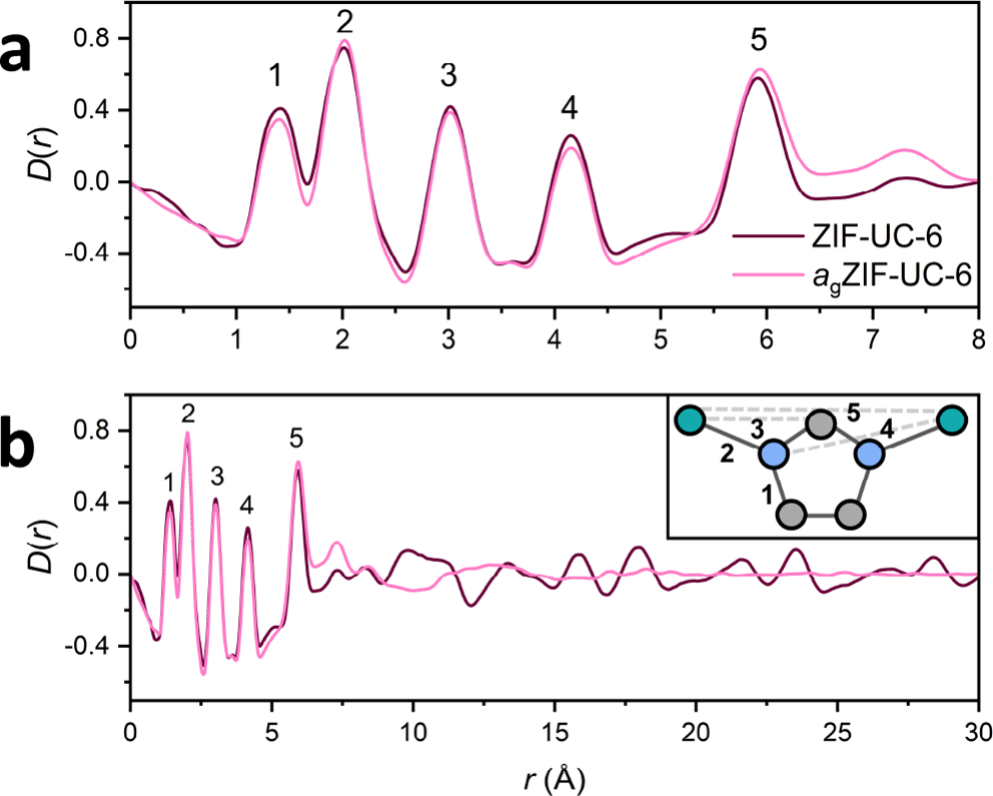
PDF, *D*(*r*), of ZIF-UC-6 (dark pink) and *a*_g_ZIF-UC-6 (light pink). (a) Short range order correlations (0–8 Å) undergo minimal changes on glass formation. (b) Full *D*(*r*) shows long range order (>8 Å) is lost after heating. The inset shows molecular connectivity along with some of the key corresponding correlations. The five dominant correlations at low-*r* are also labeled on the *D*(*r*). Atoms shown are carbon (gray), nitrogen (blue), and zinc (green). Hydrogen atoms have been omitted for clarity.

To further investigate the structures of ZIF-UC-6 and *a*_g_ZIF-UC-6, X-ray total scattering data were collected on the I15-1 beamline at the Diamond Light Source, (Methods, Figure S19). Fourier transformation of the corrected total scattering data gave the pair distribution function (PDF), which we have presented here in the *D*(*r*) form (Figure 4). Short-range order correlations of ZIF-UC-6 (labeled 1–5) were maintained in *a*_g_ZIF-UC-6 with little variation in the intensities of these peaks, implying that chemical connectivity is largely retained in the glass phase. However, long-range order (>8.0 Å) was lost in *a*_g_ZIF-UC-6, supporting the lack of long-range periodicity in the glass phase (Figure 4b).

### PSM of *a*_g_ZIF-UC-6

The use of PSM to further tune the properties of a MOF glass has yet to be reported, despite the potential to alter its thermal and surface properties. PSM of crystalline MOFs has already been studied in considerable depth,^19,43^ with the use of amine-functionalized linkers being a common choice as the site for further reaction.^34−36^ The amine functionality on the abIm linker in both ZIF-UC-6 and *a*_g_ZIF-UC-6 therefore provides a promising target for further modification with various electrophiles.

Anhydrides are frequently used for PSM of amine-functionalized MOFs, as they are highly reactive electrophiles, resulting in amide-functionalized frameworks alongside a carboxylic acid byproduct.^19,34^ However, although ZIFs are well known for their thermal and chemical stability, their susceptibility to acid digestion is well reported.^8,44^ As such, an alternative electrophile was needed for ZIF-UC-6. Isocyanates are also reported to react with amine-functionalized frameworks, yielding urea functionalities and no byproducts (Figure S20).^35,36^ These reasons, in addition to the desire to ultimately alter the surface hydrophobicity of the glass, led to octyl isocyanate being selected as the modification reagent, as it combined both an electrophilic functionality with a hydrophobic carbon tail (Figure S21).

The low amine content in ZIF-UC-6 meant that only 9% of the linkers contained the reactive amine group necessary for PSM. Furthermore, the small pore size within ZIF-UC-6 (<6 Å) meant only surface modification would be possible, as octyl isocyanate (*ca.* 13 Å) (Figure S22) would be too large to enter the pores of the framework.^45^ This provided an additional challenge compared to previous studies which have generally focused on materials where all of the linkers were accessible for PSM.^19,34^

There appeared to be two possible strategies to prepare a hydrophobic ZIF glass by PSM: (i) perform PSM on crystalline ZIF-UC-6 before melt-quenching, or (ii) prepare the glass first and then perform PSM on the glass surface directly. As PSM has already been reported on crystalline MOFs quite extensively,^19,43^ strategy (i) was attempted first.

### Preparation of Modified *a*_g_ZIF-UC-6—Strategy (i)

The procedure for PSM on crystalline ZIF-UC-6 was based on previous reports on the modification of IRMOF-3.^33,35^ Briefly, ZIF-UC-6 was suspended in a dilute solution of octyl isocyanate in chloroform and left to react for 24 h before a rigorous washing and activation procedure (Methods). The retention of crystallinity after PSM was confirmed by powder X-ray diffraction (PXRD), and no crystalline impurities introduced during the PSM process were found (Figure S23, Table S6).

Fourier-transform infrared (FTIR) spectroscopy was performed on unmodified and modified ZIF-UC-6 (Figure S24). However, no discernible changes in the spectra were observed which is likely due to the low content (9%) of the amine linker. This could also be ascribed to a lack of the amine moiety in the framework; however, the presence of two distinct linker environments by ^1^H NMR spectroscopy (Figure S8) combined with its strong color and absorbance in the visible spectrum (Figures S2, S3, S7) point to the amine linker being present, albeit at low levels.

^1^H NMR spectroscopy was performed on the modified sample (Figure S25) along with imidazole, 5-aminobenzimidazole, and octyl isocyanate for reference (Figures S26–S28). A new two position imidazole singlet peak at 9.53 ppm was observed for modified ZIF-UC-6 that did not correspond to imidazole or 5-aminobenzimidazole and supported the presence of a new imidazole type linker in the structure (Figure S25b). Additionally, multiple new aromatic environments, with matching peak integrations to the new singlet at 9.53 ppm, also supported the formation of a new substituted linker (Figure S25c). Furthermore, clear evidence of the eight-membered carbon chain was also observed below 1.5 ppm, again with peak integrations correlating with the other new environments formed (Figure S25d). The composition of the modified material was then determined by peak integration, giving a formula of [Zn(Im)_1.82_(abIm)_0.16_(PSM linker)_0.02_] that is, an 11% conversion of the available abIm linkers and a 1% conversion of the total organic molecules present within the structure. This conversion was not expected to be discernible by techniques such as CHN microanalysis, as the changes would be within instrumental error. Our experimental observations support this, as no significant changes in the wt % of C, H or N were observed after modification (Tables S7, S8).

To further support the successful formation of a modified linker, a digested sample of modified ZIF-UC-6 was subjected to high resolution electrospray ionization mass spectrometric analysis. The presence of the molecular ion with formula [C_16_H_24_N_4_O]H^+^ (calc: *m*/*z* = 289.2028; found: *m*/*z* = 289.2023; 1.7 ppm) strongly suggests that covalent modification of 5-aminobenzimidazolate to form the octyl-urea adduct has occurred, rather than physisorption of octyl isocyanate onto the surface of the MOF particles (Figures S29, S30).

Finally, to confirm that the reactive amine functionality in ZIF-UC-6 was undergoing the PSM reaction and octyl isocyanate was not simply physisorbed on the surface, a control experiment was performed using ZIF-62, as it contains unfunctionalized benzimidazolate linkers. A sample of ZIF-62 [Zn(Im)_1.8_(bIm)_0.2_] was prepared by previously reported methods^46^ and treated with octyl isocyanate in an identical manner to ZIF-UC-6 before being analyzed by ^1^H NMR spectroscopy (Figure S31). Unlike ZIF-UC-6, there was no evidence for the formation of a new two position imidazole proton environment and there was minimal evidence of the carbon chain of octyl isocyanate left after the attempted reaction. This further supports the success of the modification reaction on ZIF-UC-6 and highlights that the reactive amine group is needed for the PSM reaction to occur.

CO_2_ gas sorption on modified ZIF-UC-6 revealed a maximum CO_2_ uptake of 43.3 cm^3^ g^–1^ at STP (Figure S32, Table S9), that is, an uptake very close to that observed for unmodified ZIF-UC-6 (46.9 cm^3^ g^–1^ at STP). This suggests that PSM does not dramatically alter the porosity of the ZIF due to the low percentage conversion (*ca.* 1%) of the organic molecules within the framework. The modification reaction likely occurs mostly on the particle surface, that is, not affecting the internal pore structure of the ZIF. The slightly lower value for the maximum uptake of CO_2_ in the modified material is attributed to the partial blocking of the pore network by the newly introduced alkyl chains from octyl isocyanate. The modified ZIF also exhibited more pronounced hysteresis compared to the unmodified material. This is also attributed to the newly introduced alkyl chains inhibiting the diffusion of CO_2_ in the framework.

Modified ZIF-UC-6 was then examined using DSC and TGA (Figures S33–35) to detect changes in its thermal behavior caused by PSM. Its thermal stability was identical to the unmodified material (Figure S33). However, a mass loss (9.9%) between 200 and 346 °C (Figure S34) may be attributed to partial decomposition of the modified linkers. The washing and activation procedure used here has been reported to remove most unreacted species.^33,47^ Additionally, neat octyl isocyanate has a boiling point of 200–204 °C, which is lower than the observed mass loss here.^33^ This mass loss was also accompanied by a sharp endo- and exothermic event in the DSC (Figure S35). Desolvation is an endothermic process and occurs at lower temperatures than this event, which had a closer resemblance to thermal amorphization events in other ZIFs.^48,49^ Interestingly, upon further heating, no indication of melting was detected. However, a possible *T*_g_ at 314 °C was observed upon reheating (Figure S35), and subsequent PXRD confirmed the sample to be X-ray amorphous (Figure S36). ^1^H NMR spectroscopy revealed that, after heating, the carbon chains were still present in the structure to some extent, although the emergence of multiple new environments suggested that partial decomposition had occurred (Figure S37).

### Preparation of Modified *a*_g_ZIF-UC-6—Strategy (ii)

As the abovementioned strategy resulted in significant sample decomposition, strategy (ii), that is, direct modification of *a*_g_ZIF-UC-6, was then attempted (Methods, Figure S38). Briefly, *a*_g_ZIF-UC-6 was suspended in a dilute solution of octyl isocyanate in chloroform and left for 24 h before the sample was washed and activated. CHN microanalysis on modified *a*_g_ZIF-UC-6 showed minimal differences when compared to the unmodified sample, and this is attributed again to the small fraction of the structure that undergoes the PSM reaction (Tables S10, S11). ^1^H NMR spectroscopy on the modified glass did not exhibit a peak in the imidazole region that corresponded to the modified linker (Figure S39b). However, close inspection of the satellite peaks of the imidazole proton peak revealed asymmetric integration values, suggesting that the modified linker in the glass could be masked by these peaks. This was accompanied by the presence of new aromatic linker environments of the expected intensity (Figure S39c) and evidence of the carbon chain below 1.5 ppm (Figure S39d). The composition of the modified glass was then determined and found to be identical to the crystalline sample [Zn(Im)_1.82_(abIm)_0.16_(PSM linker)_0.02_], that is, the same as strategy (i), an 11% conversion of the available abIm linkers and a 1% conversion of the total organic molecules present within the structure.

As with the crystalline sample, mass spectrometric analysis of modified *a*_g_ZIF-UC-6 showed the presence of the octyl-urea adduct, with formula [C_16_H_24_N_4_O]H^+^ (calc: *m*/*z* = 289.2028; found: *m*/*z* = 289.2023; 2.0 ppm) again confirming linker modification (Figures S40 and S41).

Modified *a*_g_ZIF-UC-6 displayed a maximum CO_2_ uptake of 21.0 cm^3^ g^–1^ at STP (Figure S42, Table S12). This value was close to that observed for unmodified *a*_g_ZIF-UC-6 (23.0 cm^3^ g^–1^ at STP). As for the crystalline sample, this indicates that the PSM procedure does not dramatically alter the porosity of the ZIF glass. The small difference in the uptake of CO_2_ in the modified glass is again attributed to the presence of alkyl chains in the structure. The difference in maximum uptake between modified *a*_g_ZIF-UC-6 and modified ZIF-UC-6 is similar to the difference between their unmodified counterparts (Figure S43), suggesting that the maximum CO_2_ uptake is influenced more by the framework structure, that is, crystal or glass, than by the PSM process.

The thermal response of modified *a*_g_ZIF-UC-6 was investigated (Figure S44–S46) to identify changes in its thermal behavior caused by PSM. The thermal stability of the glass was largely unaffected by modification (Figures S44 and S45). However, a distinct change in the DSC of modified *a*_g_ZIF-UC-6 was observed (Figure S46), with a broad *T*_g_ observed at 290 °C in the first upscan. This is different to the behavior of modified crystalline ZIF-UC-6 which exhibited a sharp endo- and exothermic event in the first upscan of the DSC. Upon re-heating-modified *a*_g_ZIF-UC-6, a *T*_g_ was observed (319 °C), consistent with unmodified *a*_g_ZIF-UC-6. The behavior in the first upscan could be attributed to the partial decomposition of the modified linkers with heating. After thermal decomposition of these linkers, the bulk composition of the modified glass is likely to be very similar to the composition of the unmodified glass, resulting in a *T*_g_ more consistent with this material.

**Figure 5:**
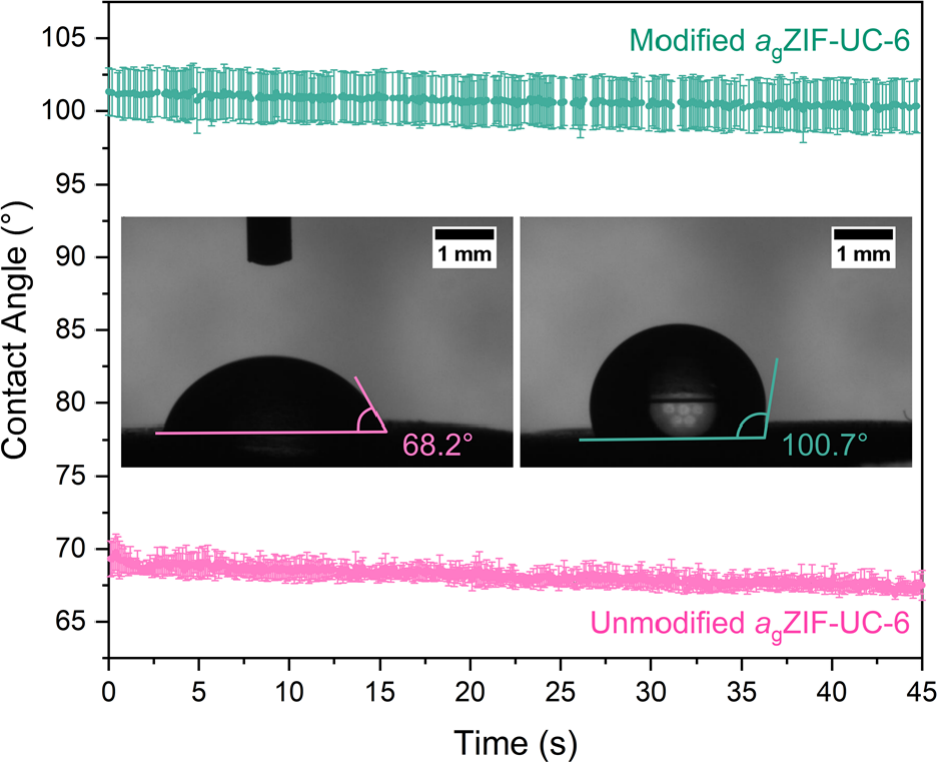
Water contact angle data collected on unmodified *a*_g_ZIF-UC-6 (pink) and modified *a*_g_ZIF-UC-6 (green) collected over 45 s. Insets: microscope images of water on the unmodified (left) and modified glass (right) surfaces showing overall mean contact angles of 68.2 ± 0.6 and 100.7 ± 2.2° respectively. Lines and angles drawn on these images are a guide only.

To demonstrate the ability of PSM to tune the bulk properties of the ZIF glass surface, two *a*_g_ZIF-UC-6 pellets were prepared. One was left unmodified, and the other was modified with octyl isocyanate in a manner similar to the glass powder described above (Methods). The hydrophobicity was then probed using water contact angle measurements (Figures 5, S47). These measurements can only be performed on flat surfaces, making them ideal for studying the ZIF glass surface. Hence, an equivalent measurement of the modified crystalline ZIF-UC-6 powder was not possible. The water contact angle measurements revealed that the unmodified glass surface was relatively hydrophilic with a mean water contact angle of 68.2 ± 0.6°. However, modification of the glass surface with carbon chains from octyl isocyanate caused a dramatic increase in the hydrophobicity of the glass surface, resulting in a mean water contact angle of 100.7 ± 2.2° (Figures 5, S47). This clearly demonstrates the success of the modification reaction and highlights the promise of this method for tuning the properties of the ZIF glass surface.

To further demonstrate the scope of PSM for tuning the hydrophobicity of ZIF glasses, a third a_g_ZIF-UC-6 pellet was prepared before using an identical modification procedure, albeit this time with dodecyl isocyanate as the modification reagent (Methods). Hydrophobicity measurements revealed a water contact angle of 114.7 ± 2.7° (Figure S48), which is significantly higher than that observed after modification with octyl isocyanate. This implies that increasing the carbon chain length results in an increase in hydrophobicity at the glass surface, although further experiments would be needed to confirm the veracity of this conclusion. Ultimately, it is hoped that the precise hydrophobicity of the ZIF glass surface could be controlled by judicious choice of the carbon chain length used during modification.

## Conclusions

In this work, we have successfully prepared a novel liquid and glass-forming ZIF, denoted ZIF-UC-6. Crystallizing in the *Pbca* space group and exhibiting the **cag** topology, with composition [Zn(Im)_2–*x*_(abIm)_*x*_] (where *x* = 0.18), the framework displays strong structural similarities with other glass-forming ZIFs.^14^ The amine functionality introduced into the framework by the abIm linker causes a lowering of both *T*_m_ (345 °C) and *T*_g_ (316 °C) compared to other **cag** topology ZIFs. Additionally, this amine functionality provided a promising target for PSM, thereby facilitating the modification of the ZIF in its crystalline form as well as the glass form for the first time.

As a prototypical example, we investigated the effect on the wetting ability of the glass surface by its reaction with octyl isocyanate. A 32.5° increase in the water contact angle after PSM supports the success of the reaction. Moreover, modification of the ZIF glass surface with dodecyl isocyanate resulted in an increase in the water contact angle by 46.5° compared to the unmodified glass, implying that careful consideration of the carbon chain length can be used to precisely control the wetting behavior of the ZIF glass surface. These results confirm PSM as a promising strategy for tuning the hydrophobicity of the glass surface, almost independently of porosity. This enhancement in hydrophobicity could be utilized to facilitate the preparation of hydrophobic ZIF glass coatings, for example. However, PSM of ZIF glasses is not limited to the reaction demonstrated here and, in fact, the library of PSM reactions that have been demonstrated in crystalline systems can now potentially be applied to glasses as well. Some potential avenues for future research could include surface modification to achieve catalytically active ZIF glasses, or the incorporation of photoresponsive functional groups to prepare light-responsive ZIF glasses.^50^ Further studies to expand PSM to other ZIF glasses, as well as incorporating additional chemical functionality at the glass surface are now underway.

## Methods

### Materials

Imidazole (≥99.5%), D_2_O (35 wt.% DCl), octyl isocyanate (97%), dodecyl isocyanate (99%), and chloroform (99.0–99.4% GC) were purchased from Sigma-Aldrich. Dimethyl sulfoxide (DMSO)-*d*_6_ [99.8 atom % D, contains 0.03% (v/v) tetramethylsilane (TMS)] was purchased from VWR. Zinc nitrate hexahydrate (98%) was purchased from Alfa Aesar. *N*,*N*-Dimethylformamide (DMF) (99.5%), chloroform (reagent grade), and dichloromethane stabilized with amylene (DCM) (99.8%) were purchased from Fischer Scientific. 5-Aminobenzimidazole (>99%) was purchased from Santa Cruz Biotechnology. All materials were used without further purification.

### Single Crystal Synthesis of ZIF-UC-6

Zinc nitrate hexahydrate (0.095 g, 0.32 mmol), imidazole (0.460 g, 6.76 mmol), and 5-aminobenzimidazole (0.100 g, 0.75 mmol) were dissolved in DMF (4.7 mL) to give a dark red-colored solution. This solution was heated to 130 °C and held there for 48 h before being cooled to 30 °C at 5 °C h^–1^. The resulting burgundy crystals were collected by vacuum filtration, washed with fresh DMF, and stored in fresh DMF until needed.

### Bulk Solvothermal Synthesis of ZIF-UC-6

Zinc nitrate hexahydrate (1.515 g, 5.09 mmol), imidazole (7.350 g, 108 mmol), and 5-aminobenzimidazole (1.598 g, 12.0 mmol) were dissolved in DMF (75 mL) to give a dark red-colored solution. This solution was heated to 130 °C and held there for 48 h in an oven before being removed and left to cool to room temperature naturally. The resulting burgundy polycrystalline powder was isolated by vacuum filtration and washed with fresh DMF. The sample was then soaked in DCM (5 mL) for 24 h in a sealed vial to allow solvent exchange to take place. The powder was again isolated by vacuum filtration before being activated under vacuum at 170 °C for 3 h. This synthetic method was designed based on the reported synthesis of ZIF-UC-5.^14^

### PSM of ZIF-UC-6 and *a*_g_ZIF-UC-6 Powder

ZIF-UC-6 and *a*_g_ZIF-UC-6 (42 mg, 0.20 mmol, 0.04 mmol equivalents of −NH_2_) were suspended in chloroform (2 mL). Octyl isocyanate (56 μL, 0.32 mmol, 8 equivalents compared to −NH_2_) was added, and the suspension was shaken to disperse the reagents. The suspension was left to stand for 24 h. The powder was then collected by vacuum filtration and washed with chloroform (3 × 6 mL) before being soaked in fresh chloroform (3 mL) for a minimum of 3 days. The powder was again isolated by vacuum filtration before being activated under vacuum at 100 °C overnight (*ca.* 18 h). This synthetic method was based on the PSM methods previously reported by Cohen *et al.* on IRMOF-3.^33,35^ The exact same modification and washing procedure was performed for the control experiment using ZIF-62 (42 mg), where ZIF-62 was prepared using a previously reported method.^46^

### Pellet Preparation of *a*_g_ZIF-UC-6

Crystalline ZIF-UC-6 (*ca.* 150 mg) was placed inside a 13 mm pellet die and compressed at a pressure of *ca.* 0.15 GPa for 1 min before the pressure was released. This gave a smooth pellet of crystalline ZIF-UC-6. This pellet was then melted by heating to 409 °C in a Carbolite tube furnace under a flowing argon atmosphere. After waiting for 1 min at 409 °C, the pelletized glass sample was cooled to 200 °C under flowing argon. The argon stream was then turned off, and the sample was allowed to cool to room temperature. The loss of any defined Bragg reflections was then confirmed by PXRD.

### PSM of *a*_g_ZIF-UC-6 Pellet—Octyl Isocyanate

A 13 mm *a*_g_ZIF-UC-6 pellet (66 mg, 0.31 mmol, 0.06 mmol equivalents of −NH_2_) was suspended in chloroform (2 mL). Octyl isocyanate (85 μL, 0.48 mmol, 8 equivalents compared to −NH_2_) was added, and the suspension was left to stand for one day. The pellet was then collected by vacuum filtration and washed with chloroform (3 × 6 mL) before being soaked in fresh chloroform (3 mL) for 6 days. The pellet was again isolated by vacuum filtration before being activated under vacuum at 100 °C overnight (*ca.* 18 h). This was based on the PSM methods previously reported on IRMOF-3.^33,35^

### PSM of *a*_g_ZIF-UC-6 Pellet—Dodecyl Isocyanate

A 13 mm *a*_g_ZIF-UC-6 pellet (73 mg, 0.34 mmol, 0.07 mmol equivalents of −NH_2_) was suspended in chloroform (2 mL). Dodecyl isocyanate (134 μL, 0.56 mmol, 8 equivalents compared to −NH_2_) was added, and the suspension was left to stand for one day. The pellet was then collected by vacuum filtration and washed according to the procedure described above.

### Single-Crystal X-Ray Diffraction

Single-crystal diffraction data were collected using a Bruker D8 VENTURE diffractometer equipped with a Bruker PHOTON II detector at 150 K using graphite monochromated Mo Kα radiation (λ = 0.71073). Data reduction was done using the APEX3 program. Absorption correction based on multiscan was obtained by SADABS. The structure was solved and refined using SHELXT and SHELXL packages correspondingly, using the OLEX2 program.^51−53^ The electron density within the voids was accounted for using the SQUEEZE program implemented in PLATON.^54^

Crystal data for ZIF-UC-6: C_13.59_H_12.78_N_8.39_Zn_2_, *M*_r_ = 424.36, crystal dimensions 0.195 × 0.156 × 0.077 mm, Orthorhombic, *a* = 15.839(2) Å, *b* = 15.599(2) Å, *c* = 17.984(2) Å, α = β = γ = 90°, *V* = 4443.3(9) Å^3^, space group, *Pbca*, (no. 61), *T* = 150 K, *Z* = 8, 24514 measured reflections, 4524 independent reflections (*R*_int_ = 0.0593), which were used in all calculations. The final *R*_1_ = 0.053 for 3285 observed data *R*[*F*^2^ > 2σ(*F*^2^)] and *wR*(*F*^2^) = 0.139 (all data). CSD deposition 2115059.

### Powder X-Ray Diffraction

Data were collected on a Bruker D8 ADVANCE diffractometer equipped with a position-sensitive LynxEye detector with Bragg–Brentano parafocusing geometry. Cu Kα (λ = 1.5418 Å) radiation was used. The samples were compacted into 5 mm disks on a low background silicon substrate and rotated during data collection in the 2θ range of 5–40° at ambient temperature. All data conversion from .raw files to .xy files was performed using PowDLL.^55^ Pawley refinements were performed using TOPAS-Academic Version 6.^56^ Thompson–Cox–Hastings pseudo-Voigt peaks shapes were used along with a simple axial divergence correction. The lattice parameters were refined in the 2θ range of 5–40° against the values obtained from the CIF for ZIF-UC-6 reported in this work. The zero-point error was also refined.

### Differential Scanning Calorimetry

Data were collected on a Netzsch DSC 214 Polyma Instrument. Heating and cooling rates of 10 °C min^–1^ were used in conjunction with a flowing argon atmosphere. Sealed aluminum pans (30 μL) were used with a hole punctured in the lid to prevent pressure build-up. An empty aluminum pan was used as a reference. Background corrections were performed using the same heating cycle on an empty aluminum crucible. All data analysis was performed using the Netzsch *Proteus* software package. *T*_m_ was taken as the offset (the end point) of the melting endotherm. *T*_g_ was taken as the mid-point of the change in gradient of the heat flow of the DSC on the second upscan.

### Thermogravimetric Analysis

Data were collected on a TA Instruments SDT-Q600 using alumina pans (90 μL). Heating and cooling rates of 10 °C min^–1^ were used, and experiments were conducted under a flowing argon atmosphere. All data analyses were performed using the TA Instruments Universal Analysis software package. The temperature used to define any weight changes was determined using the first derivative of the weight (%) trace as a function of temperature.

### ^1^H Nuclear Magnetic Resonance Spectroscopy

^1^H NMR spectra were recorded at 298 K using a Bruker AVIII 500 MHz Spectrometer with a dual ^13^C/^1^H (DCH) cryoprobe. Samples of crystalline ZIF-UC-6 and *a*_g_ZIF-UC-6 were dissolved in a mixture of DCl (35%)/D_2_O and DMSO-*d*_6_ in a 1:5 ratio with TMS used as a reference. For samples of ZIF-UC-6 and *a*_g_ZIF-UC-6 which had undergone PSM, as well as unreacted imidazole and 5-aminobenzimidazole, *ca.* 5.5 mg of sample was dissolved in DMSO-*d*_6_ (1.5 mL) and a dilute solution of DCl (200 μL) with TMS again used as a reference. This dilute solution was prepared from DCl (35%)/D_2_O (23 μL) and DMSO-*d*_6_ (1 mL). Dilute conditions were used to prevent acid-catalyzed hydrolysis of the newly formed urea functionalized linkers. These conditions were based on those used by Cohen *et al.* in their studies on the PSM of IRMOF-3.^33 1^H NMR spectra were also collected on octyl isocyanate (dissolved in DMSO-*d*_6_ only to prevent any reaction with DCl/D_2_O). The sample of ZIF-62 used as a PSM control was dissolved in a mixture of DCl (35%)/D_2_O and DMSO-*d*_6_ in a 1:5 ratio with TMS as a reference. All data processing was performed using the Bruker TopSpin 4.0.7 software package.

### Mass Spectrometry

High resolution electrospray ionization mass spectra were collected on an Agilent 6546 QTOF-MS in positive ion mode using direct infusion. Samples of modified ZIF-UC-6 and *a*_g_ZIF-UC-6 were suspended in an aqueous Na_2_EDTA solution (0.6 M), HCl added dropwise until solids had dissolved, and extracted with CHCl_3_. The organic layer was then dried over Na_2_SO_4_ and evaporated before dissolution in methanol prior to injection and run in 70:30 *v*/*v* MeCN:H_2_O with 1% formic acid. This methodology is adapted from previously reported protocols for analysis of MOF surface modification.^57^

### CO_2_ Gas Sorption

Measurements were performed on a Micromeritics ASAP 2020 surface area and porosity analyzer. Samples of ZIF-UC-6 (*ca.* 140 mg), *a*_g_ZIF-UC-6 (*ca.* 110 mg), modified ZIF-UC-6 (*ca.* 60 mg), and modified *a*_g_ZIF-UC-6 (*ca.* 90 mg) were degassed by heating under vacuum at 100 °C for 12 h before analysis using carbon dioxide gas at 273 K. Gas uptake was determined using the Micromeritics MicroActive software package.

### Diffuse-Reflectance UV–Vis Spectroscopy

Measurements were carried out using a PerkinElmer Lambda 750 spectrophotometer equipped with a Labsphere 60 mm RSA ASSY integrating sphere. Samples were measured in a powder sample holder with a fused quartz disc. Spectra were then transformed using the Kubelka–Munk transformation.^58,59^

### Fourier-Transform Infrared Spectroscopy

IR spectra were collected on powder samples using a Bruker Tensor 27 FTIR spectrometer in transmission mode between 550 and 4000 cm^–1^. A background was subtracted from all spectra prior to analysis.

### Scanning Electron Microscopy

Scanning electron microscopy (SEM) images were collected with a high-resolution scanning electron microscope FEI Nova Nano SEM 450, with an accelerating voltage of 15 kV. All samples were prepared by dispersing the material onto double sided adhesive conductive carbon tape that was attached to a flat aluminum sample holder and coated with a platinum layer of 15 nm.

### CHN Microanalysis

CHN combustion analysis experiments were performed using a CE440 Elemental Analyzer, EAI Exeter Analytical Inc. ∼1.3–1.5 mg of the sample was used for each run. Measurements were collected up to 3 times per sample.

### X-Ray Total Scattering—PDF

X-ray total scattering data were collected at beamline I15-1, Diamond Light Source, UK (EE20038) on crystalline ZIF-UC-6 and glass *a*_g_ZIF-UC-6. A bulk sample of *a*_g_ZIF-UC-6 was prepared by heating crystalline ZIF-UC-6 (100 mg) to 400 °C (and holding it there for 1 min before cooling) in a Carbolite tube furnace under a flowing argon atmosphere. Both samples were ground and loaded into borosilicate glass capillaries (1.17 mm inner diameter) to heights of 3.7 cm (ZIF-UC-6) and 3.8 cm (*a*_g_ZIF-UC-6). The capillaries were then sealed before being mounted onto the beamline. Total scattering data were collected at room temperature for the background (*i.e.*, empty instrument), empty borosilicate capillary, and for both samples in a *Q* range of 0.4–26.0 Å^–1^ (λ = 0.161669 Å, 76.69 keV). Data for *a*_g_ZIF-UC-6 were collected at 100% flux. Data for crystalline ZIF-UC-6 were collected at 10% flux to prevent saturation of the detector due to the high crystallinity of this sample. The total scattering data were processed to account for the difference in beam flux incident on each sample, along with absorption corrections and various scattering corrections—background scattering, multiple scattering, container scattering, and Compton scattering—in a *Q* range of 0.55–26.0 Å^–1^. The crystallographic density of ZIF-UC-6 was taken as the density for both ZIF-UC-6 and *a*_g_ZIF-UC-6 during the data processing. Subsequent Fourier transformation of the processed total scattering data resulted in a real space PDF *G*(*r*) for each material. In this work, we use the *D*(*r*) form of the PDF to accentuate high *r* correlations. All processing of the total scattering data was performed using GudrunX following well documented procedures.^60−62^

### Contact Angle Measurements

An FTA1000 B class instrument was used to acquire contact angles between water and the surface of modified and unmodified *a*_g_ZIF-UC-6 pellets (13 mm diameter). Static contact angles of a droplet of water (*ca.* 10 μL) were measured over a period of 45 s, and an average of at least three droplets was taken for each sample. All analyses were performed using the FTA32 software package.^63^ The overall mean contact angle for each surface was then determined.

## Supporting Information Available

Detailed experimental methods and techniques, synthesis and characterization of ZIF-UC-6, *a*_g_ZIF-UC-6 and modified samples including optical microscopy, SCXRD, PXRD, diffuse-reflectance UV–vis spectroscopy, CO_2_ gas sorption, ^1^H NMR spectroscopy, TGA, DSC, SEM, X-ray total scattering, FTIR, CHN microanalysis, and water contact angle measurements (PDF)
ZIF-UC-6 Crystal Structure (CIF)

## Supplementary Material

cm1c03820_si_001.pdf

cm1c03820_si_002.cif
