# Supplementary material for: Post-Synthetic Modification of a Metal–Organic Framework Glass
Source: Chem Mater. 2022 Feb 18;34(5):2187–96. doi: 10.1021/acs.chemmater.1c03820 (PMC9100367; doi:10.1021/acs.chemmater.1c03820)
Supplement: Supplementary file 1 — cm1c03820_si_001.pdf [file cm1c03820_si_001.pdf]

Supporting Information

Post-Synthetic Modification of a Metal–Organic Framework Glass

Alice M. Bumstead[†], Ignas Pakamorė[‡], Kieran D. Richards[†], Michael F. Thorne[†], Sophia S. Boyadjieva[‡], Celia Castillo-Blas[†], Lauren N. McHugh[†], Adam F. Sapnik[†], Dean S. Keeble[§], David A. Keen[∥], Rachel C. Evans[†], Ross S. Forgan[‡], and Thomas D. Bennett[†*]

[†] Department of Materials Science and Metallurgy, University of Cambridge, Cambridge, CB3 0FS, UK.

[‡] WestCHEM, School of Chemistry, The University of Glasgow, University Avenue, Glasgow, G12 8QQ, UK.

[§] Diamond Light Source Ltd, Diamond House, Harwell Campus, Didcot, Oxfordshire, OX11 0DE, UK.

[∥] ISIS Facility, Rutherford Appleton Laboratory, Harwell Campus, Didcot, Oxfordshire, OX11 0QX, UK.

* Email: tdb35@cam.ac.

Contents List

Figures

Figure S1: Reaction scheme for the formation of ZIF-UC-6.

Figure S2: Images before, during and after synthesis of ZIF-UC-6.

Figure S3: Single crystals of ZIF-UC-6.

Figure S4: Crystal structure of ZIF-UC-6.

Figure S5: Powder X-ray diffraction patterns of ZIF-UC-6 and a_gZIF-UC-6.

Figure S6: Pawley refinement of powder X-ray diffraction data of ZIF-UC-6.

Figure S7: Diffuse-reflectance UV-Vis spectroscopy (DRUV) performed on ZIF-UC-6.

Figure S8: ^{1}H nuclear magnetic resonance spectrum of ZIF-UC-6.

Figure S9: Thermogravimetric analysis of crystalline ZIF-UC-6 to 1000 °C.

Figure S10: Thermogravimetric analysis of crystalline ZIF-UC-6 to 550 °C.

Figure S11: Differential scanning calorimetry of crystalline ZIF-UC-6.

Figure S12: Enthalpy and entropy of fusion of ZIF-UC-6.

Figure S13: Scanning electron microscope images of crystalline ZIF-UC-6 and a_gZIF-UC-6.

Figure S14: ^{1}H nuclear magnetic resonance spectrum of a_gZIF-UC-6.

Figure S15: Thermogravimetric analysis of a_gZIF-UC-6 to 1000 °C.

Figure S16: Thermogravimetric analysis of a_gZIF-UC-6 to 550 °C.

Figure S17: CO_2 adsorption and desorption isotherms collected on crystalline ZIF-UC-6.

Figure S18: CO_2 adsorption and desorption isotherms collected on a_gZIF-UC-6.

Figure S19: Total scattering structure factors of ZIF-UC-6 and a_gZIF-UC-6.

Figure S20: Reaction scheme of amine functionalized MOFs undergoing PSM with isocyanates.

Figure S21: Schematic of the surface modification of a_gZIF-UC-6.

Figure S22: Pore diameter of ZIF-UC-6 and size of octyl isocyanate molecule.

Figure S23: Pawley refinement of ZIF-UC-6 after modification with octyl isocyanate.

Figure S24: FTIR spectra of ZIF-UC-6 and modified ZIF-UC-6.

Figure S25: ^{1}H nuclear magnetic resonance spectrum of ZIF-UC-6 PSM.

Figure S26: ^{1}H nuclear magnetic resonance spectrum of imidazole.

Figure S27: ^{1}H nuclear magnetic resonance spectrum of 5-aminobenzimidazole.

Figure S28: ^{1}H nuclear magnetic resonance spectrum of octyl isocyanate.

Figure S29: Mass spectrum of digested modified crystalline ZIF-UC-6 powder.

Figure S30: Portion of the mass spectrum focused on the molecular ion peak for the modified linker.

Figure S31: ^{1}H nuclear magnetic resonance spectrum of ZIF-62 control experiment.

Figure S32: CO_2 adsorption and desorption isotherms collected on modified crystalline ZIF-UC-6.

Figure S33: Thermogravimetric analysis of crystalline ZIF-UC-6 PSM to 1000 °C.

Figure S34: Thermogravimetric analysis of crystalline ZIF-UC-6 PSM to 550 °C.

Figure S35: Differential scanning calorimetry of ZIF-UC-6 PSM.

Figure S36: Powder X-ray diffraction of ZIF-UC-6, ZIF-UC-6 PSM and ZIF-UC-6 PSM post 450 °C.

Figure S37: ^{1}H nuclear magnetic resonance spectrum of ZIF-UC-6 PSM post 450 °C.

Figure S38: Powder X-ray diffraction of ZIF-UC-6, a_gZIF-UC-6, and modified a_gZIF-UC-6.

Figure S39: ^{1}H nuclear magnetic resonance spectrum of a_gZIF-UC-6 PSM.

Figure S40: Mass spectrum of digested modified a_gZIF-UC-6 glass.

Figure S41: Portion of the mass spectrum focused on the molecular ion peak for the modified linker.

Figure S42: CO_2 adsorption and desorption isotherms collected on modified a_gZIF-UC-6.

Figure S43: CO_2 gas sorption isotherms for modified ZIF-UC-6 and modified a_gZIF-UC-6.

Figure S44: Thermogravimetric analysis of modified a_gZIF-UC-6 to 1000 °C.

Figure S45: Thermogravimetric analysis of modified a_gZIF-UC-6 to 550 °C.

Figure S46: Differential scanning calorimetry of modified a_gZIF-UC-6.

Figure S47: Water contact angle measurements on a_gZIF-UC-6 and a_gZIF-UC-6 PSM.

Figure S48: Water contact angle data collected on modified a_gZIF-UC-6 with dodecyl isocyanate.

Tables

Table S1: Crystal data and structure refinement for ZIF-UC-6.

Table S2: Data from Pawley refinement of ZIF-UC-6.

Table S3: Melting thermodynamics of ZIF-UC-6 vs. reported values for ZIF-UC-5 and TIF-4.

Table S4: CO_2 gas sorption results for crystalline ZIF-UC-6.

Table S5: CO_2 gas sorption results for a_gZIF-UC-6.

Table S6: Data from Pawley refinement of modified ZIF-UC-6.

Table S7: CHN microanalysis results for crystalline ZIF-UC-6.

Table S8: CHN microanalysis results for modified crystalline ZIF-UC-6.

Table S9: CO_2 gas sorption results for modified crystalline ZIF-UC-6.

Table S10: CHN microanalysis results for a_gZIF-UC-6.

Table S11: CHN microanalysis results for modified a_gZIF-UC-6.

Table S12: CO_2 gas sorption results for modified a_gZIF-UC-6.

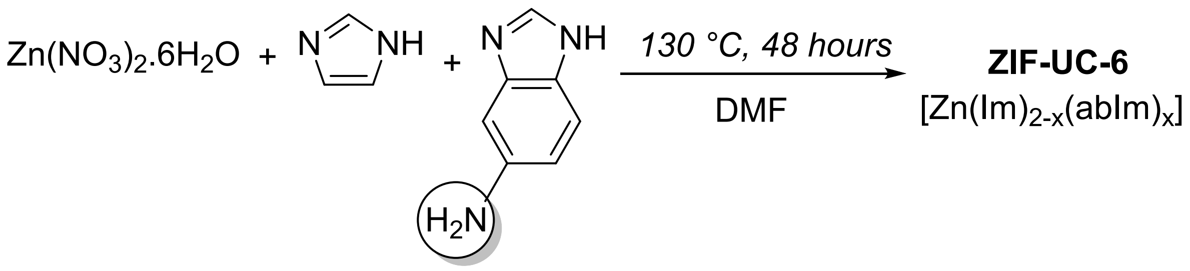

Figure S1: Reaction scheme for the formation of ZIF-UC-6 from zinc nitrate hexahydrate, imidazole and 5-aminobenzimidazole under solvothermal conditions.

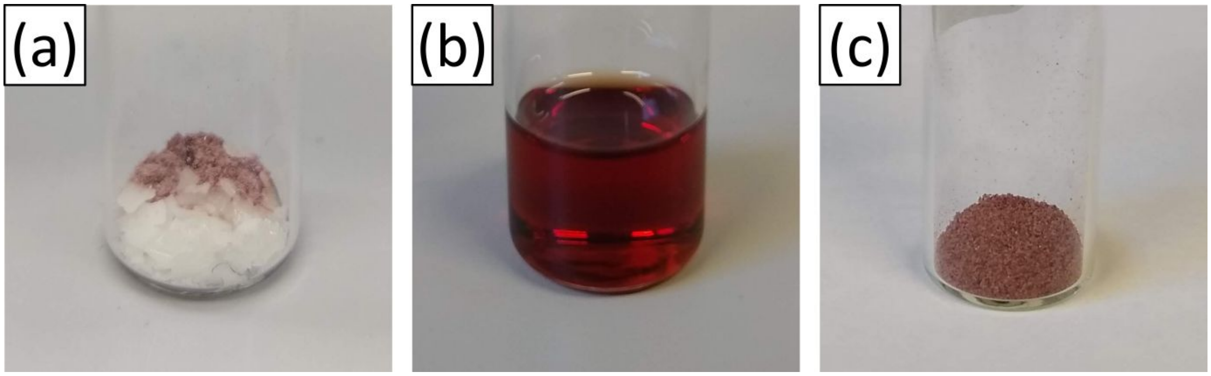

Figure S2: (a) Mixture of zinc nitrate hexahydrate (colorless crystals), imidazole (white flakes) and 5-aminobenzimidazole (dark pink powder) prior to addition of *N,N*-dimethylformamide (DMF); (b) after addition of DMF, all components dissolve to give a red solution; (c) as synthesized ZIF-UC-6 polycrystalline powder (burgundy) after collection by vacuum filtration, solvent exchange with dichloromethane (DCM) and activation at 170 °C for 3 hours under vacuum.

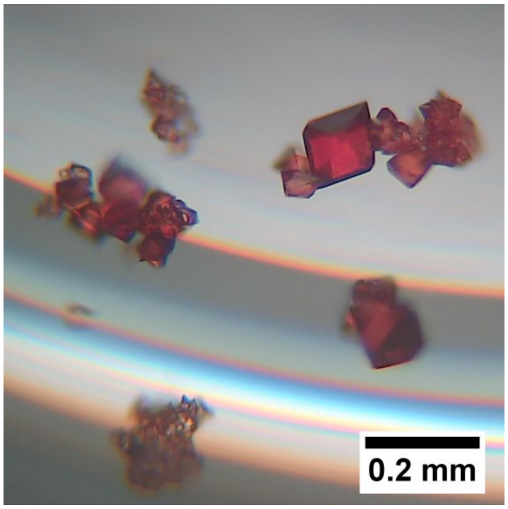

Figure S3: Single crystals of ZIF-UC-6 prepared by cooling the reaction solution at a rate of 5 °C hr^{-1}. Scale bar added using ImageJ 1.52.[1]

Table S1: *Crystal data and structure refinement for ZIF-UC-6.*

Chemical formula	$C_{13.59}$ $H_{12.78}$ $N_{8.39}$ Zn_2
Formula weight	424.36 g mol^{-1}
Temperature	150 K
Wavelength	0.71073 Å
Crystal system	Orthorhombic
Space group	*Pbca*
Unit cell dimensions	a = 15.839(2) A˚ α = 90°
	b = 15.599(2) A˚ β = 90°
	c = 17.984(2) A˚ γ = 90°
Volume	4443.3(9) $Å^3$
Density (calculated)	1.269 g cm^{-3}
Absorption coefficient	2.169
F(000)	1704
Crystal size	0.195 x 0.156 x 0.077 mm^3
Theta range for data collection	2.57– 26.34°
Index ranges	-19 <= h <= 19, -18 <= k <= 19, -22 <= l <= 22
Reflections collected	24514
Independent reflections	4524
Absorption correction	multi-scan
Final R indices [$R[F^2 > 2\sigma(F^2)]$]	R_1 = 0.053 (3285 observed data)
Final R indices (all data)	$wR(F^2)$ = 0.139

(a)

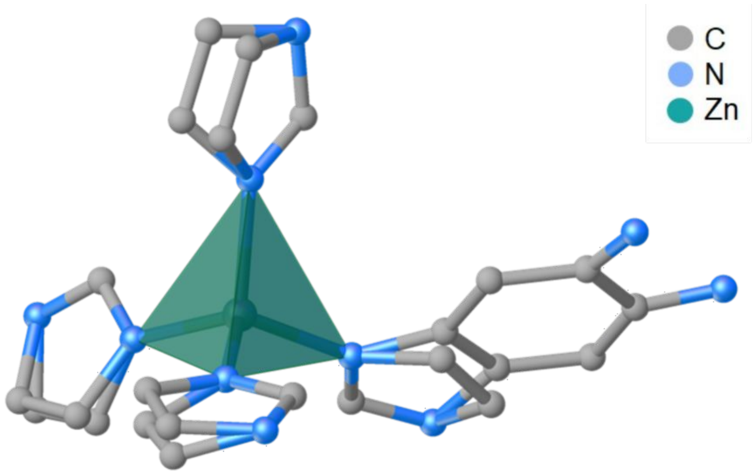

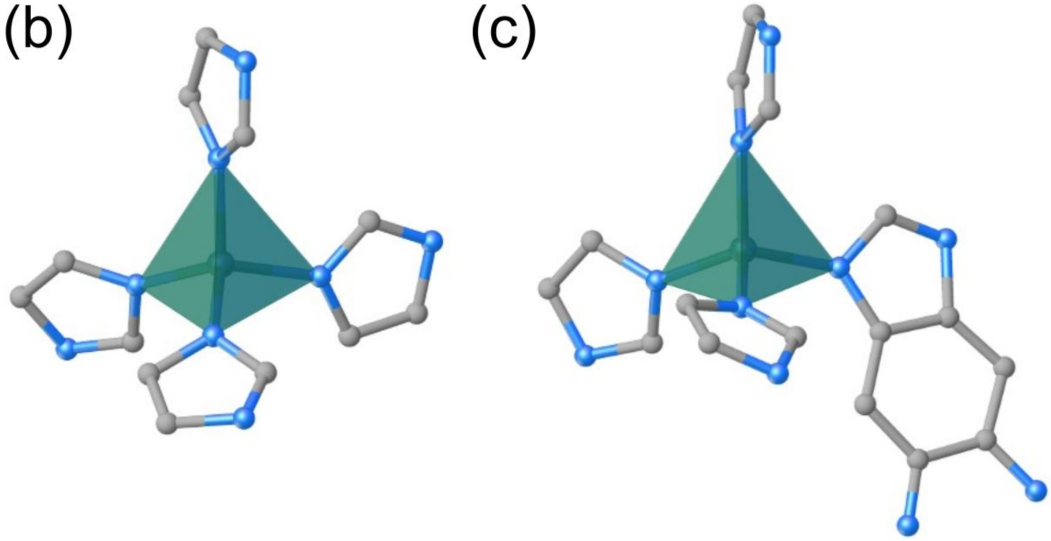

Figure S4: Crystallographic analysis reveals that ZIF-UC-6 crystallizes in the *Pbca* space group with an asymmetric unit (a) consisting of two crystallographically independent Zn^{2+} ions (b, c). Each tetrahedral Zn^{2+} metal centre contains three coordination sites occupied by imidazolate (0.4:0.6 occupancy at two possible orientations) and one by both imidazolate and 5-aminobenzimidazolate (0.4:0.6 occupancy) linkers. Furthermore, the 5-aminobenzimidazolate ligand has one amine group in two possible orientations with occupancy of 0.2 each. These two orientations are visualized by nitrogen being at both the 5- and 6-positions on the ligand. Atoms shown are carbon – grey, nitrogen – blue and zinc – green. All hydrogen atoms have been omitted for clarity.

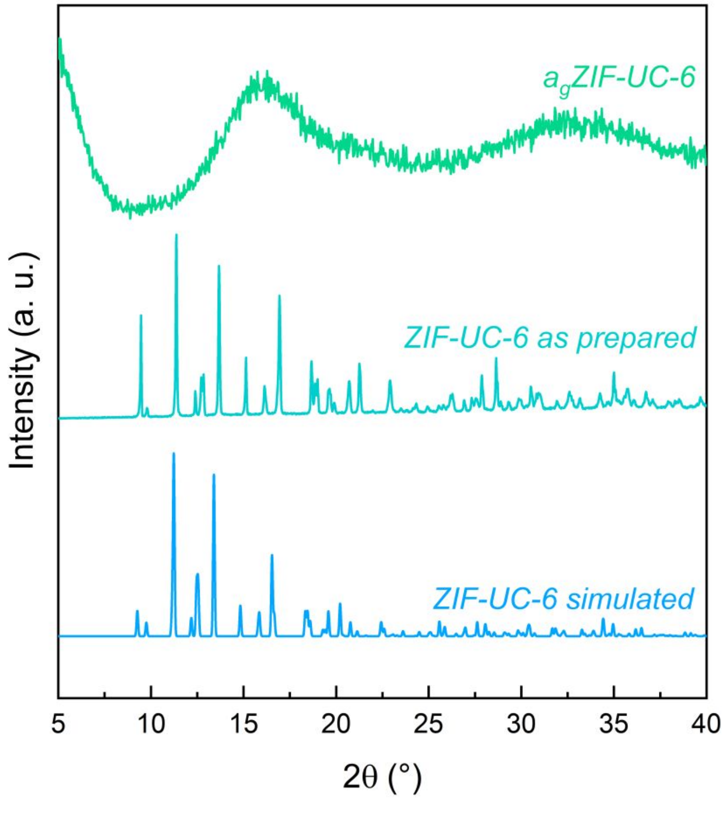

Figure S5: Powder X-ray diffraction patterns between 5-40° of as prepared ZIF-UC-6 (cyan) and a_gZIF-UC-6 (green) (after heating to 400 °C) compared to the simulated diffraction pattern of ZIF-UC-6 (light blue) obtained from the CIF reported in this work from single crystal X-ray diffraction. Conversion of .raw files to .xy files was performed using PowDLL.[2]

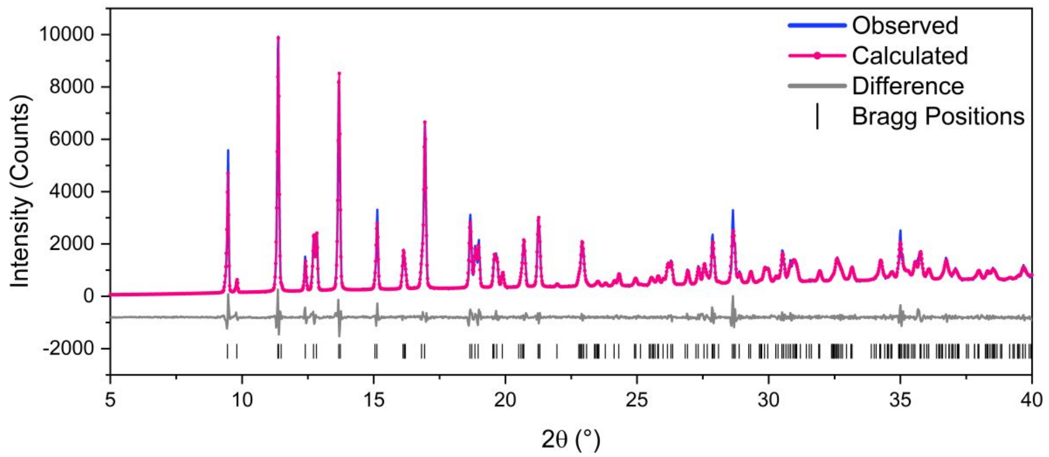

Figure S6: Pawley refinement of powder X-ray diffraction data of ZIF-UC-6. Initial parameters were obtained from the CIF for ZIF-UC-6 reported in this work. Conversion of .raw files to .xy files was performed using PowDLL and all powder refinements were performed using TOPAS-Academic Version 6.[2,3] The difference curve and Bragg positions are offset on the y axis for clarity.

Table S2: Data from Pawley refinement of ZIF-UC-6

R_{wp}	Space Group	Refined Powder Lattice Parameters	Single Crystal Lattice Parameters
7.634	*Pbca*	*a* = 15.3987(12) Å	*a* = 15.839(2) Å
		b = 15.5293(12) Å	*b* = 15.599(2) Å
		c = 18.0366(15) Å	*c* = 17.984(2) Å
		α = β = γ = 90 °	α = β = γ = 90 °

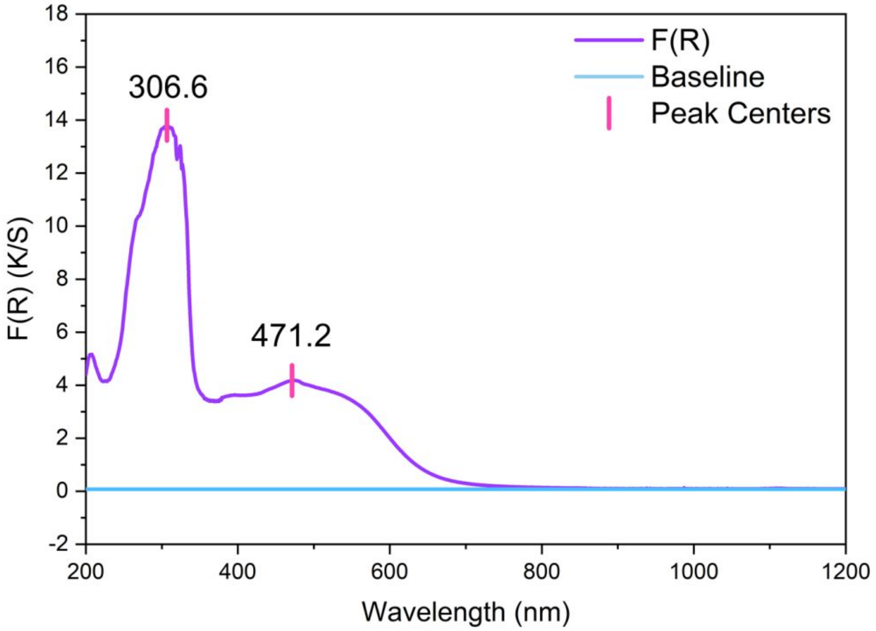

Figure S7: Diffuse-reflectance UV-Vis spectroscopy (DRUV) on ZIF-UC-6 showing two absorbances at 307 nm (UV region) and 471 nm (visible region). The raw reflectance data were converted using the Kubelka-Munk Equation (F(R) = K/S) where F(R) is taken to be equivalent to the absorbance.[4,5] K = $(1\text{-}R)^2$ and S = 2R where R is the absolute reflectance of the material. As 5-aminobenzimidazole was also dark pink in color (**Fig. S2**), the color in ZIF-UC-6 is ascribed to the transition at 471 nm.

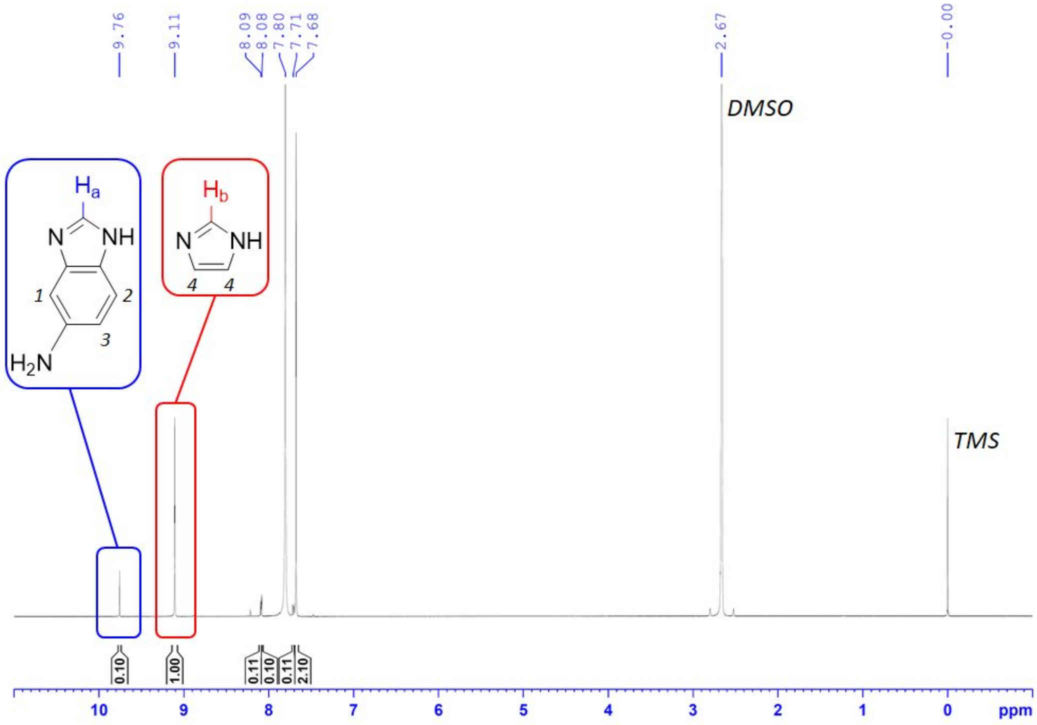

Figure S8: ^{1}H nuclear magnetic resonance spectrum of ZIF-UC-6. All analysis was performed using Topspin Version 4.0.7.[6] δH (500 MHz; DCl(35%)/D_2O:DMSO-d_6 (1:5); Me_4Si) 9.76 (1H, s, H_a), 9.11 (1H, s, H_b), 8.09 (1H, d, H_2), 8.08 (1H, s, H_1), 7.80 (H_2O/HCl), 7.71 (1H, d, H_3), 7.68 (2H, s, H_4) 2.67 (DMSO), 0.00 (TMS).

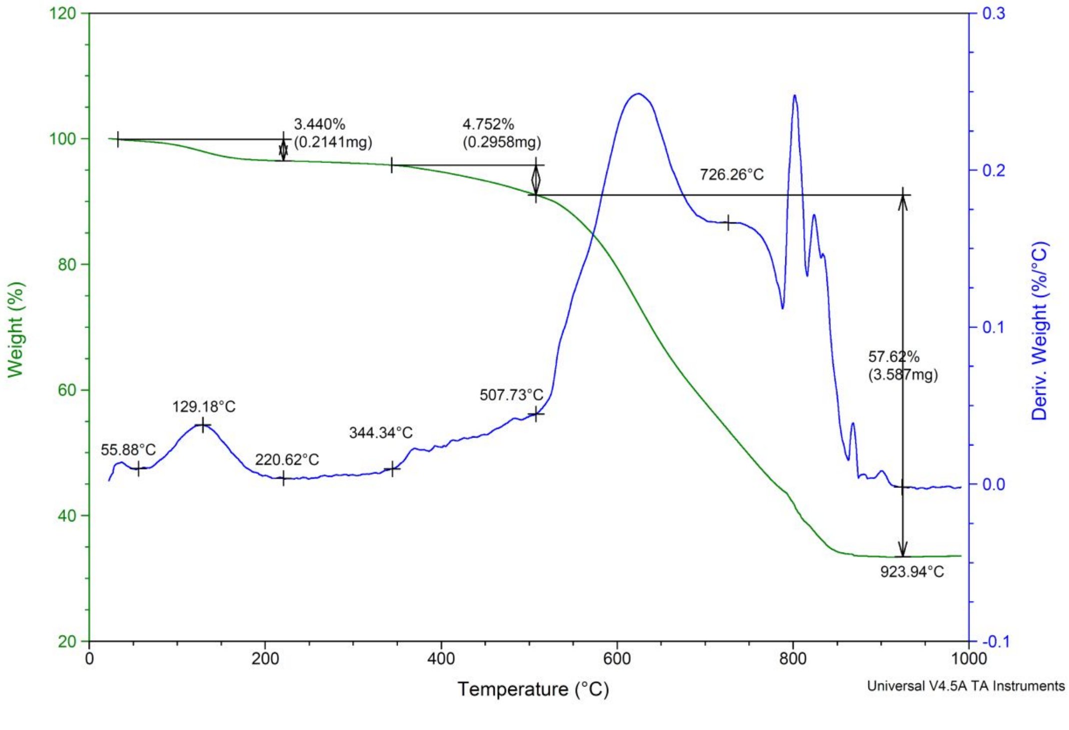

Figure S9: Thermogravimetric analysis of crystalline ZIF-UC-6 heated at 10 °C min^{-1} up to 1000 °C under argon. Weight (%) curve shown in green and derivative weight (%/°C) shown in blue. All data analysis was performed using the TA Instruments Universal Analysis software package.[7]

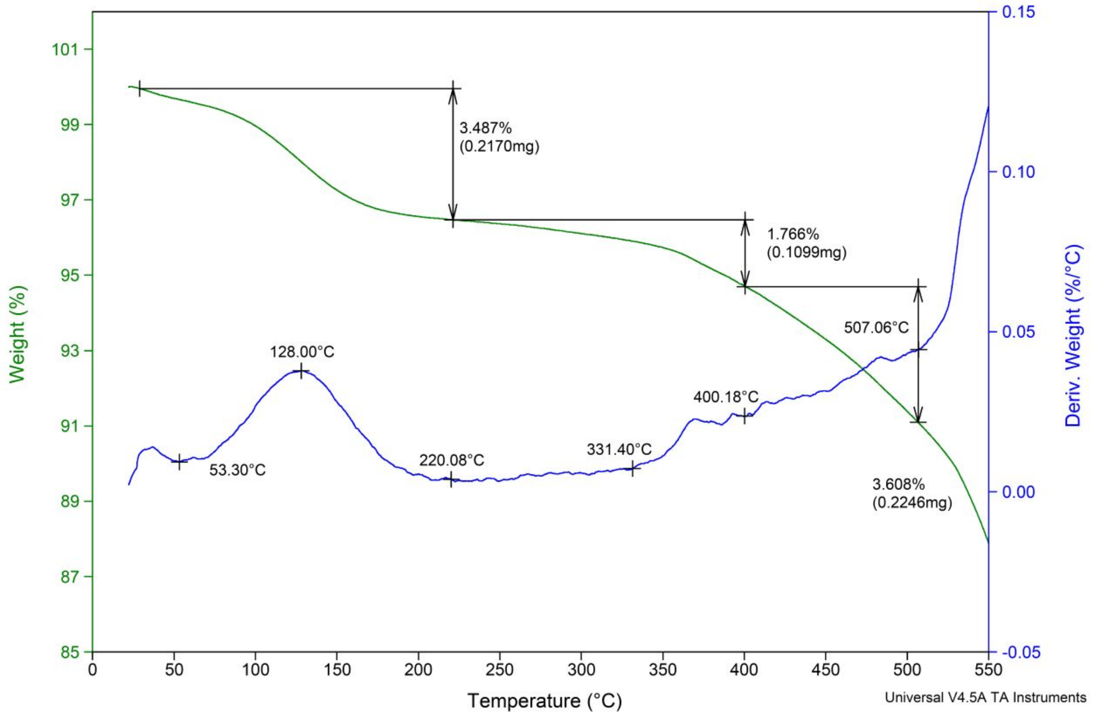

Figure S10: Thermogravimetric analysis of crystalline ZIF-UC-6 heated at 10 °C min^{-1} up to 550 °C under argon. Weight (%) curve shown in green and derivative weight (%/°C) shown in blue.

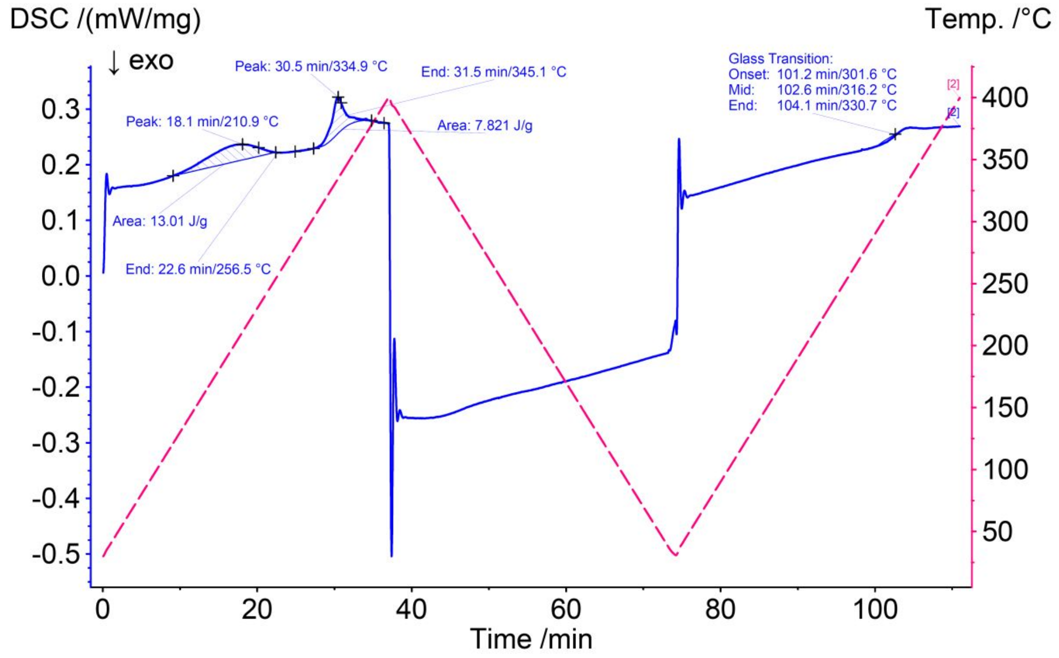

Figure S11: Differential scanning calorimetry (DSC) of crystalline ZIF-UC-6 heated to 400 °C, cooled to 30 °C and heated to 400 °C at 10 °C min^{-1} under argon. Heat flow curve shown in blue and temperature trace shown in pink. All data analysis was performed using the Netzsch Proteus® software package.[8]

Table S3: Melting thermodynamics of ZIF-UC-6 vs. reported values for ZIF-UC-5 and TIF-4

**ZIF**	**T_m (°C)**	**T_m (K)**	**ΔH_{fus} (J g^{-1})**	**ΔH_{fus} (kJ mol^{-1})**	**ΔS_{fus} (J K^{-1} mol^{-1})**
ZIF-UC-6	345	618	7.8	1.7	2.8
ZIF-UC-5[9]	428	701	11.7	2.5	3.6
TIF-4[9]	440	713	15.3	3.2	4.5

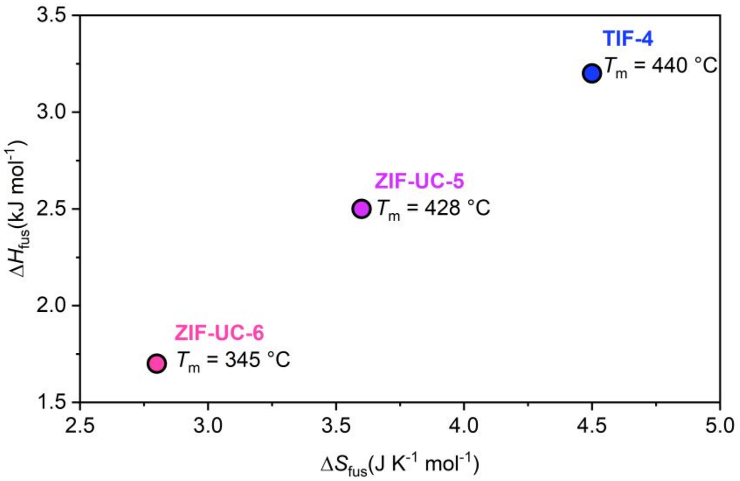

Figure S12: Enthalpy and entropy of fusion of ZIF-UC-6 compared to TIF-4 and ZIF-UC-5.[9] Values were calculated based on the method reported by Mason *et al*.[10] The molar enthalpy was calculated using the molecular weight for the ZIF formula unit.

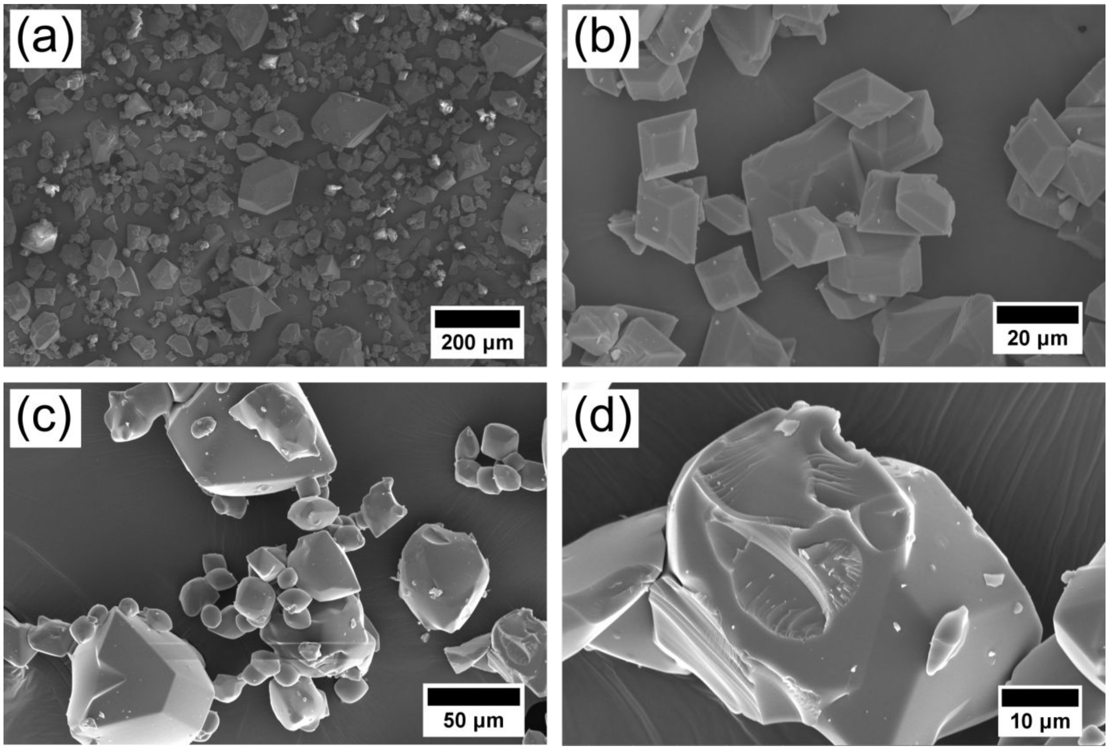

Figure S13: Scanning electron microscope images of crystalline ZIF-UC-6 (a) and (b) and a_gZIF-UC-6 (c) and (d). Crystalline ZIF-UC-6 exhibits sharp crystal facets on all particles. a_gZIF-UC-6 had more rounded particles with flow related striations (d).

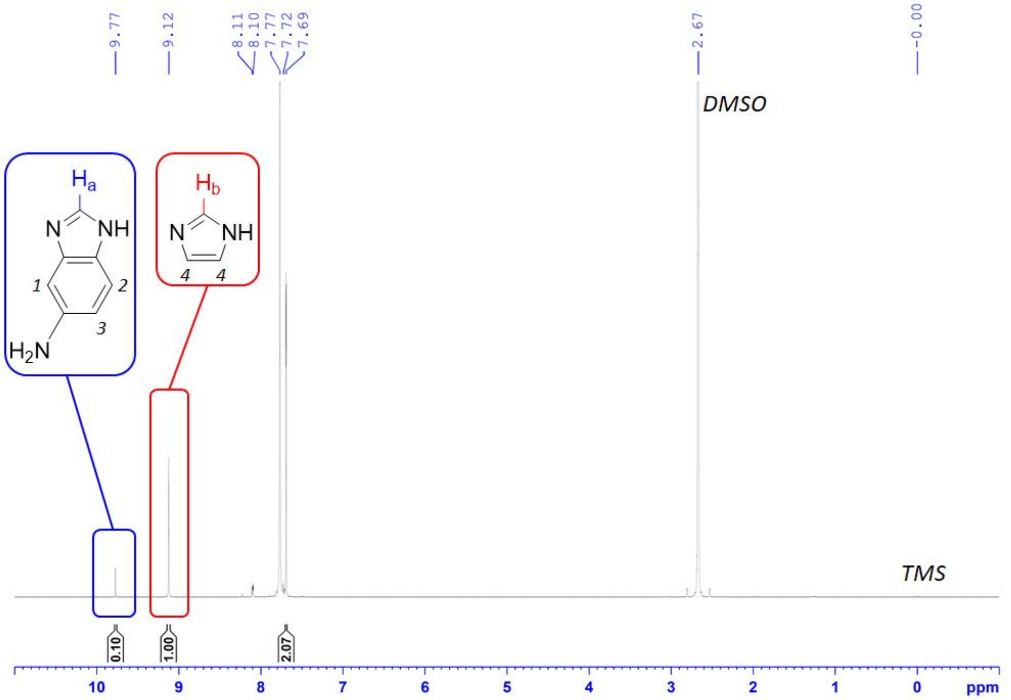

Figure S14: ^{1}H nuclear magnetic resonance spectrum of a_gZIF-UC-6. δH (500 MHz; DCl(35%)/D_2O:DMSO-d$_6$ (1:5); Me_4Si) 9.77 (1H, s, H_a), 9.12 (1H, s, H_b), 8.11 (1H, d, H_2), 8.10 (1H, s, H_1), 7.77 (H_2O/HCl), 7.72 (1H, d, H_3), 7.69 (2H, s, H_4) 2.67 (DMSO), 0.00 (TMS).

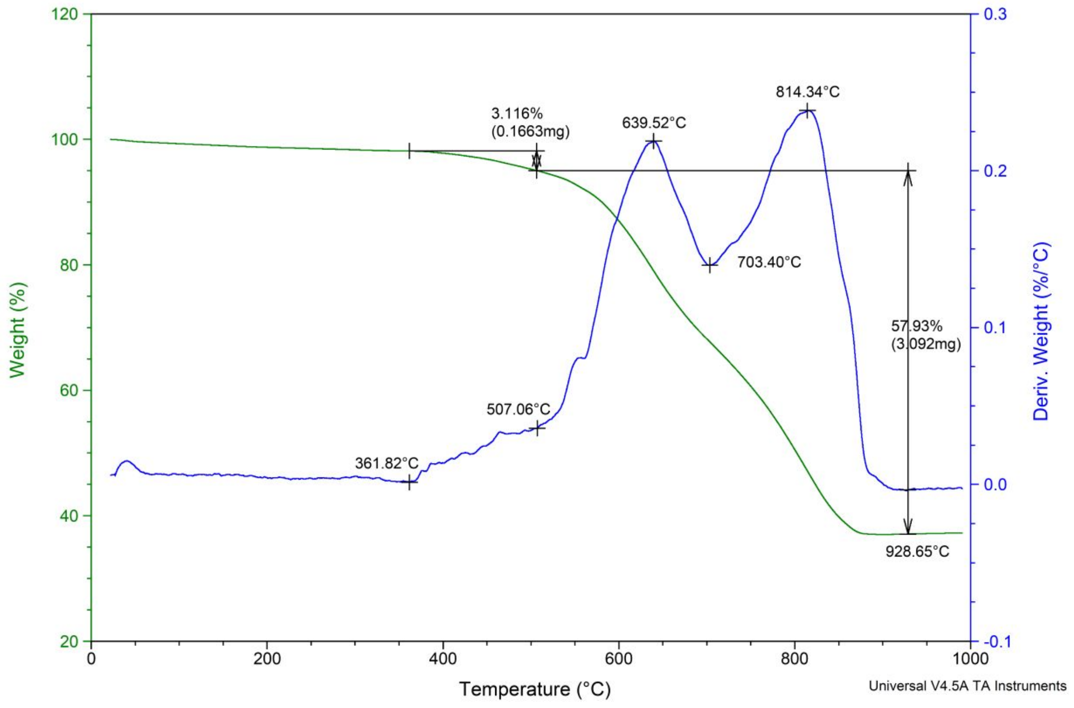

Figure S15: Thermogravimetric analysis of a_gZIF-UC-6 heated at 10 °C min^{-1} up to 1000 °C under argon. Weight (%) curve shown in green and derivative weight (%/°C) shown in blue.

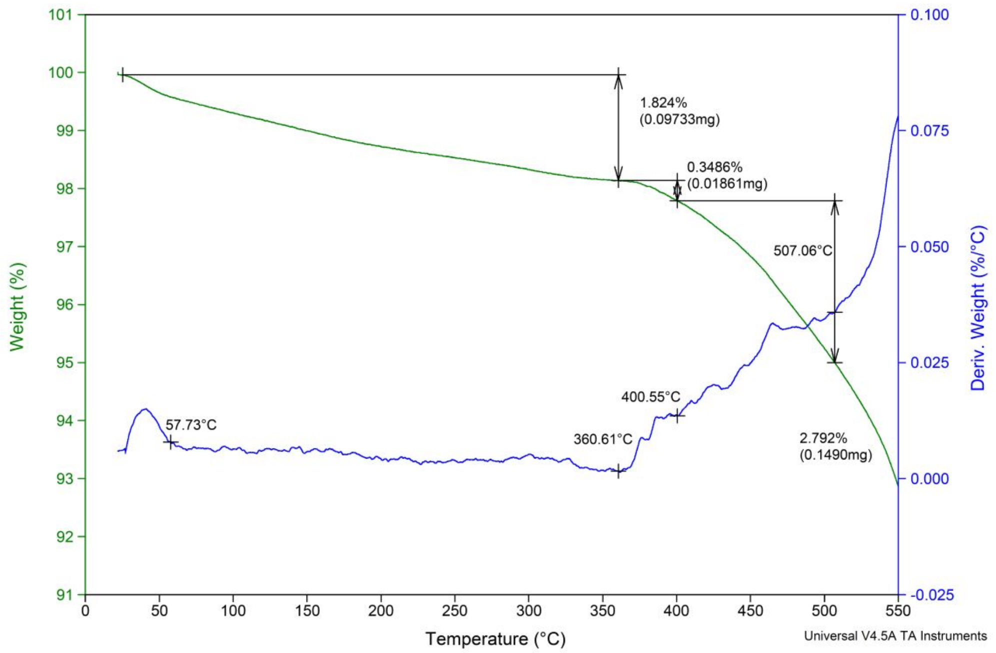

Figure S16: Thermogravimetric analysis of a_gZIF-UC-6 heated at 10 °C min^{-1} up to 550 °C under argon. Weight (%) curve shown in green and derivative weight (%/°C) shown in blue.

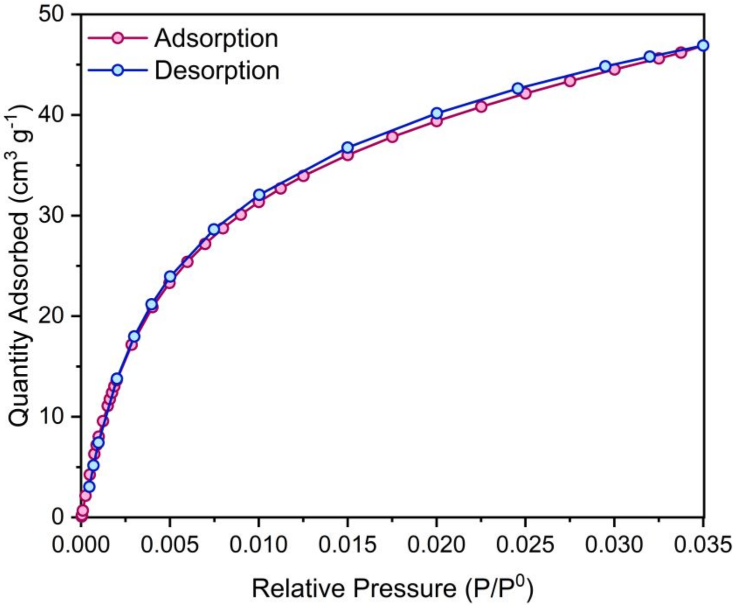

Figure S17: CO_2 adsorption (pink) and desorption (blue) isotherms collected on crystalline ZIF-UC-6 from 0-0.035 P/P^0. Maximum gas uptake was determined using the Micromeritics MicroActive software package.[11]

Table S4: CO_2 gas sorption results for crystalline ZIF-UC-6

Maximum CO_2 uptake (cm^3 g^{-1} STP)	46.90
Maximum CO_2 uptake (mmol g^{-1})	2.09

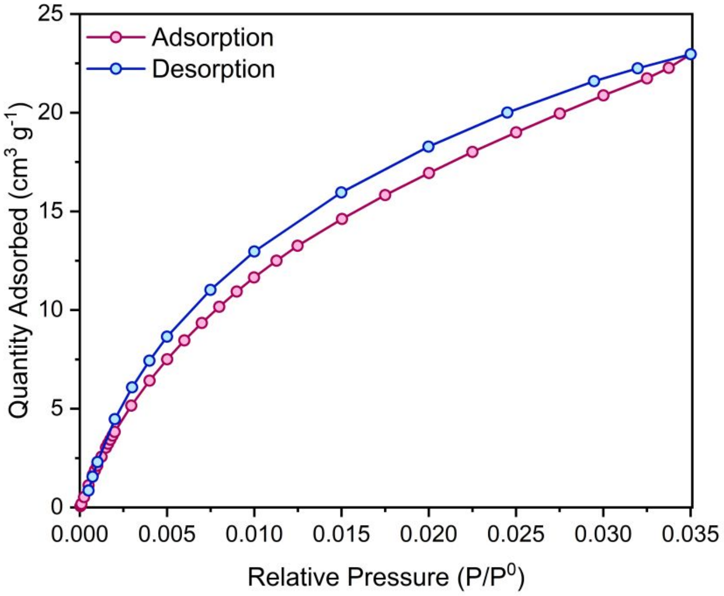

Figure S18: CO_2 adsorption (pink) and desorption (blue) isotherms collected on a_gZIF-UC-6 from 0-0.035 P/P^0. Maximum gas uptake was determined using the Micromeritics MicroActive software package.[11]

Table S5: CO_2 gas sorption results for a_gZIF-UC-6

Maximum CO_2 uptake (cm^3 g^{-1} STP)	22.96
Maximum CO_2 uptake (mmol g^{-1})	1.02

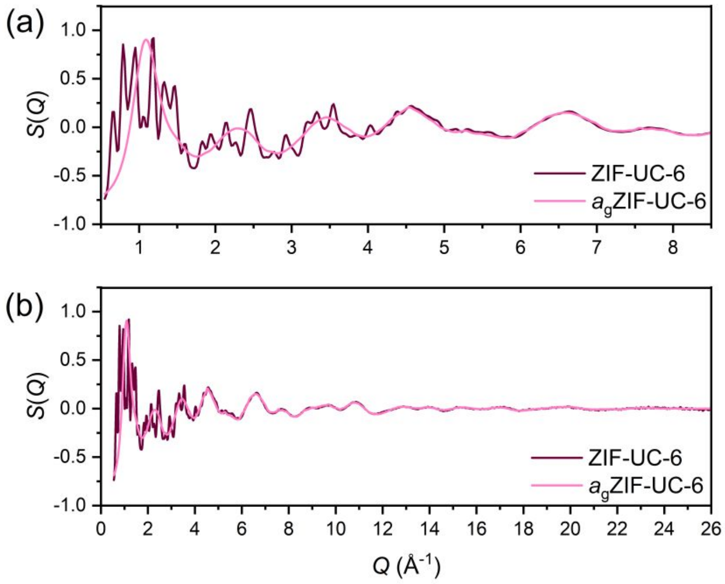

Figure S19: Total scattering structure factors of ZIF-UC-6 (dark pink) and a_gZIF-UC-6 (light pink). (a) Highlights differences in the low *Q* region between ZIF-UC-6 and a_gZIF-UC-6 resulting from differences in their long-range order. (b) Shows the total scattering data collected for both materials in the full *Q* range up to Q_{max} = 26 Å^{-1}. All processing of the total scattering data was performed using GudrunX following well documented procedures.[12–14]

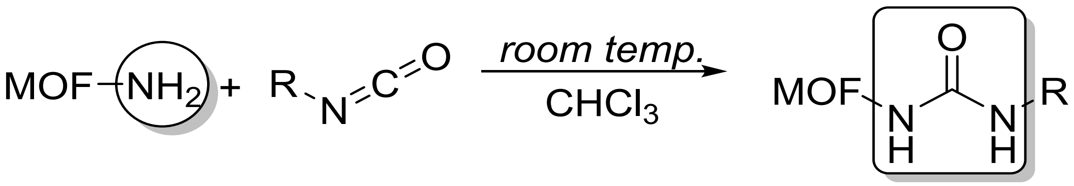

Figure S20: Reaction scheme of an amine-functionalized MOF with an isocyanate resulting in a urea functionalized MOF and no by-products.

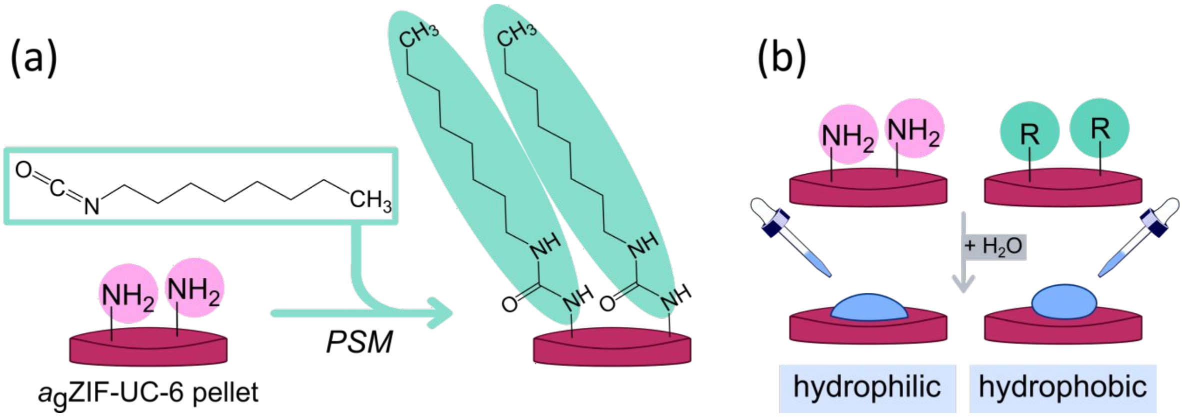

Figure S21: (a) Schematic of the surface modification of a_gZIF-UC-6 (amine groups highlighted in pink) with octyl isocyanate, resulting in the formation of urea functionalities with hydrophobic carbon chains protruding from the glass surface (highlighted in green). (b) Schematic of the proposed change in the surface hydrophobicity by PSM: amine groups (pink) create a hydrophilic surface whilst carbon chains (green) create a hydrophobic surface, resulting in a change in the surface water contact angle.

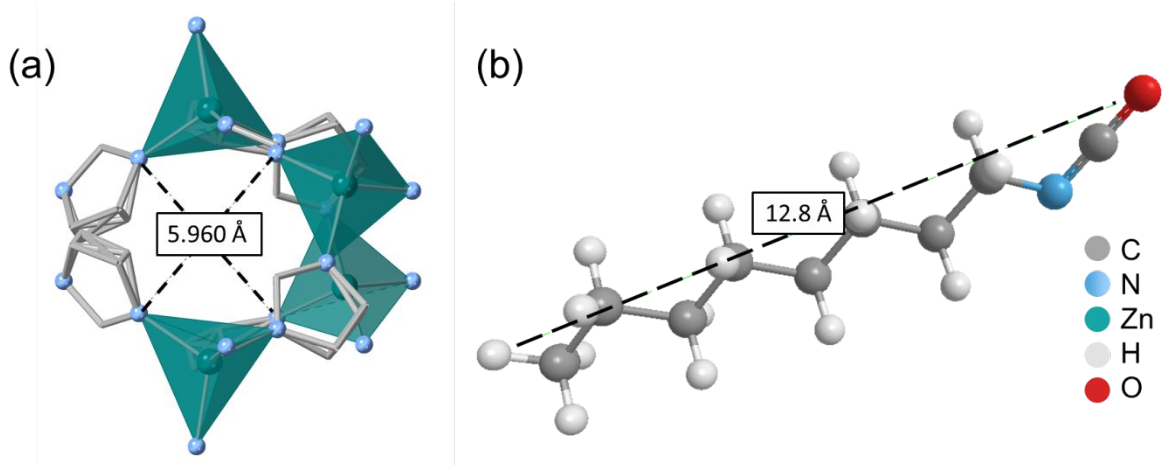

Figure S22: (a) Pore diameter of ZIF-UC-6 measured in CrystalMaker® X, Version 10.5.4, CrystalMaker Software Limited. (b) Length of an octyl isocyanate molecule (12.8 Å) calculated using Chem3D 19.0™, PerkinElmer Informatics. This is significantly larger than the pore size of ZIF-UC-6, thus PSM will only occur on the material surface. Atoms shown are carbon – grey, nitrogen – blue, zinc – green, hydrogen – white and oxygen – red.

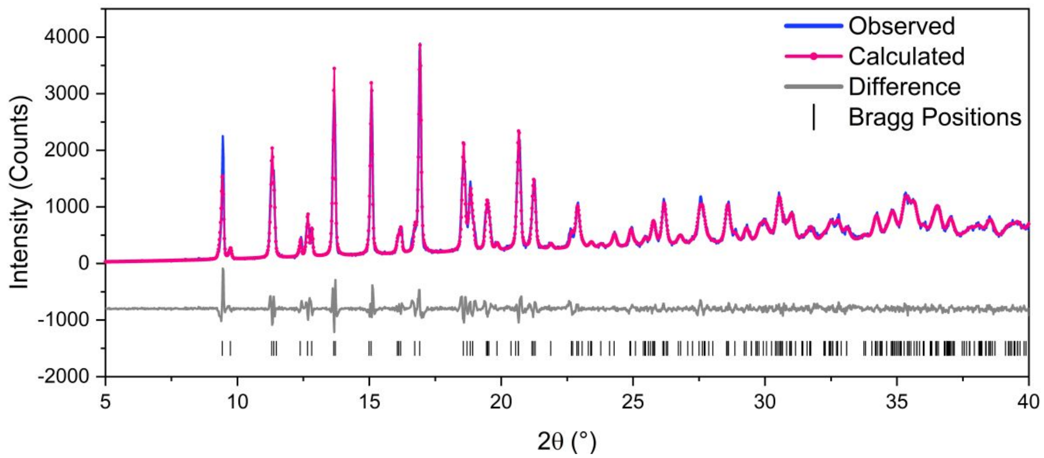

Figure S23: Pawley refinement of powder X-ray diffraction data of ZIF-UC-6 after modification with octyl isocyanate. Initial parameters were obtained from the CIF for ZIF-UC-6 reported in this work. Difference curve and Bragg positions are offset on the y axis for clarity.

Table S6: Data from Pawley refinement of modified ZIF-UC-6

R_{wp}	Space Group	Refined Powder Lattice Parameters	Single Crystal Lattice Parameters
8.943	*Pbca*	*a* = 15.410(3) Å	*a* = 15.839(2) Å
		b = 15.541(3) Å	*b* = 15.599(2) Å
		c = 18.166(4) Å	*c* = 17.984(2) Å
		α = β = γ = 90 °	α = β = γ = 90 °

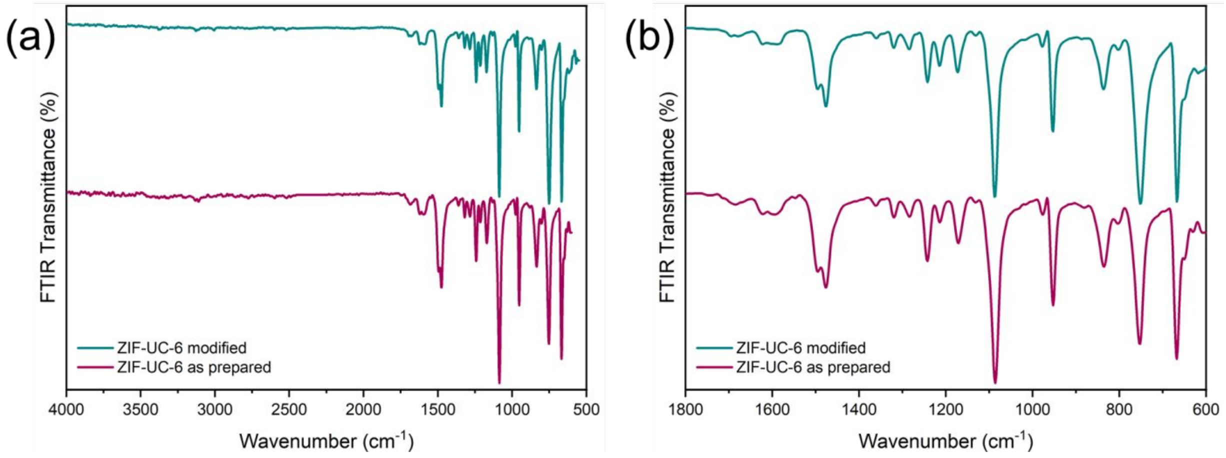

Figure S24: Fourier transform infrared (FTIR) spectra of ZIF-UC-6 (pink) and modified ZIF-UC-6 (green) from 550 to 4000 cm^{-1} (a) and from 600 to 1800 cm^{-1}.

(a)

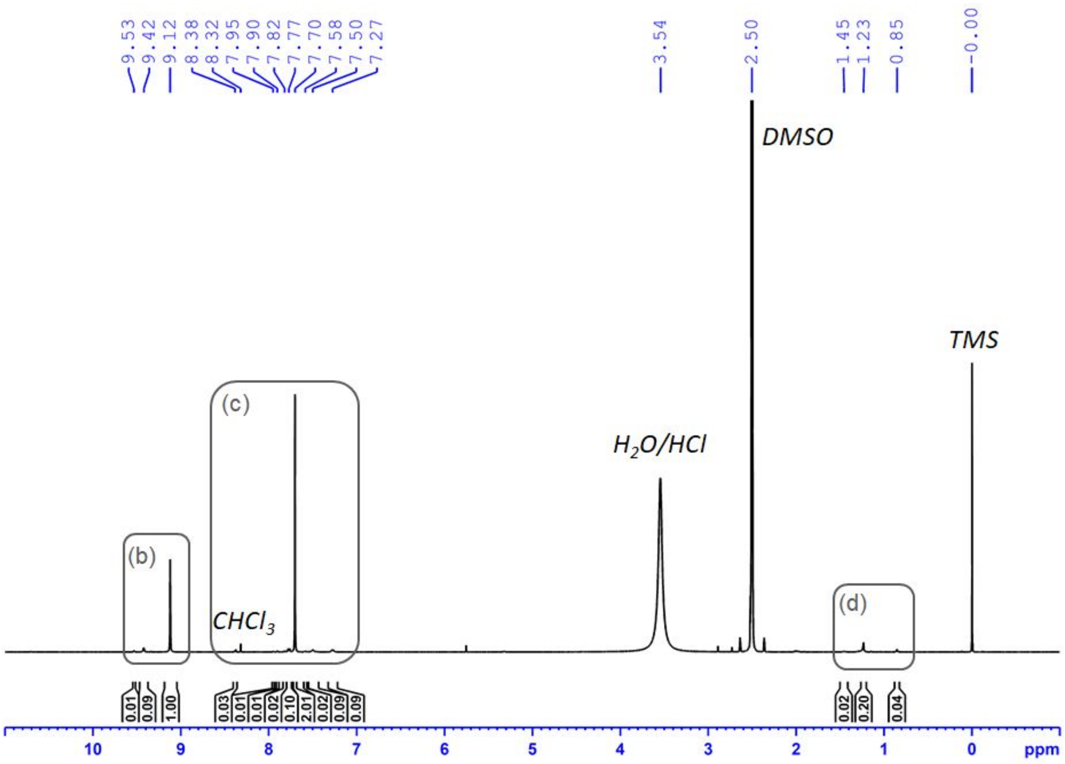
9.53
9.42
9.12
8.38
8.32
7.95
7.90
7.82
7.77
7.70
7.58
7.50
7.27
3.54
2.50
1.45
1.23
0.85
0.00
DMSO
TMS
H2O/HCl
(b)
(c)
(d)
CHCl3
0.01
0.09
1.00
0.03
0.01
0.01
0.02
0.10
2.01
0.02
0.02
0.09
0.09
0.02
0.20
0.04
10
9
8
7
6
5
4
3
2
1
0
ppm

(b)

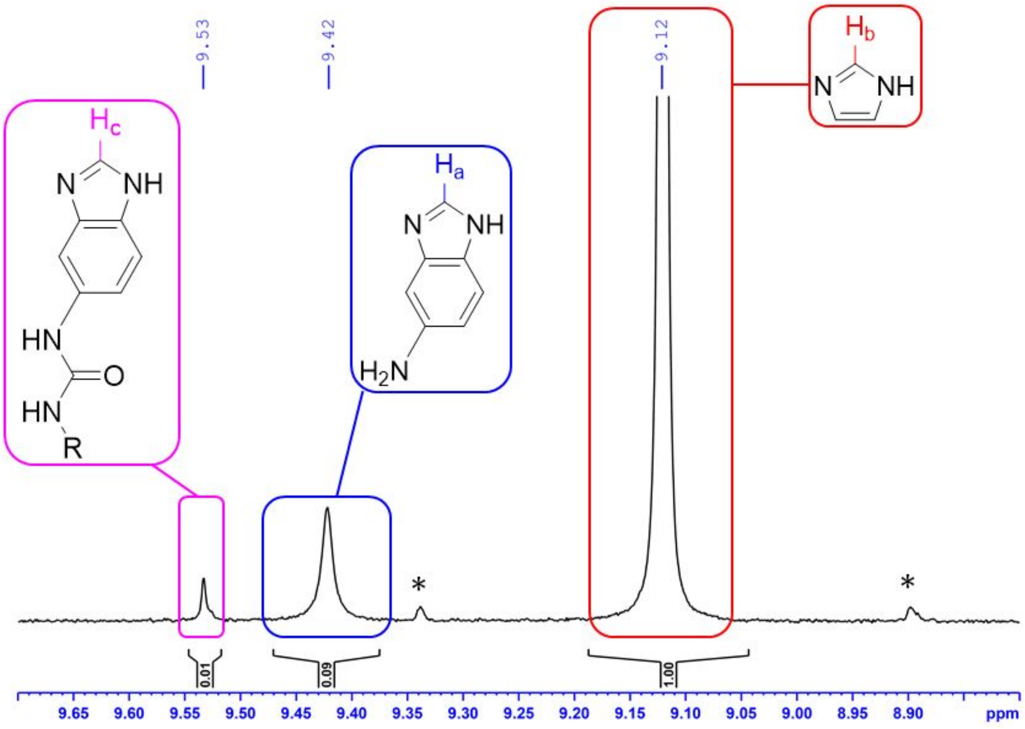
9.53
9.42
9.12
Hc
N
NH
HN
O
HN
R
Ha
N
NH
H2N
Hb
N
NH
*
*
0.01
0.09
1.00
9.65
9.60
9.55
9.50
9.45
9.40
9.35
9.30
9.25
9.20
9.15
9.10
9.05
9.00
8.95
8.90
ppm

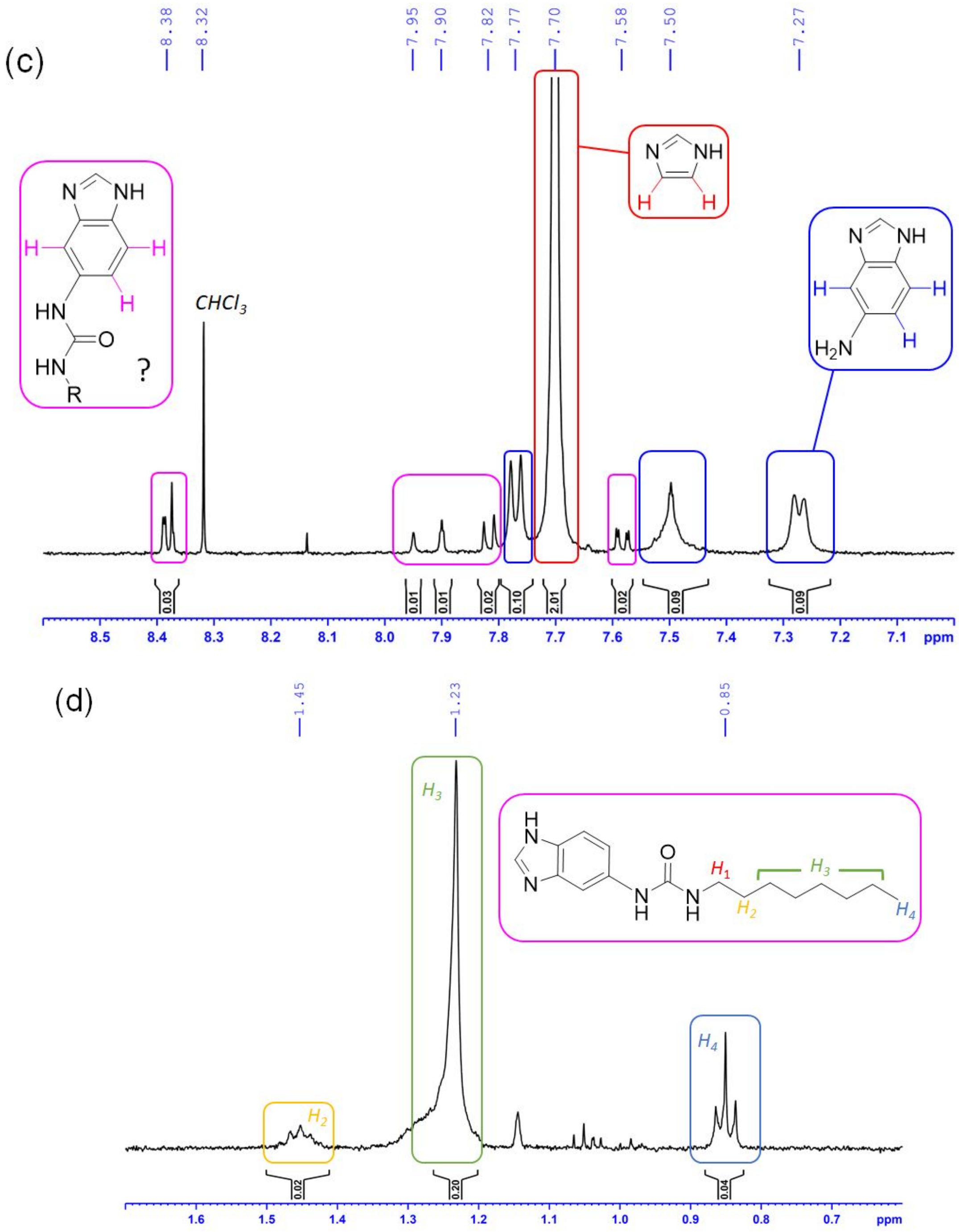

Figure S25: ^{1}H nuclear magnetic resonance spectrum of modified ZIF-UC-6. (a) Full ^{1}H NMR spectrum with positions of figures (b), (c) and (d) highlighted in grey. (b) Imidazole proton region of ^{1}H NMR, imidazole satellites marked by asterisks. (c) Aromatic region of ^{1}H NMR. Molecules shown in figures (b) and (c) are imidazole – red and 5-aminobenzimidazole – blue. Peaks with the correct position and integration for the modified linker are highlighted in pink although it is unlikely that all the peaks highlighted in (c) are due to the modified linker. (d) Alkyl chain region of ^{1}H NMR. Proton environments shown are H_1 – red (masked by H_2O/HCl), H_2 – yellow, H_3 – green and H_4 – blue. δH (500 MHz; DCl(35%)/D_2O:DMSO-d_6 (dilute conditions); Me_4Si) 9.53 (1H, s, H_c), 9.42 (1H, s, H_a), 9.12 (1H, s, H_b), 8.32 ($CHCl_3$), 8.38, 7.95, 7.90, 7.82, 7.58 (possible aromatic environments on modified linker), 7.77 (1H, d, 5abIm – aromatic proton), 7.70 (2H, s, Im – aromatic proton), 7.50 (1H, s, 5abIm – aromatic proton), 7.27 (1H, d, 5abIm – aromatic proton), 3.54 (H_2O/HCl), 2.50 (DMSO), 1.45 (2H, m, H_2), 1.23 (10H, m – methylene envelope, H_3), 0.85 (3H, t, H_4), 0.00 (TMS).

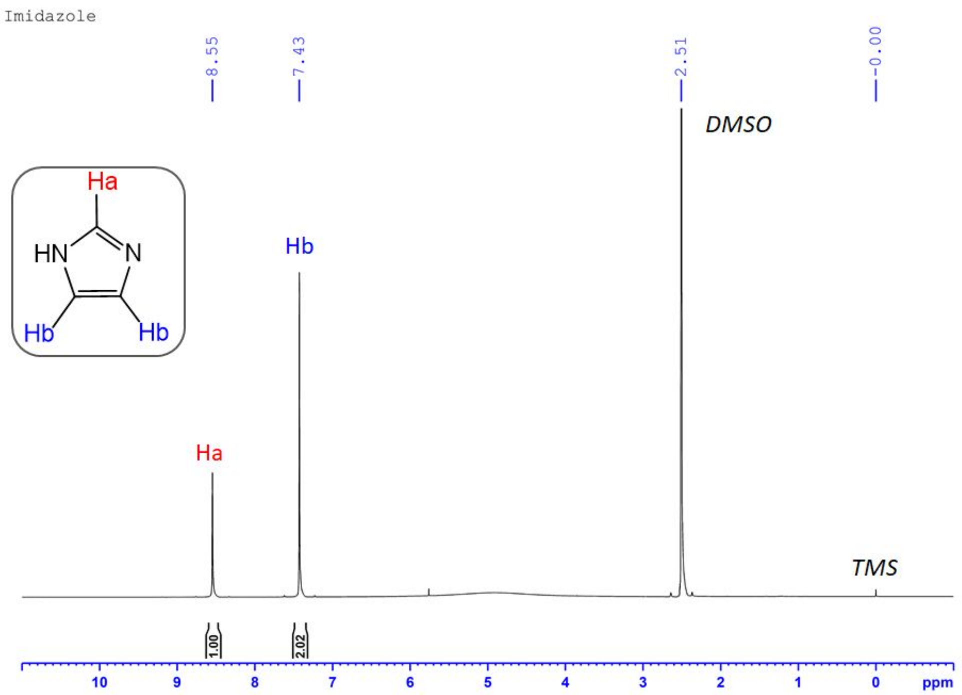

Figure S26: ^{1}H nuclear magnetic resonance spectrum of imidazole. δH (500 MHz; DCl(35%)/D_2O:DMSO-d_6 (dilute conditions); Me_4Si) 8.55 (1H, s, H_a), 7.43 (2H, s, H_b), 2.51 (DMSO), 0.00 (TMS).

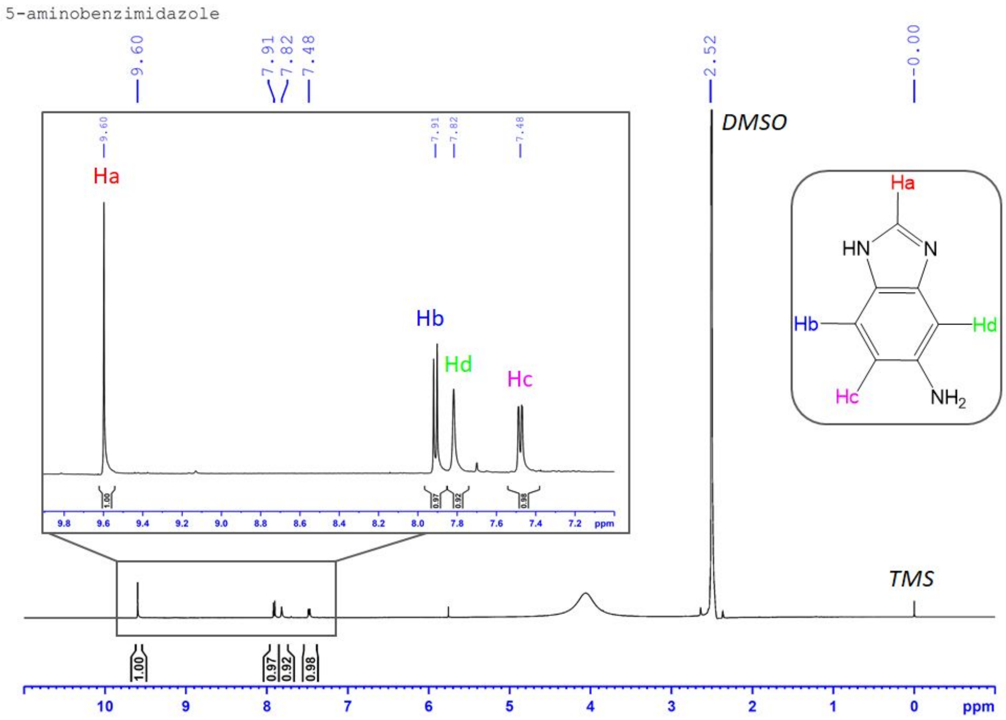

Figure S27: ^{1}H nuclear magnetic resonance spectrum of 5-aminobenzimidazole. δH (500 MHz; DCl(35%)/D_2O:DMSO-d_6 (dilute conditions); Me_4Si) 9.60 (1H, s, H_a), 7.91 (1H, d, H_b), 7.82 (1H, s, H_d), 7.48 (1H, d, H_c), 2.52 (DMSO), 0.00 (TMS).

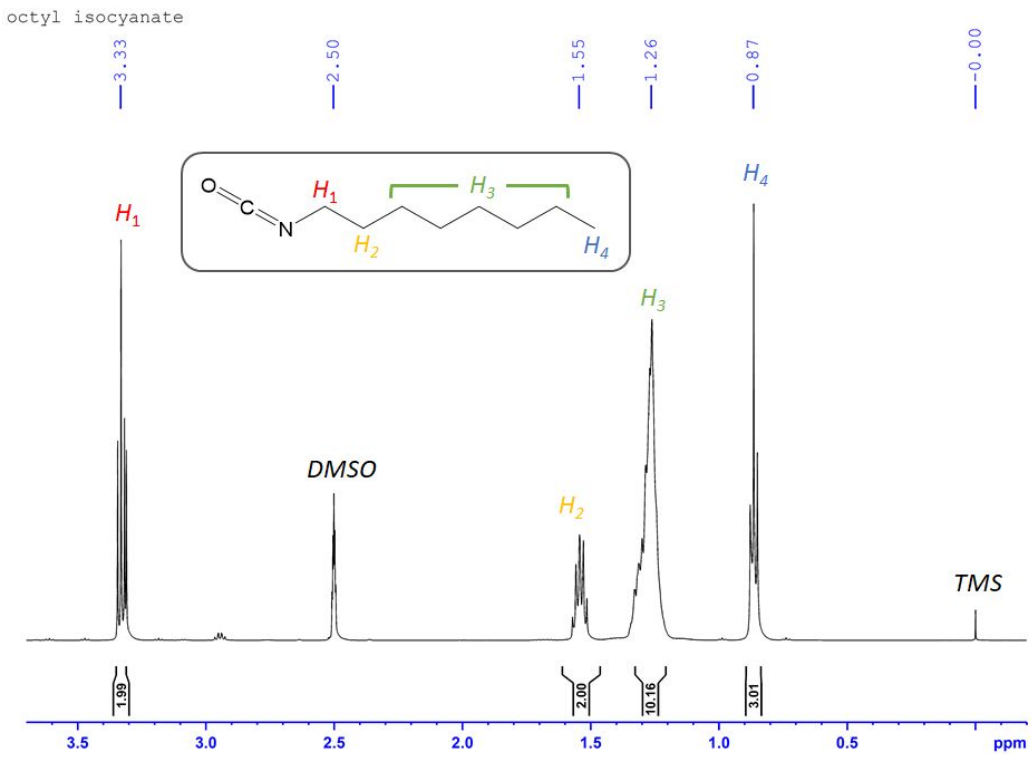

Figure S28: ^{1}H nuclear magnetic resonance spectrum of octyl isocyanate. δH (500 MHz; DMSO-d_6; Me_4Si) 3.33 (2H, t, H_1), 2.50 (DMSO), 1.55 (2H, m, H_2), 1.26 (10H, m – methylene envelope, H_3), 0.87 (3H, t, H_4), 0.00 (TMS).

Table S7: CHN microanalysis results for crystalline ZIF-UC-6

Sample	Mass (mg)	wt.% C	wt.% H	wt.% N
ZIF-UC-6	1.3013	37.77	2.99	26.02
ZIF-UC-6	1.2633	37.48	2.91	26.05
ZIF-UC-6	1.4597	37.29	2.91	25.91
Mean (ESD)	-	37.51 (0.24)	2.94 (0.05)	25.99 (0.07)
Predicted	-	38.21	3.12	27.72

Table S8: CHN microanalysis results for modified crystalline ZIF-UC-6

Sample	Mass (mg)	wt.% C	wt.% H	wt.% N
ZIF-UC-6 PSM	1.335	38.13	3	26.48
ZIF-UC-6 PSM	1.3012	37.8	2.93	26.35
ZIF-UC-6 PSM	1.4187	37.96	2.94	26.47
Mean (ESD)	-	37.96 (0.17)	2.96 (0.04)	26.43 (0.07)
Predicted	-	38.66	3.19	27.45

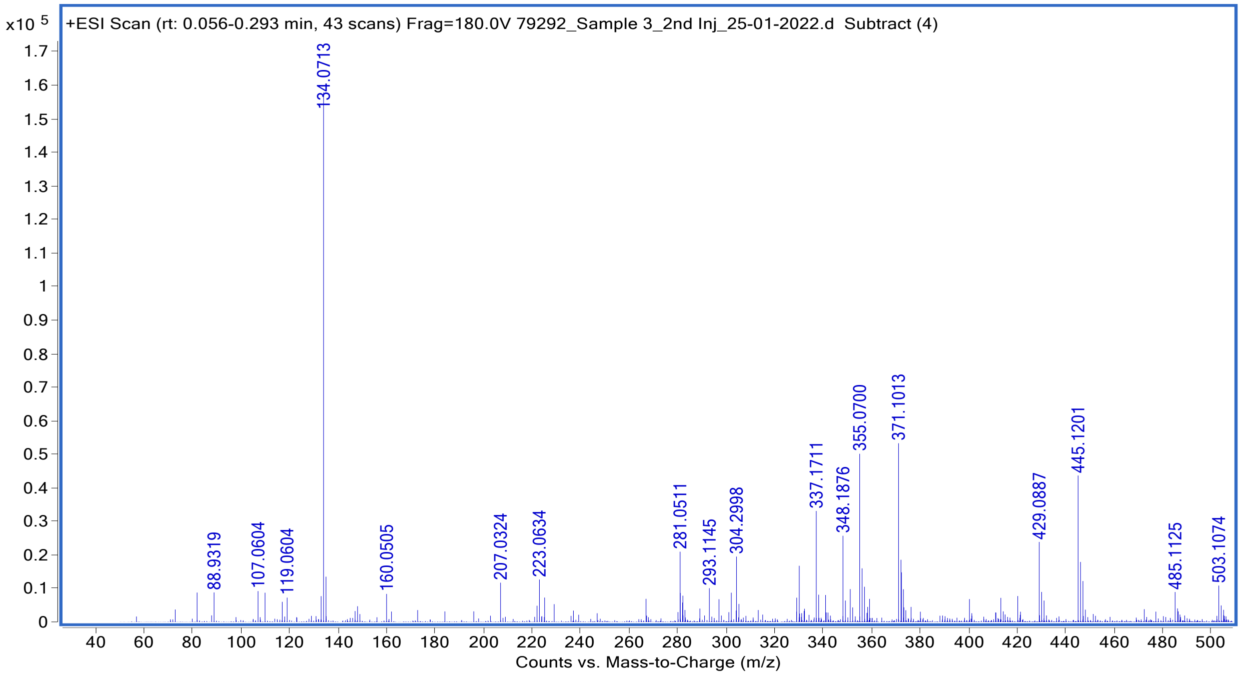

Figure S29: Mass spectrum of digested modified crystalline ZIF-UC-6 powder. The major peak at *m/z* = 134.0713 corresponds to unreacted 5-aminobenzimidazole. As the modified linker makes up < 1% of the material, it has a low intensity but is visible below.

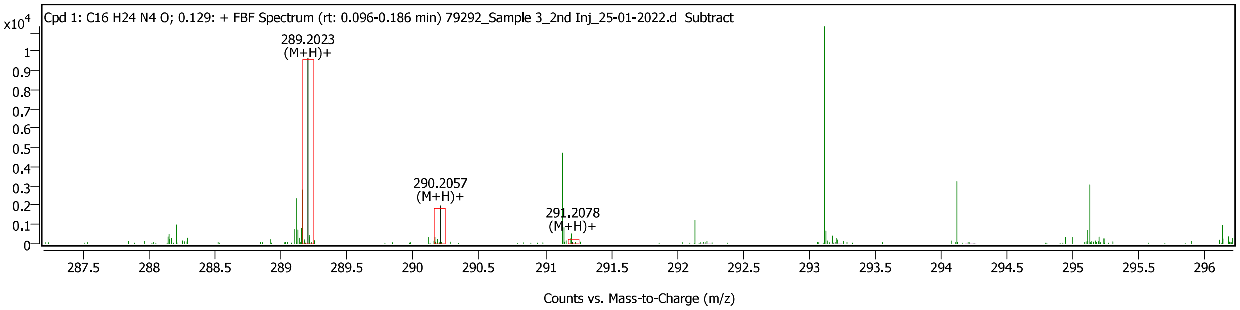

Figure S30: Portion of the mass spectrum of digested modified crystalline ZIF-UC-6 powder focused on the molecular ion peak for the modified linker, which has formula $C_{16}H_{24}N_4O$ $[C_{16}H_{24}N_4O]H^+$ (calc: *m/z* = 289.2028; found: *m/z* = 289.2023; 1.7 ppm).

(a)

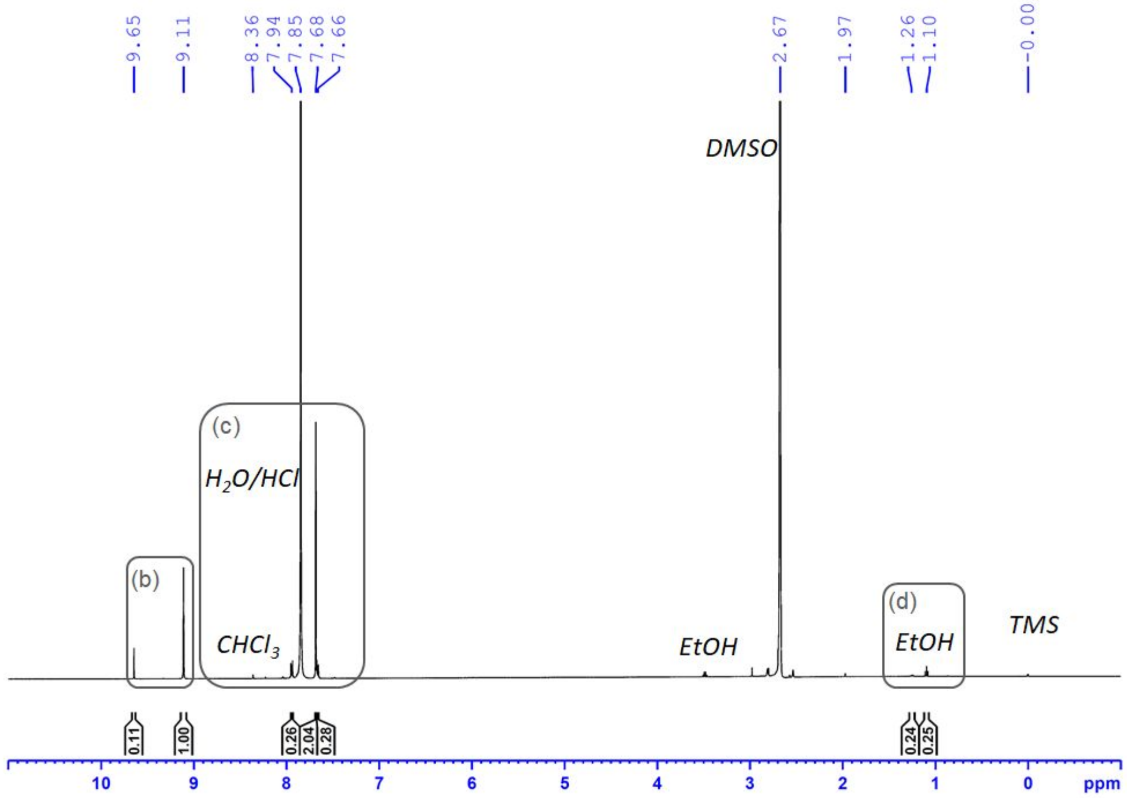
9.65
9.11
8.36
7.94
7.85
7.68
7.66
2.67
1.97
1.26
1.10
-0.00
DMSO
(c)
H2O/HCl
(b)
CHCl3
EtOH
(d)
EtOH
TMS
0.11
1.00
0.26
2.04
0.28
0.24
0.25
10
9
8
7
6
5
4
3
2
1
0
ppm

(b)

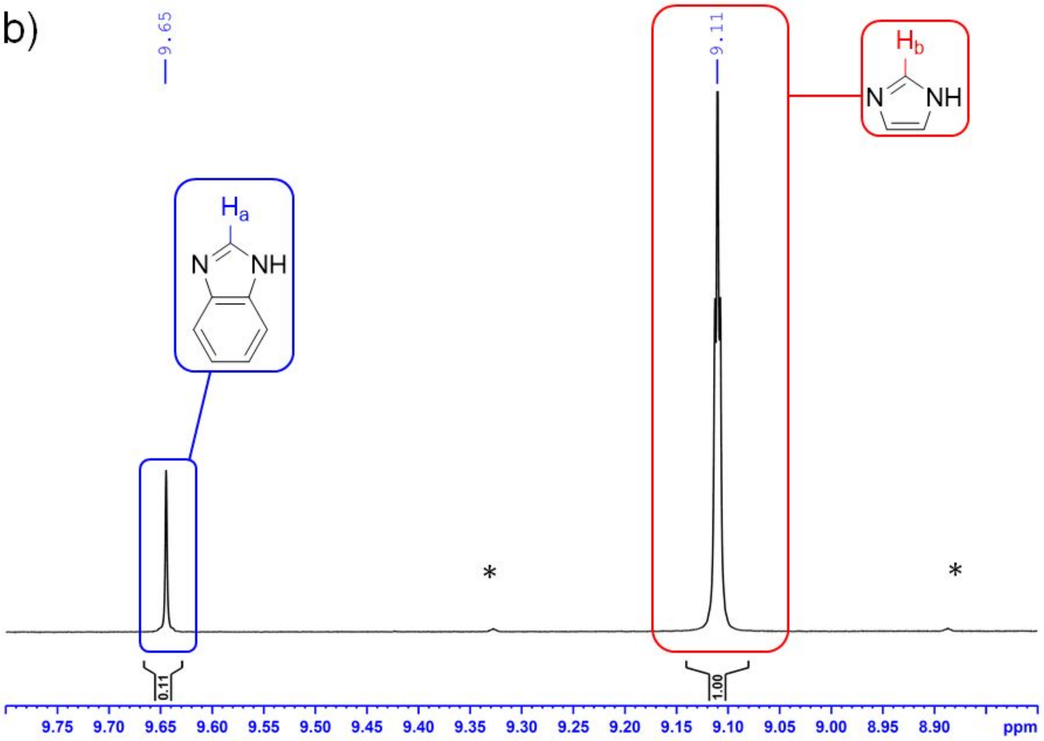
9.65
9.11
Hb
N
NH
Ha
N
NH
*
*
0.11
1.00
9.75
9.70
9.65
9.60
9.55
9.50
9.45
9.40
9.35
9.30
9.25
9.20
9.15
9.10
9.05
9.00
8.95
8.90
ppm

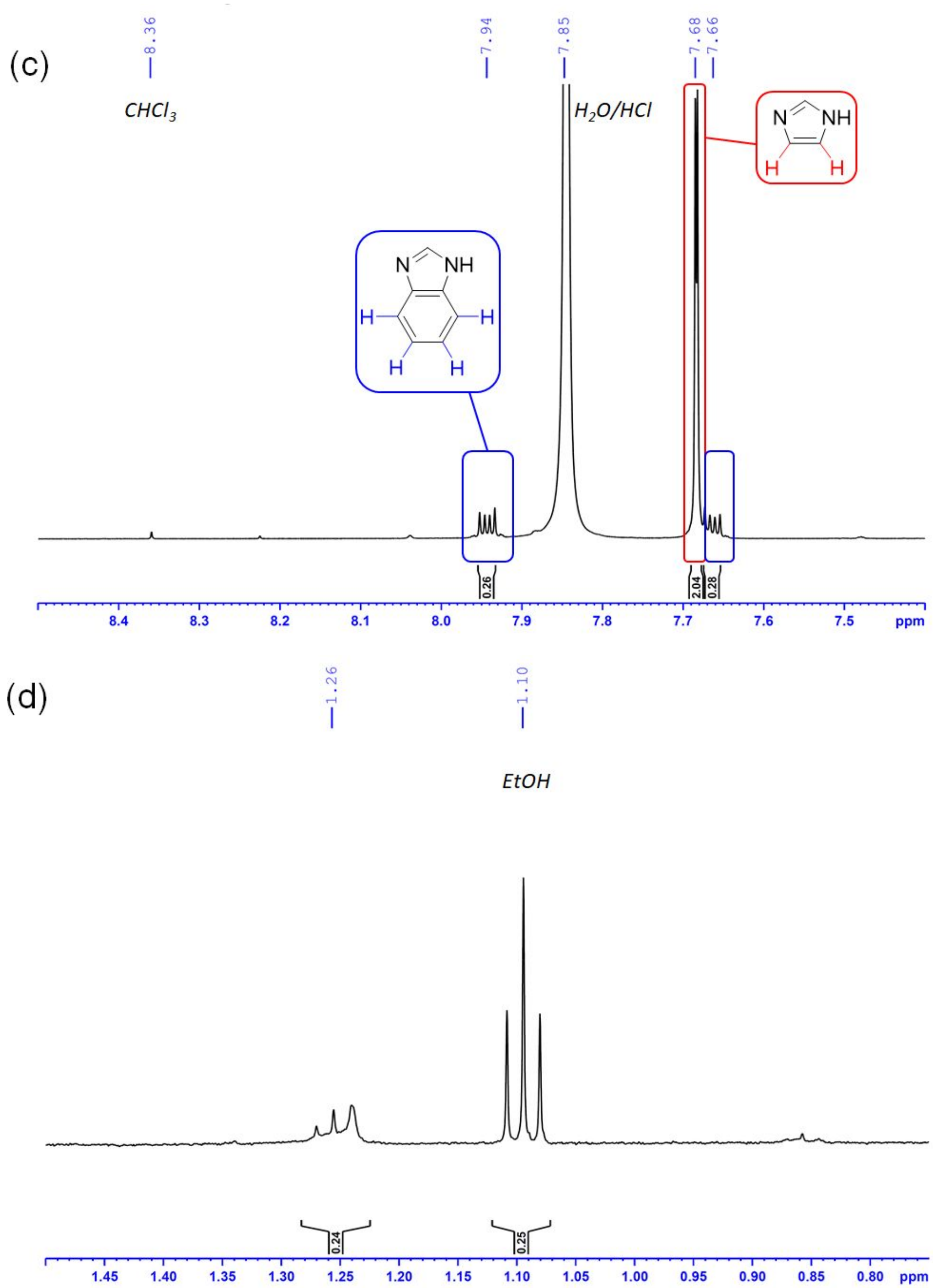

Figure S31: ^{1}H nuclear magnetic resonance spectrum of ZIF-62 control experiment. (a) Full ^{1}H NMR spectrum with positions of figures (b), (c) and (d) highlighted in grey. (b) Imidazole proton region of ^{1}H NMR, imidazole satellites marked by asterisks. (c) Aromatic region of ^{1}H NMR. Molecules shown in figures (b) and (c) are imidazole – red and benzimidazole – blue. No evidence for the formation of a modified linker was observed. (d) Alkyl chain region of ^{1}H NMR. δH (500 MHz; DCl(35%)/D_2O:DMSO-d_6 (1:5); Me_4Si) 9.65 (1H, s, H_a), 9.11 (1H, s, H_b), 8.36 ($CHCl_3$), 7.94, 7.66 (bIm aromatic environments), 7.85 (H_2O/HCl), 7.68 (2H, s, Im – aromatic proton), 2.67 (DMSO), 1.26 (impurity - possibly trace quantities of unreacted octyl isocyanate), 1.10 (ethanol), 0.00 (TMS). It should be noted that, ideally, the same solvent conditions as for modified ZIF-UC-6 would have been used here. However, ZIF-62 was not soluble in dilute acidic conditions so a higher concentration of DCl had to be used.

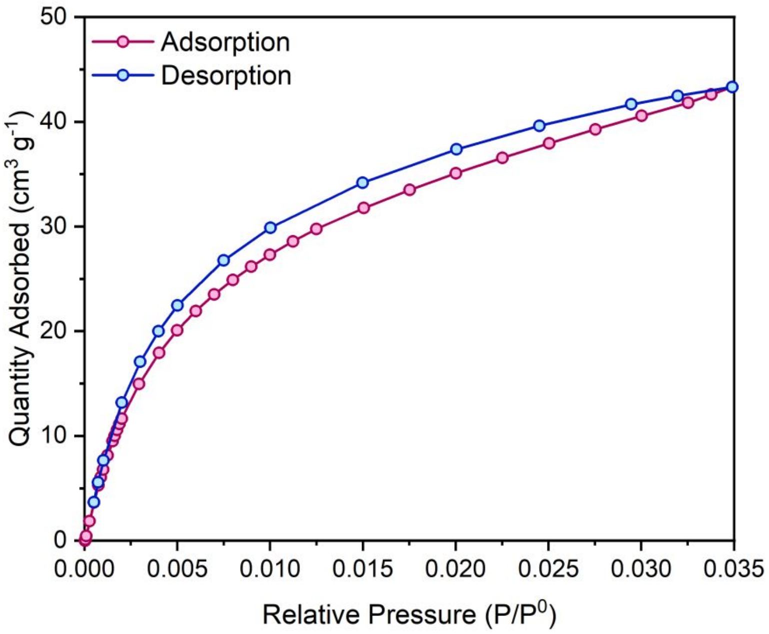

Figure S32: CO_2 adsorption (pink) and desorption (blue) isotherms collected on modified crystalline ZIF-UC-6 from 0-0.035 P/P^0. Maximum gas uptake was determined using the Micromeritics MicroActive software package.[11]

Table S9: CO_2 gas sorption results for modified crystalline ZIF-UC-6

Maximum CO_2 uptake (cm^3 g^{-1} STP)	43.32
Maximum CO_2 uptake (mmol g^{-1})	1.93

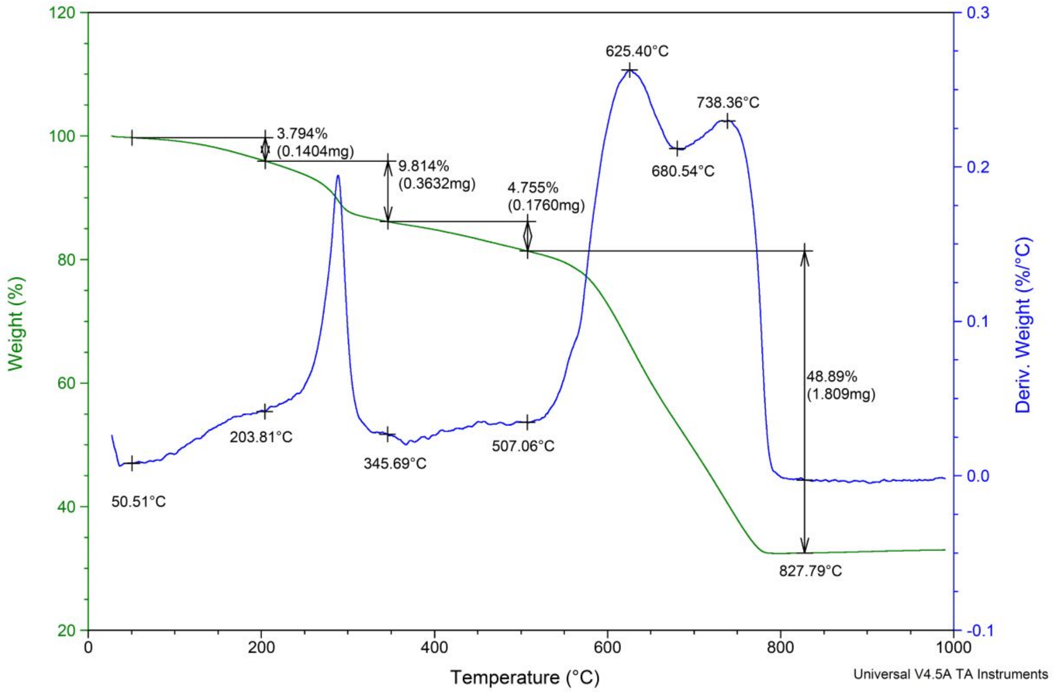

Figure S33: Thermogravimetric analysis of crystalline ZIF-UC-6 after modification with octyl isocyanate, heated at 10 °C min^{-1} up to 1000 °C under argon. Weight (%) curve shown in green and derivative weight (%/°C) shown in blue.

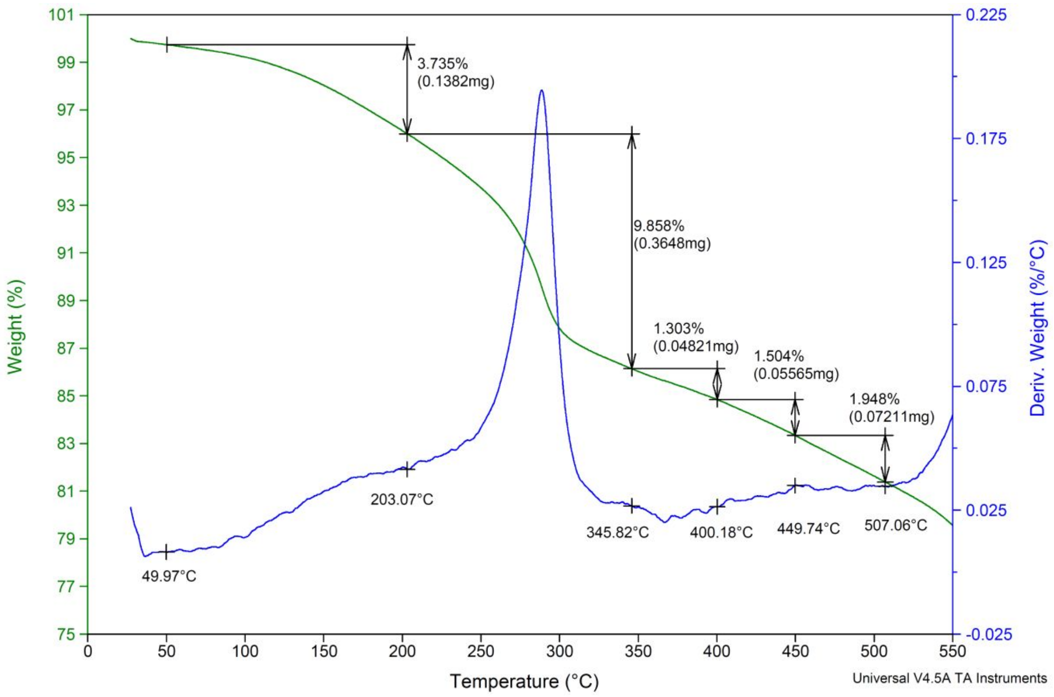

Figure S34: Thermogravimetric analysis of crystalline ZIF-UC-6 after modification with octyl isocyanate, heated at 10 °C min^{-1} up to 550 °C under argon. Weight (%) curve shown in green and derivative weight (%/°C) shown in blue.

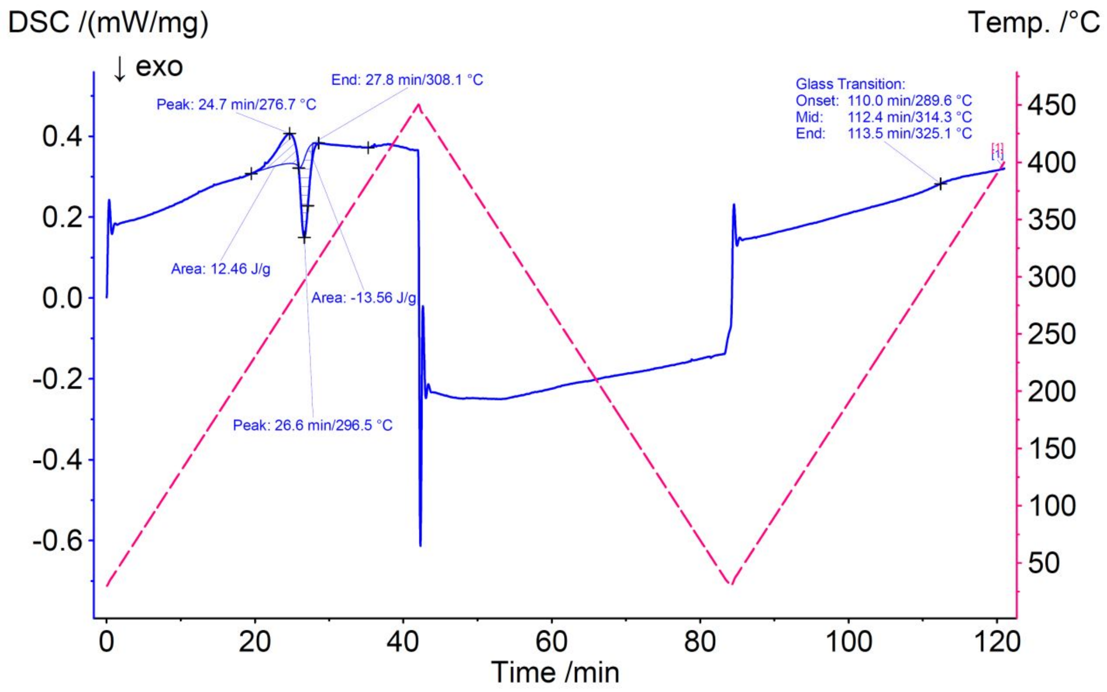

***Figure S35**:* Differential scanning calorimetry of crystalline ZIF-UC-6 after modification with octyl isocyanate, heated to 450 °C, cooled to 30 °C and heated to 400 °C at 10 °C min^{-1} under argon. Heat flow curve shown in blue and temperature trace shown in pink.

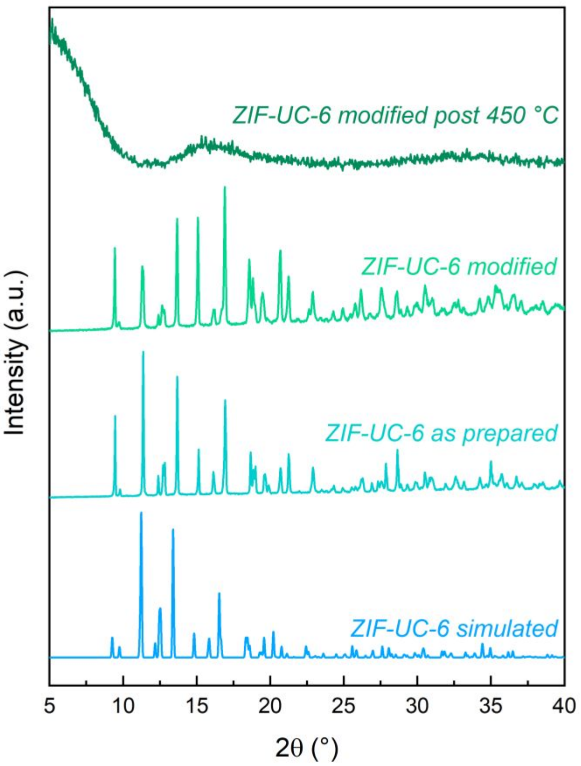

Figure S36: Powder X-ray diffraction patterns between 5-40° of as prepared ZIF-UC-6 (cyan), modified ZIF-UC-6 (light green) and, modified ZIF-UC-6 post 450 °C (dark green, only diffuse scattering observed) compared to the simulated diffraction pattern of ZIF-UC-6 (light blue) obtained from CIF reported in this work.

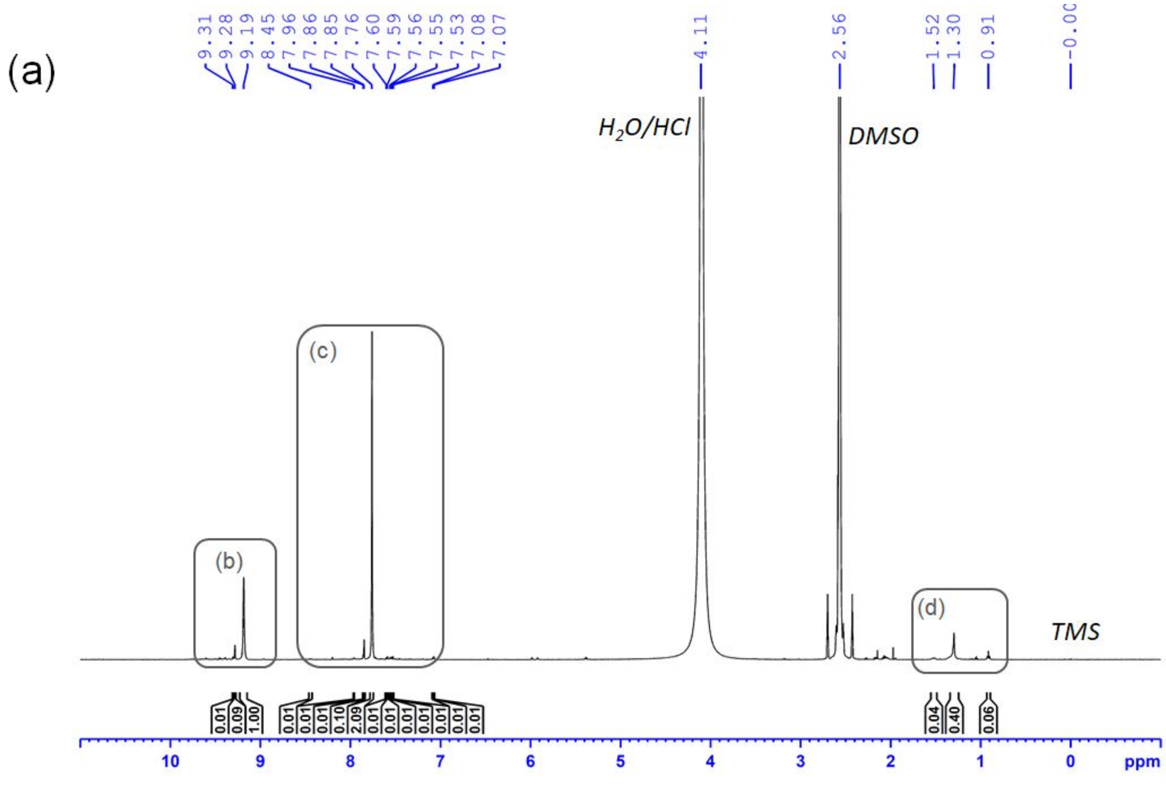

(b)

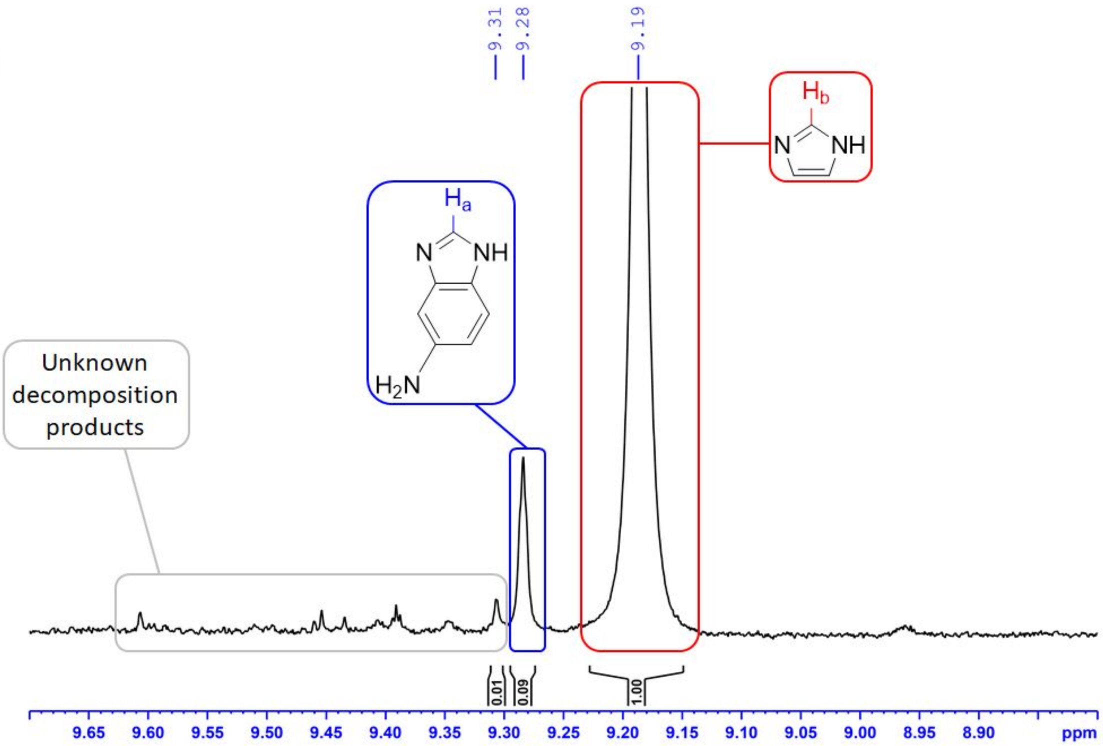

9.31
9.28
9.19
Hb
N
NH
Ha
N
NH
H2N
Unknown
decomposition
products
0.01
0.09
1.00
9.65 9.60 9.55 9.50 9.45 9.40 9.35 9.30 9.25 9.20 9.15 9.10 9.05 9.00 8.95 8.90 ppm

(c)

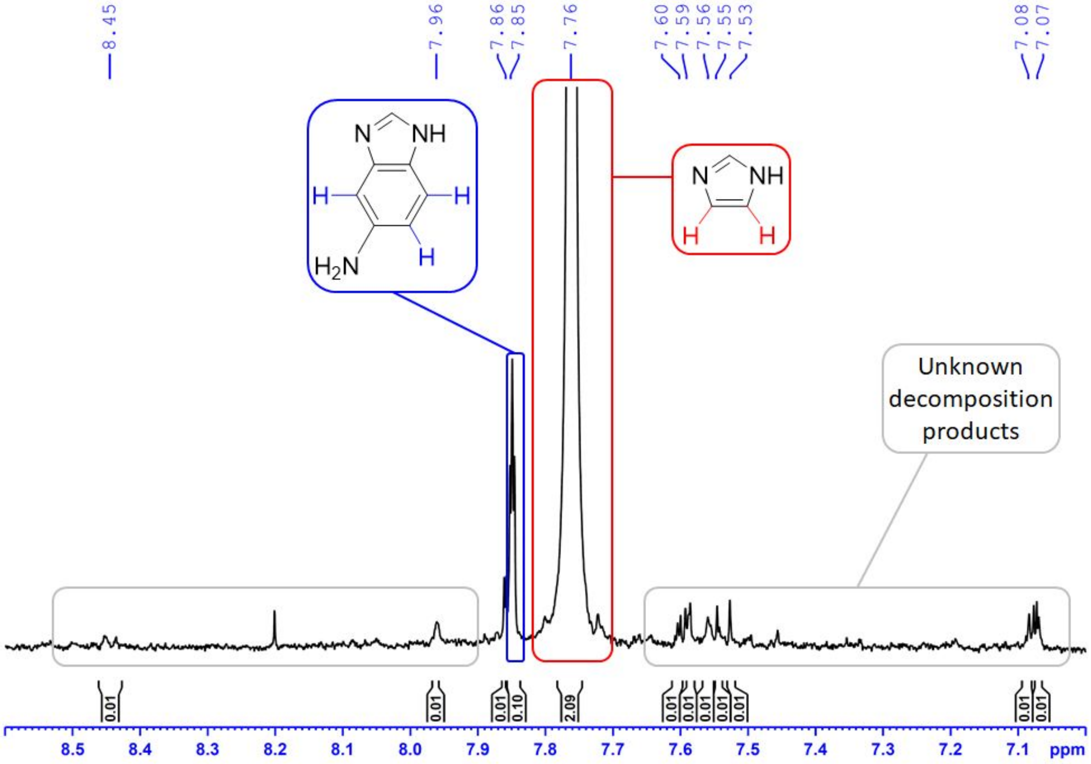

8.45
7.96
7.86
7.85
7.76
7.60
7.59
7.56
7.55
7.53
7.08
7.07
N
NH
H
H
H2N
H
N
NH
H
H
Unknown
decomposition
products
0.01
0.01
0.01
0.10
2.09
0.01
0.01
0.01
0.01
0.01
0.01
0.01
8.5 8.4 8.3 8.2 8.1 8.0 7.9 7.8 7.7 7.6 7.5 7.4 7.3 7.2 7.1 ppm

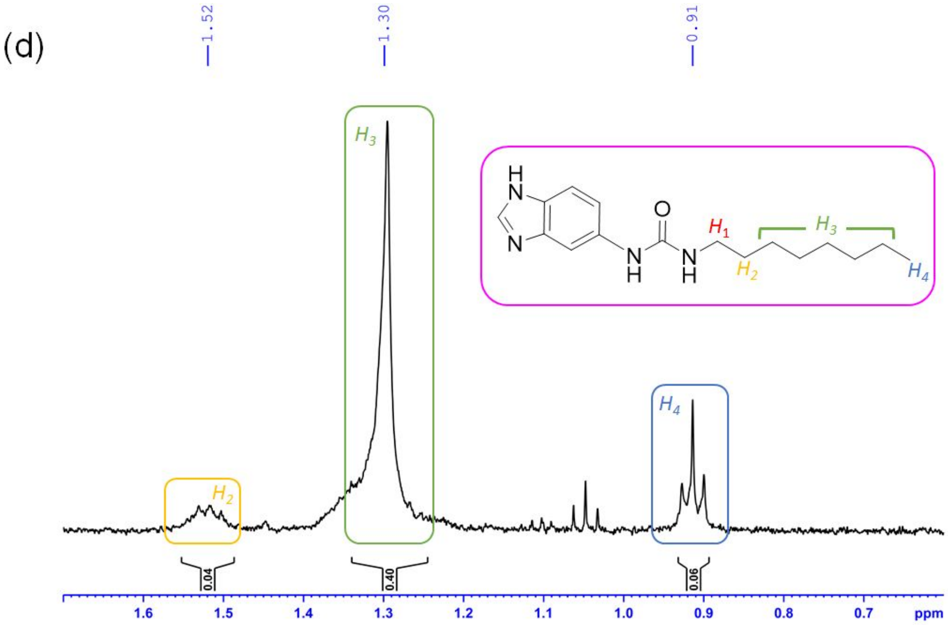

Figure S37: ^{1}H nuclear magnetic resonance spectrum of modified ZIF-UC-6 post 450 °C. (a) Full ^{1}H NMR spectrum with positions of figures (b), (c) and (d) highlighted in grey. (b) Imidazole proton region of ^{1}H NMR. (c) Aromatic region of ^{1}H NMR. Molecules shown in figures (b) and (c) are imidazole – red and 5-aminobenzimidazole – blue. Unknown decomposition product peaks are highlighted in grey. (d) Alkyl chain region of ^{1}H NMR. Proton environments shown are H_2 – yellow, H_3 – green and H_4 – blue. δH (500 MHz; DCl(35%)/D_2O:DMSO-d_6 (dilute conditions); Me_4Si) 9.28 (1H, s, H_a), 9.19 (1H, s, H_b), 8.45-7.07 (aromatic protons) 7.76 (2H, s, Im – aromatic proton), 4.11 (H_2O/HCl), 2.56 (DMSO), 1.52 (2H, m, H_2), 1.30 (10H, m – methylene envelope, H_3), 0.91 (3H, t, H_4), 0.00 (TMS).

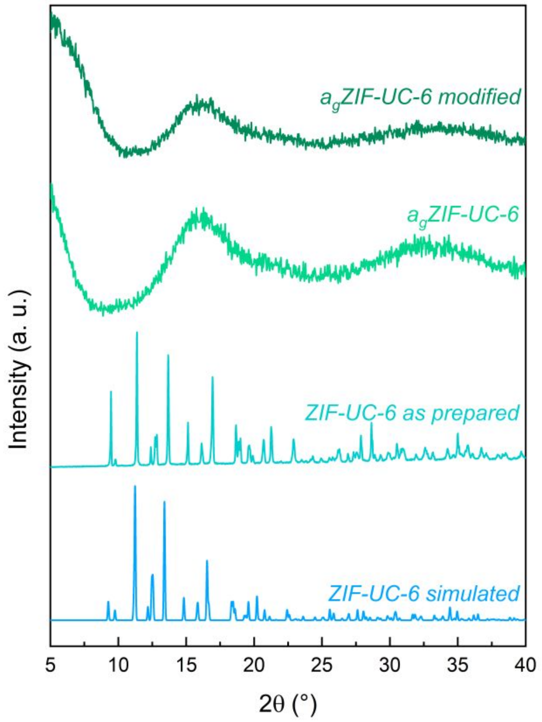

Figure S38: Powder X-ray diffraction patterns between 5-40° of as prepared ZIF-UC-6 (cyan), a_gZIF-UC-6 (light green, only diffuse scattering is observed) and, modified a_gZIF-UC-6 (dark green, only diffuse scattering is observed) compared to the simulated diffraction pattern of ZIF-UC-6 (light blue) obtained from CIF reported in this work.

Table S10: CHN microanalysis of a_gZIF-UC-6

Sample	Mass (mg)	wt.% C	wt.% H	wt.% N
a_gZIF-UC-6	1.1797	37.98	3.05	26.55
a_gZIF-UC-6	1.0076	37.9	2.97	26.62
Mean (ESD)	-	37.94 (0.06)	3.01 (0.06)	26.59 (0.05)
Predicted	-	38.21	3.12	27.72

Table S11: CHN microanalysis of modified a_gZIF-UC-6

Sample	Mass (mg)	wt.% C	wt.% H	wt.% N
a_gZIF-UC-6 PSM	1.3073	37.54	2.83	26.35
a_gZIF-UC-6 PSM	1.3206	37.5	2.82	26.37
a_gZIF-UC-6 PSM	1.5507	37.62	2.87	26.43
Mean (ESD)	-	37.55 (0.06)	2.84 (0.03)	26.38 (0.04)
Predicted	-	38.66	3.19	27.45

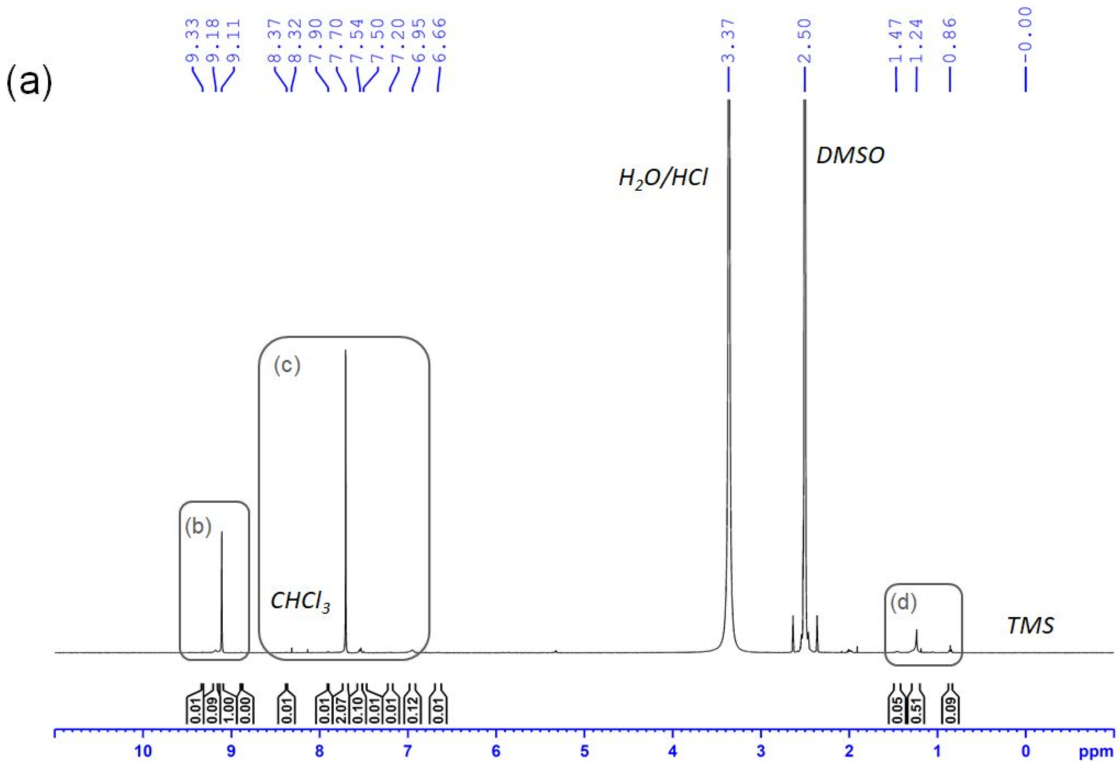

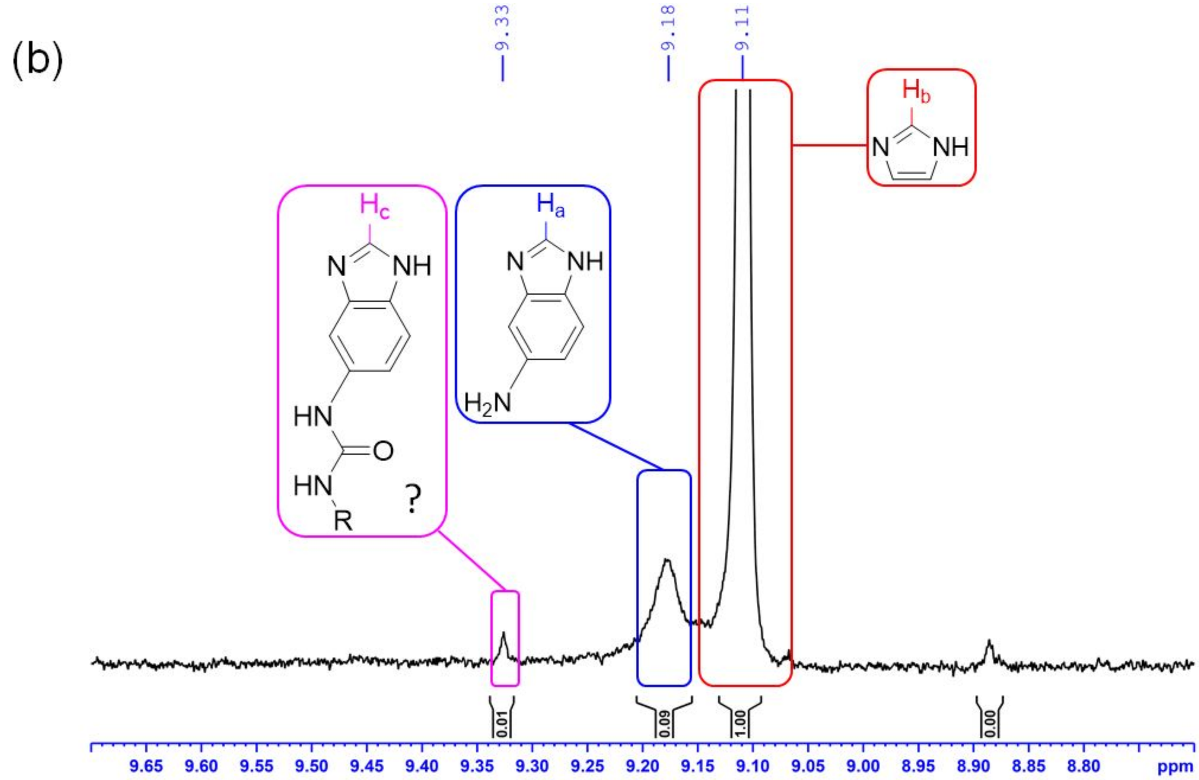
(b)
9.33
9.18
9.11
Hc
NH
N
HN
O
HN
R
?
Ha
N
NH
H2N
Hb
N
NH
0.01
0.09
1.00
0.00
9.65 9.60 9.55 9.50 9.45 9.40 9.35 9.30 9.25 9.20 9.15 9.10 9.05 9.00 8.95 8.90 8.85 8.80 ppm

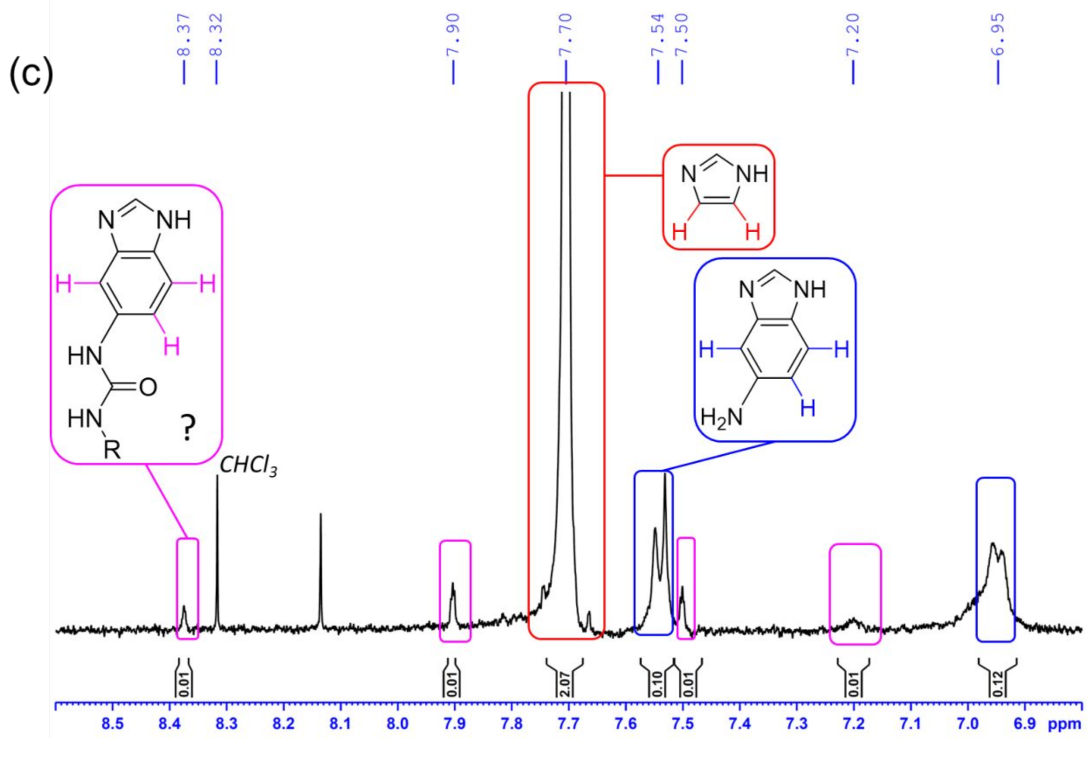
(c)
8.37
8.32
7.90
7.70
7.54
7.50
7.20
6.95
N
NH
H
H
H
HN
O
HN
R
?
CHCl3
N
NH
H
H
H
H2N
0.01
0.01
2.07
0.10
0.01
0.01
0.12
8.5 8.4 8.3 8.2 8.1 8.0 7.9 7.8 7.7 7.6 7.5 7.4 7.3 7.2 7.1 7.0 6.9 ppm

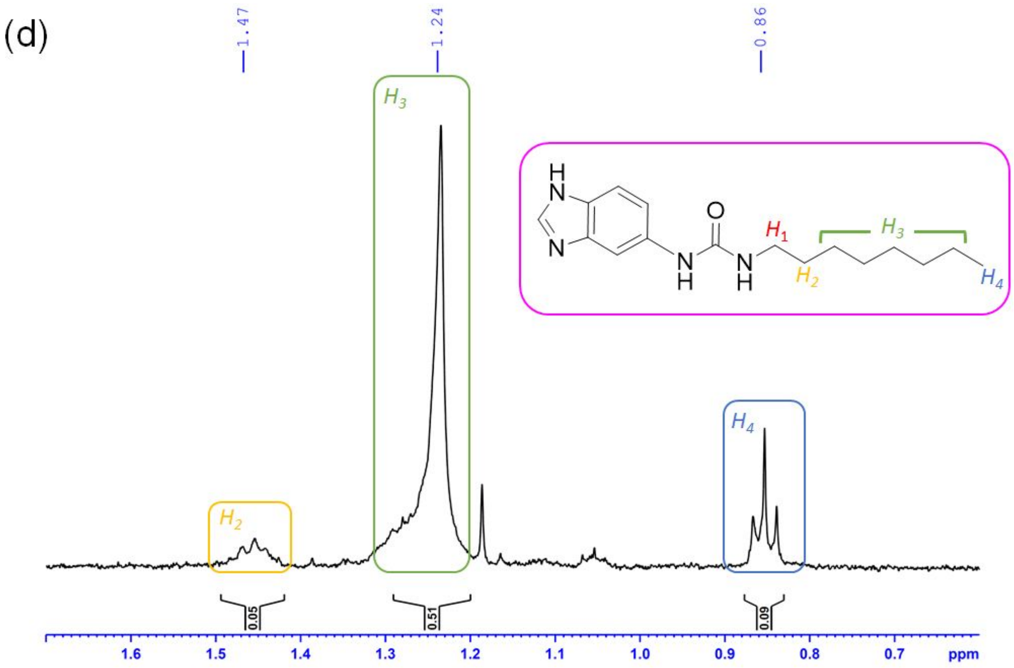

Figure S39: ^{1}H nuclear magnetic resonance spectrum of modified a_gZIF-UC-6. (a) Full ^{1}H NMR spectrum with positions of figures (b), (c) and (d) highlighted in grey. (b) Imidazole proton region of ^{1}H NMR. (c) Aromatic region of ^{1}H NMR. Molecules shown in figures (b) and (c) are imidazole – red and 5-aminobenzimidazole – blue. Peaks with the correct position and integration for the modified linker are highlighted in pink although it is unlikely that all the peaks highlighted in (c) are due to the modified linker. (d) Alkyl chain region of ^{1}H NMR. Proton environments shown are H_1 – red (masked by H_2O/HCl), H_2 – yellow, H_3 – green and H_4 – blue. δH (500 MHz; DCl(35%)/D_2O:DMSO-d_6 (dilute conditions); Me_4Si) 9.33 (1H, s, H_c), 9.18 (1H, s, H_a), 9.11 (1H, s, H_b), 8.37-7.20 (aromatic protons) 7.70 (2H, s, Im – aromatic proton), 3.37 (H_2O/HCl), 2.50 (DMSO), 1.47 (2H, m, H_2), 1.24 (10H, m – methylene envelope, H_3), 0.86 (3H, t, H_4), 0.00 (TMS).

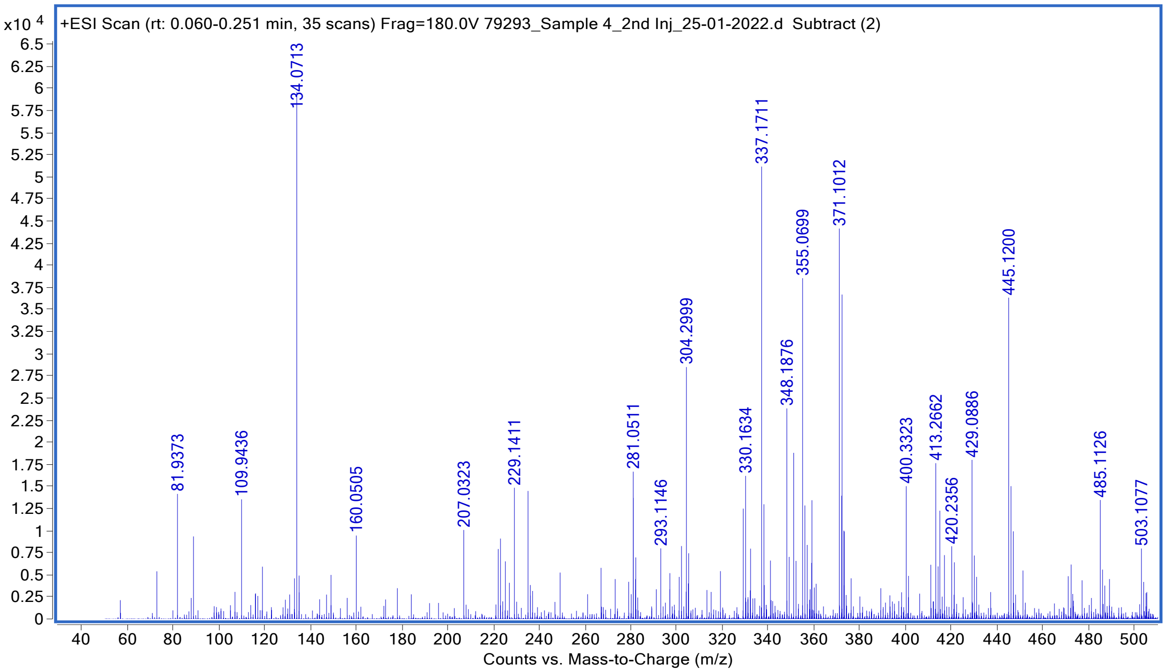

Figure S40: Mass spectrum of digested modified a_gZIF-UC-6 glass. The major peak at m/z = 134.0713 corresponds to unreacted 5-aminobenzimidazole. As the modified linker makes up < 1% of the material, it has a low intensity but is visible below.

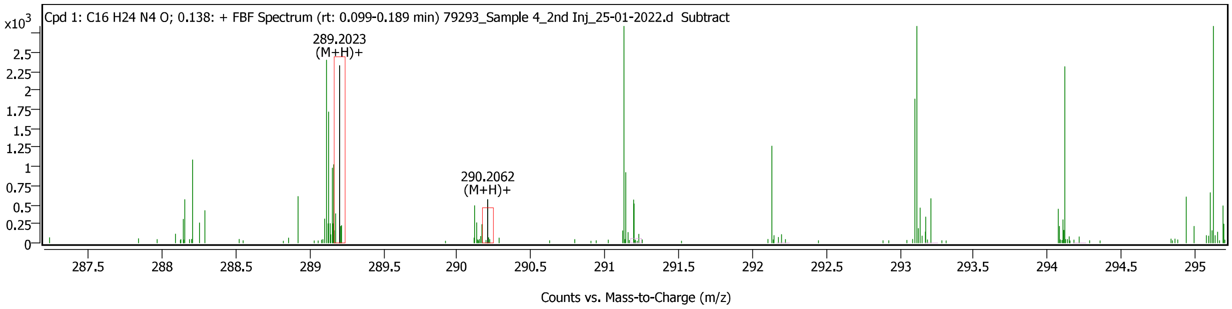

Figure S41: Portion of the mass spectrum of digested modified a_gZIF-UC-6 glass focused on the molecular ion peak for the modified linker, which has formula $C_{16}H_{24}N_4O$ $[C_{16}H_{24}N_4O]H^+$ (calc: *m/z* = 289.2028; found: *m/z* = 289.2023; 2.0 ppm).

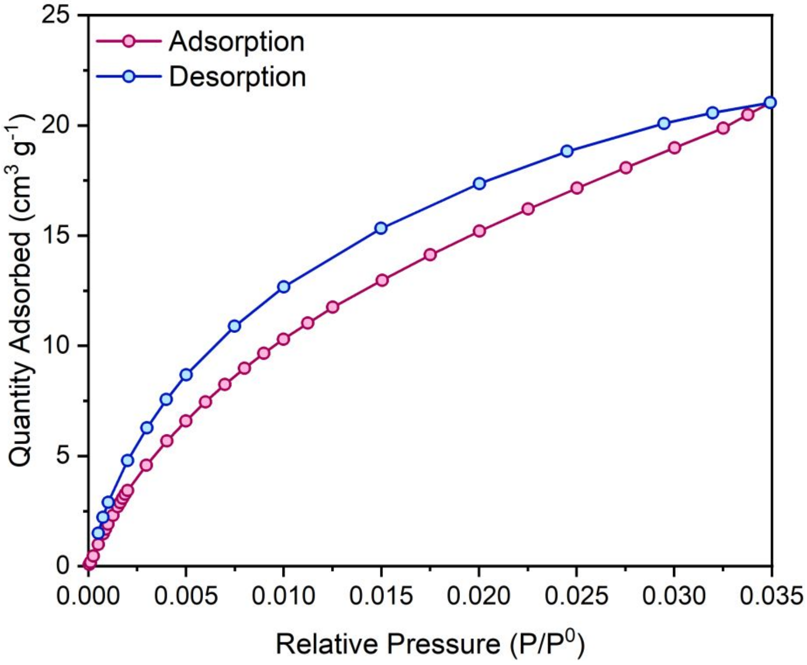

Figure S42: CO_2 adsorption (pink) and desorption (blue) isotherms collected on modified a_gZIF-UC-6 from 0-0.035 P/P^0. Maximum gas uptake was determined using the Micromeritics MicroActive software package.[11]

Table S12: CO_2 gas sorption results for modified a_gZIF-UC-6

Maximum CO_2 uptake (cm^3 g^{-1} STP)	21.03
Maximum CO_2 uptake (mmol g^{-1})	0.94

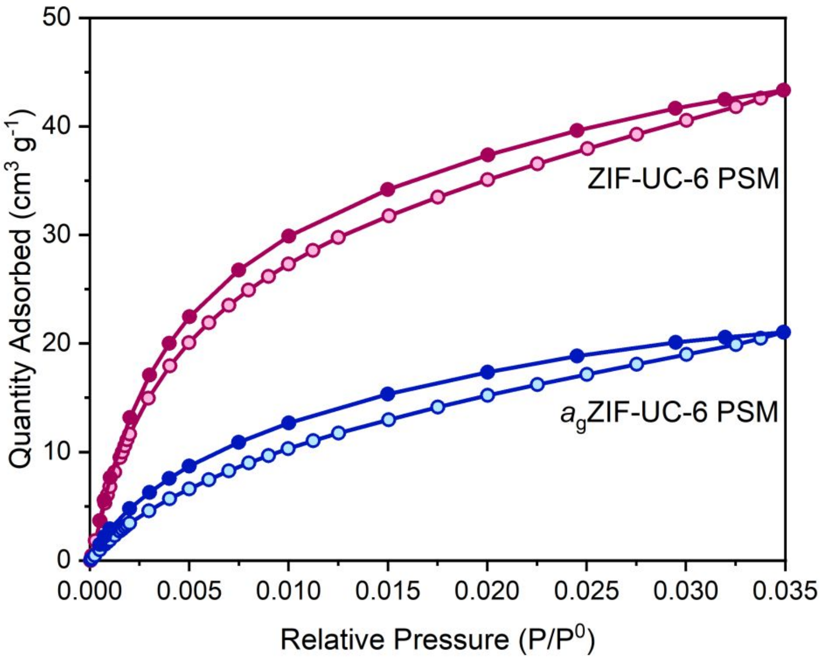

Figure S43: CO_2 gas sorption isotherms for modified ZIF-UC-6 (pink) and modified a_gZIF-UC-6 (blue) plotted on the same graph for comparison. Adsorption isotherms represented by open circles whilst desorption isotherms represented by closed circles.

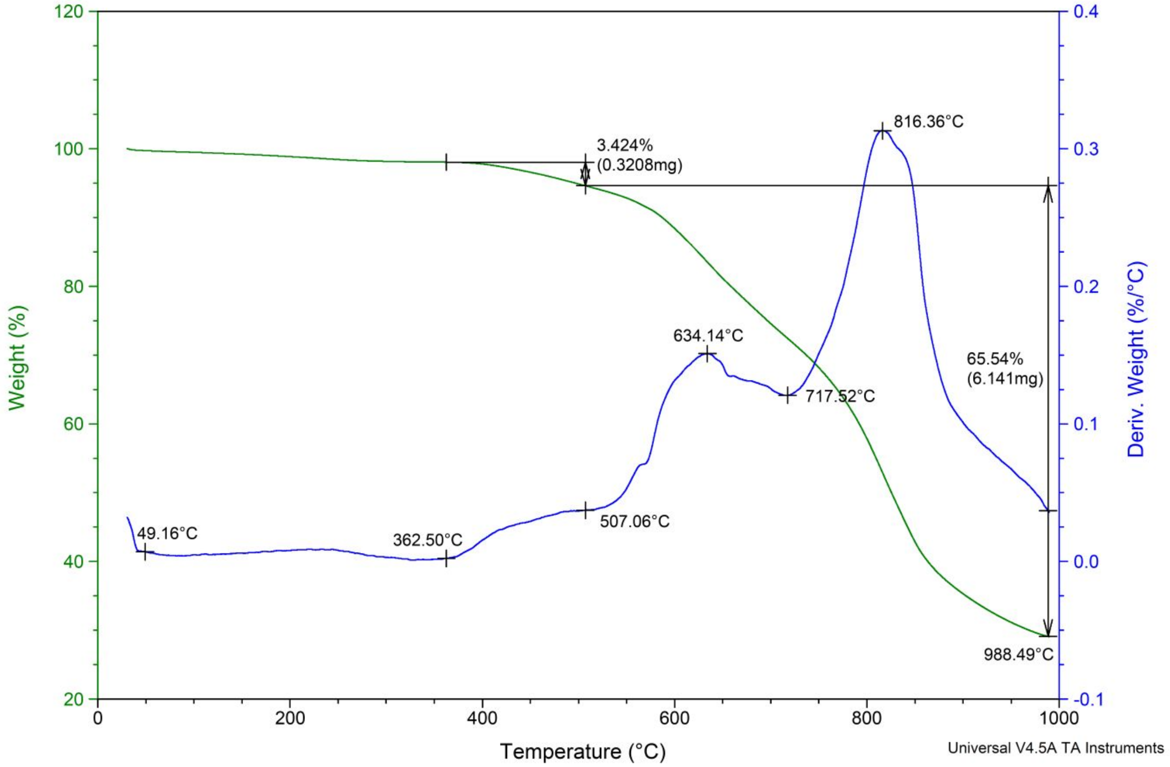

Figure S44: Thermogravimetric analysis of modified a_gZIF-UC-6 heated at 10 °C min^{-1} up to 1000 °C under argon. Weight (%) curve shown in green and derivative weight (%/°C) shown in blue.

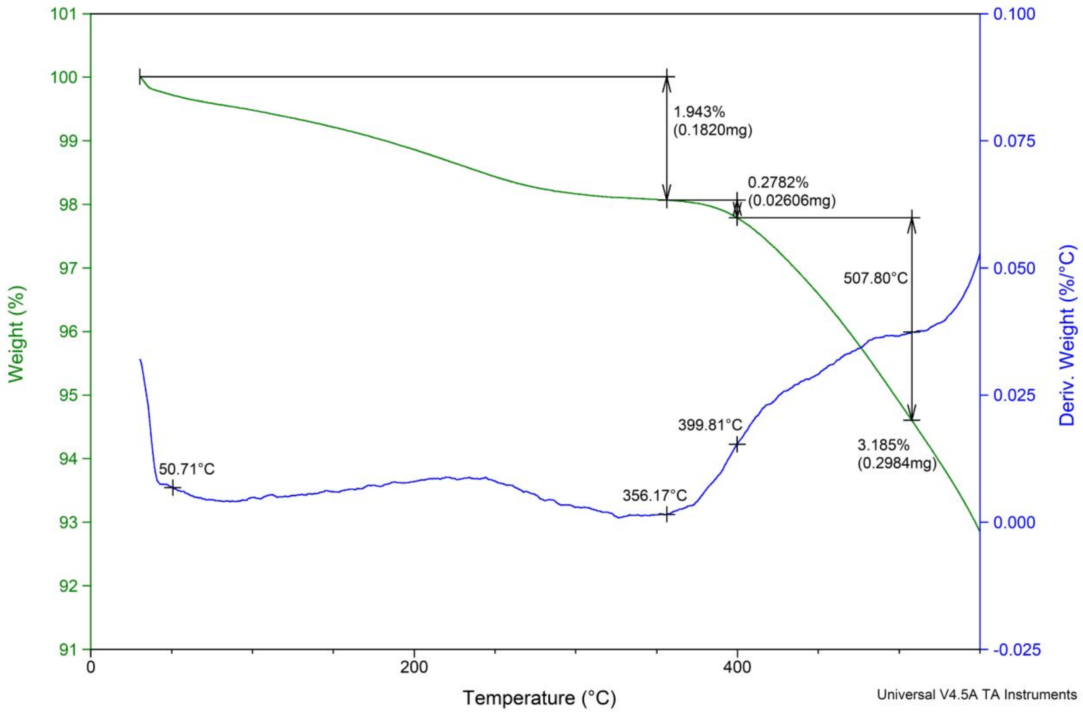

Figure S45: Thermogravimetric analysis of modified a_gZIF-UC-6 heated at 10 °C min^{-1} up to 550 °C under argon. Weight (%) curve shown in green and derivative weight (%/°C) shown in blue.

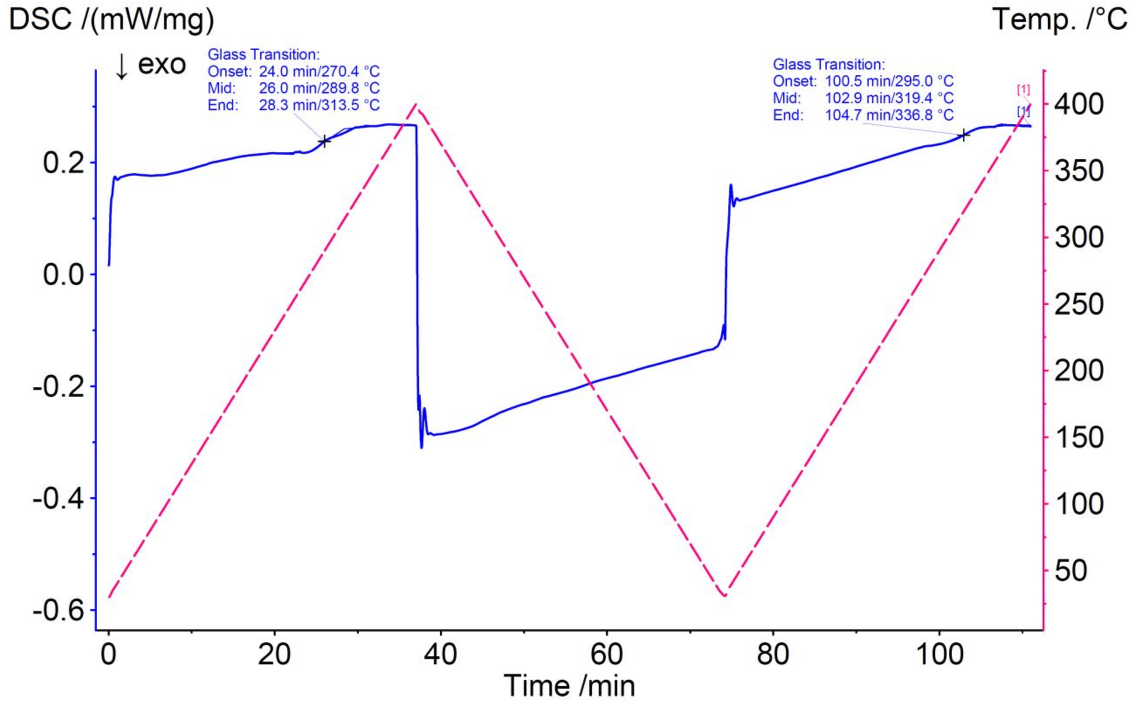

Figure S46: Differential scanning calorimetry of modified a_gZIF-UC-6, heated to 400 °C, cooled to 30 °C and heated to 400 °C at 10 °C min^{-1} under argon. Heat flow curve shown in blue and temperature trace shown in pink.

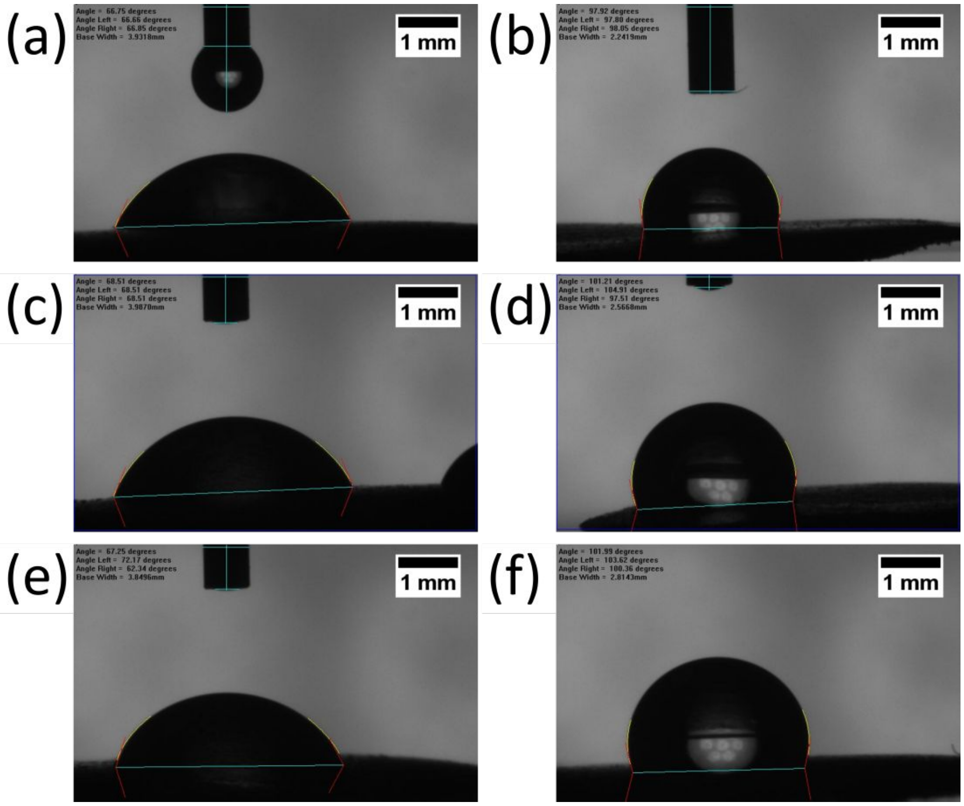

Figure S47: Microscope images of contact angle made between a water droplet and the glass surface. Images overlayed with analysis performed using FTA32 software.[15] (a), (c) and (e) show images of the unmodified glass surface at 3 different positions whilst (b), (d) and (f) show images of the modified glass at 3 different positions on the surface.

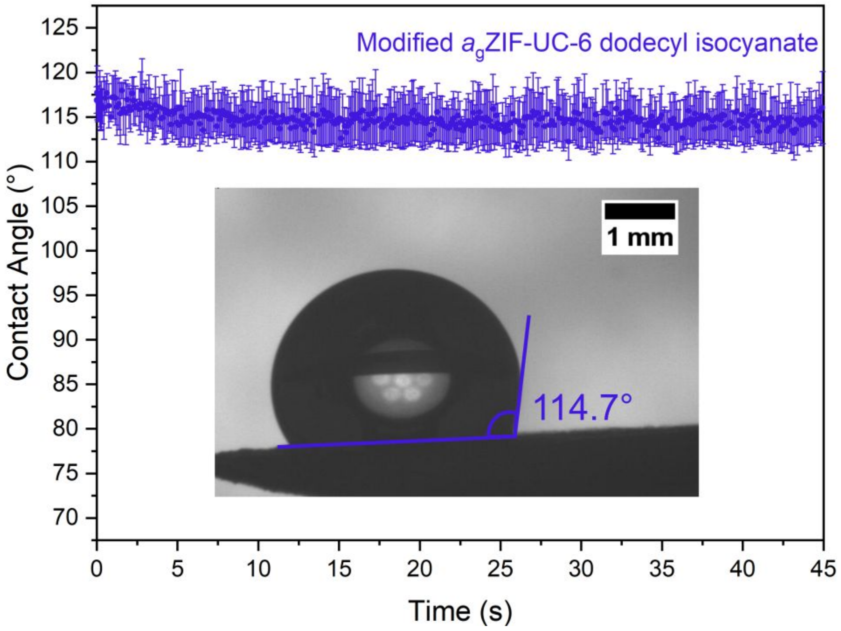

Figure S48: Water contact angle data collected on modified a_gZIF-UC-6 with dodecyl isocyanate (blue) collected over 45 s. Inset: microscope image of water on the modified glass surface showing overall mean contact angles of 114.7 ± 2.7°. Lines and angles drawn on the image are a guide only.

References

(1) Schneider, C. A.; Rasband, W. S.; Eliceiri, K. W. NIH Image to ImageJ: 25 Years of Image Analysis. *Nat. Methods* **2012**, *9* (7), 671–675.

(2) Kourkoumelis, N. PowDLL, a Reusable .NET Component for Interconverting Powder Diffraction Data: Recent Developments. In *ICDD Annual Spring Meetings*; O’Neill, L., Ed.; Cambridge University Press: Powder Diffraction, 2013; Vol. 28, pp 137–148.

(3) Coelho, A. A. TOPAS and TOPAS-Academic: An Optimization Program Integrating Computer Algebra and Crystallographic Objects Written in C++. *J. Appl. Crystallogr.* **2018**, *51* (1), 210–218.

(4) Kubelka, P.; Munk, F. An Article on Optics of Paint Layers. *Z. Tech. Phys.* **1931**, *12*, 593–601.

(5) Kubelka, P. New Contributions to the Optics of Intensely Light-Scattering Materials. *J. Opt. Soc. Am.* **1948**, *38* (5), 448–

(6) *Bruker TopSpin 4.0.7*; Bruker: Billerica, Massachusetts, 2019. https://www.bruker.com/en/products-and-solutions/mr/nmr-software/topspin.html (accessed 2021-09-24).

(7) *TA Instruments Universal Analysis*; TA Instruments: New Castle, Delaware, 2000. https://www.tainstruments.com/installation-of-universal-analysis-from-the-advantage-software-package/ (accessed 2021-09-24).

(8) *Netzsch Proteus – Thermal Analsyis – Version 8.0.2*; NETZSCH-Gerätebau GmbH: Selb, Germany, 2020. https://www.netzsch-thermal-analysis.com/en/products-solutions/software/proteus/ (accessed 2021-09-24).

(9) Bumstead, A. M.; Rios Gomez, M. L.; Thorne, M. F.; Sapnik, A. F.; Longley, L.; Tuffnell, J. M.; Keeble, D. S.; Keen, D. A.; Bennett, T. D. Investigating the Melting Behaviour of Polymorphic Zeolitic Imidazolate Frameworks. *CrystEngComm* **2020**, *22*, 3627–3637.

(10) Liu, M.; McGillicuddy, R. D.; Vuong, H.; Tao, S.; Slavney, A. H.; Gonzalez, M. I.; Billinge, S. J. L.; Mason, J. A. Network-Forming Liquids from Metal-Bis(Acetamide) Frameworks with Low Melting Temperatures. *J. Am. Chem. Soc.* **2021**, *143* (7), 2801–2811.

(11) MicroActive Version 4.04; Micromeritics Instrument Corporation: Norcross, Georgia, 2017. https://www.micromeritics.com/microactive-reporting-software-dft/ (accessed 2021-09-24).

(12) Soper, A. K.; Barney, E. R. Extracting the Pair Distribution Function from White-Beam X-Ray Total Scattering Data. *J. Appl. Crystallogr.* **2011**, *44* (4), 714–726.

(13) Soper, A. K. *GudrunN and GudrunX: Programs for Correcting Raw Neutron and X-Ray Diffraction Data to Differential Scattering Cross Section*; Technical Report RAL-TR-2011-013; Science and Technology Facilities Council, 2011. https://epubs.stfc.ac.uk/manifestation/6710/RAL-TR-2011-013.pdf (accessed 2021-09-24).

(14) Keen, D. A. A Comparison of Various Commonly Used Correlation Functions for Describing Total Scattering. *J. Appl. Crystallogr.* **2001**, *34* (2), 172–177.

(15) Woodward, R. *Fta32 Version 2.1*; First Ten Angstroms, Inc.: Newark 2015. https://www.firsttenangstroms.com/copy-of-additional-product-info-she (accessed 2021-09-24).
